# Outcome-Specific Efficacy of Different Probiotic Strains and Mixtures in Irritable Bowel Syndrome: A Systematic Review and Network Meta-Analysis

**DOI:** 10.3390/nu15173856

**Published:** 2023-09-04

**Authors:** Peiwei Xie, Mei Luo, Xuehong Deng, Jiahui Fan, Lishou Xiong

**Affiliations:** Department of Gastroenterology, The First Affiliated Hospital of Sun Yat-Sen University, Guangzhou 510080, China

**Keywords:** irritable bowel syndrome, probiotic, network meta-analysis, outcome, efficacy

## Abstract

Irritable bowel syndrome (IBS) is a common gastrointestinal disease. The efficacy of different probiotics in treating IBS remains controversial. This network meta-analysis aimed to compare and rank the outcome-specific efficacy of different probiotic strains or combinations in adults with IBS. We searched the literature up to June 2023. Randomized controlled trials (RCTs) that evaluated the efficacy of probiotics in IBS were included. A frequentist framework was used to perform this study. In total, 9253 participants from 81 RCTs were included in the study. Four probiotic strains and five mixtures were significantly superior to placebo in improving IBS Symptom Severity Scale, among which *Lactobacillus acidophilus* DDS-1 ranked first (surface under the cumulative ranking, SUCRA, 92.9%). A mixture containing five probiotics (SUCRA, 100%) ranked first in improving the IBS-Quality of life. *Bacillus coagulans* MTCC 5856 (SUCRA, 96.9%) and *Bacillus coagulans* Unique IS2 (SUCRA, 92.6%) were among the most effective probiotics for improving abdominal pain. Three probiotic strains and two mixtures were effective in alleviating abdominal bloating. Four probiotic strains and a mixture were significantly superior to placebo in reducing the bowel movement frequency in diarrhea-predominant IBS (IBS-D). *Bacillus coagulans* MTCC 5856 (SUCRA, 99.6%) and *Saccharomyces cerevisiae* CNCM I-3856 (SUCRA, 89.7%) were among the most effective probiotics for improving the Bristol stool form scale of IBS-D. Only some probiotics are effective for particular outcomes in IBS patients. This study provided the first ranking of outcome-specific efficacy of different probiotic strains and combinations in IBS. Further studies are needed to confirm these results.

## 1. Introduction

Irritable bowel syndrome (IBS) is a common functional bowel disease that is induced by disorders of gut–brain interactions. Typical symptoms of IBS include recurrent abdominal pain associated with changes in stool form or frequency [1]. The prevalence of IBS is approximately 9.2% globally, but varies from 1.1% to 35.5% according to the region and diagnostic criteria [2,3]. The annual healthcare cost estimates of IBS are substantial: CNY 123 billion in China, USD 10 billion in the USA, and GBP 2 billion in the UK [4,5,6,7]. IBS exerts a great impact on quality of life and productivity for individuals [8]. Patients with IBS experience troublesome and unpredictable symptoms, which cause frequent medical visits and absenteeism [9,10]. Consistent health worries and a lack of understanding by family may lead to psychological problems in patients, such as anxiety and depression [10]. IBS imposes a huge burden on individuals and society.

The gut microbiota, which is considered the ecologic system of various microorganisms in the gastrointestinal tract, plays a critical role in the pathogenesis of IBS through the gut–brain axis [11]. Altering the composition of gut microbiota toward a healthy community has become a potential strategy for IBS treatment [12,13]. One of the representative choices of this strategy is probiotics, which are live microorganisms that confer a health benefit on the host when administered in adequate amounts [14]. The efficacy of particular species of probiotics in IBS has been reported in randomized controlled trials (RCTs) and meta-analyses. A recent RCT confirmed that *Bifidobacterium* quadruple viable tablets effectively alleviated abdominal pain and diarrhea for patients with diarrhea-predominant IBS (IBS-D) [15]. Two meta-analyses indicated that probiotics had beneficial effects on abdominal pain and bloating [16,17].

However, it is still unclear which strain or combination of probiotics are effective in global IBS symptoms. It is even harder for physicians to select appropriate probiotics for IBS patients with various symptoms. Numerous single-strain probiotics and multistrain combination products have been developed for IBS every year. Instead of comprehensive evaluations from a standard system, reports from some clinical trials have focused on significant efficacy and specific outcomes in endorsements of particular probiotics [18]. The profusion of data regarding different strains or combinations of probiotics, different IBS subtypes, and different end points, outcomes, and study quality has resulted in a complex evidence network that is difficult to interpret [19]. Hence, a previous meta-analysis made conservative and cautious estimates of the efficacy of probiotics [20]. Relevant guidelines also present different attitudes toward probiotics. The British Society of Gastroenterology (BSG) guidelines set probiotics as first-line treatments but have not yet recommended a specific species or strain [12]. However, the American College of Gastroenterology (ACG) guidelines suggest against probiotics for the treatment of global IBS symptoms [18]. More detailed evidence of probiotic efficacy in IBS is needed.

In this systematic review and network meta-analysis (NMA), we aimed to evaluate the comparative efficacy of different probiotic strains and mixtures based on global conditions, mental health levels, and specific gastrointestinal symptoms.

## 2. Materials and Methods

This NMA was performed according to the Preferred Reporting Items for Systematic Reviews and Meta-analysis Statement (the PRISMA 2020 statement and the PRISMA Extension Statement of NMA) [21,22]. We registered a protocol (CRD42023387351) on the International Prospective Register of Systematic Reviews. Alterations of the original protocol were presented in Supplementary Text S1.

### 2.1. Search Strategy and Eligibility Criteria

We performed comprehensive literature searches of five databases from their inception to 1 June 2023 (Web of Science, PubMed, ClinicalTrials.gov, Cochrane Central Register of Controlled Trials, and Embase). No language restrictions were applied. The basic search strategies were as follows: ((((irritable bowel syndrome [Title/Abstract]) OR (IBS [Title/Abstract])) OR (irritable bowel syndrome [MeSH Terms])) AND (((((((((Probiotic* [Title/Abstract]) OR (Probiotic [MeSH Terms])) OR (*Lactobacillus* [Title/Abstract])) OR (*Saccharomyces* [Title/Abstract])) OR (*Bacillus* [Title/Abstract])) OR (*Bifidobacterium* [Title/Abstract])) OR (*Clostridium* [Title/Abstract])) OR (*Streptococcus* [Title/Abstract])) OR (*Enterococcus* [Title/Abstract]))) AND ((((((clinical trial [Title/Abstract]) OR (clinical trials [MeSH Terms])) OR (clinical trial [Publication Type])) OR (random* [Title/Abstract])) OR (random allocation [MeSH Terms])) OR (therapeutic use [MeSH Subheading])). Supplementary Text S2 presents the detailed search strategies in different databases. We also screened meta-analyses about IBS treatments published in the last five years for eligible clinical trials. The official websites of academic conferences (Digestive Disease Week, American College of Gastroenterology, Asian Pacific Digestive Week and the United European Gastroenterology Week) and probiotic companies were searched for potential ongoing studies and grey literature. These three approaches constitute the literature source for the NMA.

The inclusion criteria were as follows:

1. RCTs that compared the efficacy of probiotics with placebo or different probiotics for IBS;
2. Participants were diagnosed with IBS based on Rome I, II, III, or IV criteria, Manning criteria, or physician’s opinion;
3. Patients in the test group received single or multistrain probiotics;
4. Patients in the control group received placebo or another probiotic;
5. RCTs should report at least one of the targeted outcomes (see 2.2 Outcome assessment). Outcomes should be reported as data at baseline and endpoint, or as absolute changes during the study;
6. The treatment duration was at least two weeks.

The exclusion criteria were as follows:

1. Open-label trials, single-arm studies, nonrandomized trials, reviews, protocols, and letters. Crossover RCTs that did not report data from the first stage were excluded;
2. Duplicate study;
3. Studies involving pregnant or lactating mothers, patients with a history of gastrointestinal surgery, and patients aged < 18 years;
4. Studies involving patients who received combined treatments, such as synbiotics, antibiotics, antidepressants, and psychological therapy.

### 2.2. Outcome Assessment

We selected seven outcomes to evaluate the global condition, mental health condition, and core gastrointestinal symptoms of patients with IBS. All data on these outcomes were recorded as the change from baseline to therapy completion.

The global condition of IBS was evaluated based on the following outcomes. (1) Change in IBS Symptom Severity Scale (IBS-SSS) from baseline [23]. The five domains of the IBS-SSS generate a total score from 0 to 500 (no symptoms to very severe). (2) The change in IBS-Quality of Life (IBS-QOL) from baseline [24]. The total score of 34 items in the IBS-QOL generates a total score from 0 to 100. Higher scores indicate a better IBS quality of life.

(3) The mental health condition of patients with IBS was evaluated by the change in the Hospital Anxiety and Depression Scale (HADS) from baseline [25]. The HADS-total, anxiety, and depression scores were collected individually.

Core gastrointestinal symptoms of IBS: (4) Abdominal pain score, reflecting the degree of abdominal pain severity. (5) The abdominal bloating score reflects the severity of abdominal bloating. This outcome was defined as a feeling of abdominal swelling or gas accumulation. The terms “bloating” or “distension” were used in related questionnaires. (6) Bowel movement frequency (per week) in IBS-D or IBS with predominant constipation (IBS-C). (7) Bristol stool form scale in IBS-D or IBS-C. Data on adverse events in each study were collected.

Single-strain probiotics were classified at the strain level. The classification of multistrain probiotics consists of several cornerstone probiotics and “X” probiotics. X can be absent or presented in other probiotics, usually fewer than two strains. Alterations in the gut microbiota after probiotic administration were summarized for a systematic review, including changes in microbiota abundance and diversity.

### 2.3. Data Extraction

Pairs of reviewers independently screened titles and abstracts of the search results. The full texts and study protocols of potentially eligible articles were examined based on eligibility criteria. Two reviewers used a piloted electronic form to extract the data independently and in duplicate (Supplementary Text S3). Data extraction included the following items: RCT general information (article title, first author, publication year, trial location, trial design, inclusion and exclusion criteria, sample size, composition and dosage of probiotics, and follow-up period), population characteristics (IBS diagnosis criteria, IBS duration and severity, sex, age, and body mass index), and outcomes of interest. Intention-to-treat analyses were performed for data collection. Disagreements were resolved by consensus with a senior investigator. Corresponding authors were queried for original data by e-mail if the outcome data were not reported in full text.

### 2.4. Risk of Bias and Evidence Quality

The Cochrane risk-of-bias tool version 2 (RoB 2) was used to assess the risk of bias for each outcome in the selected studies [26]. The tool comprises several questions and algorithms that map responses to signaling questions to a proposed risk-of-bias judgment. Two authors independently assessed risk of bias. The Grading of Recommendations, Assessment, Development and Evaluation (GRADE) system was applied to assess the quality of this systematic review and NMA for each outcome [27]. Disagreements were resolved by consensus.

### 2.5. Statistical Analysis

Traditional pairwise meta-analyses were conducted using a random-effects model [28]. The mean difference (MD) with 95% confidence intervals (CIs) was calculated to measure the treatment effects of continuous data. Standardized mean difference (SMD) was used for abdominal pain and bloating scores. Heterogeneity was described using the *I*^2^ statistic [29].

Random-effects NMA was conducted by using the frequentist framework. The MD or SMD with 95% CIs for outcomes was calculated [30]. Heterogeneity among the included studies was assessed using a prediction interval plot. It showed the influence of heterogeneity by providing a predicted range for the true treatment effect in 95% of individual studies [31]. A fundamental assumption of NMA is transitivity, which requires study sets to be similar enough in critical clinical characteristics. Regarding this NMA, the characteristics included the proportion of females and the average age. These effect modifiers were presented using box plots. The consistency was evaluated when a loop was presented in the evidence network. We evaluated local inconsistency using node-splitting analysis and loop-specific analysis [30,32]. Global inconsistency was assessed by an inconsistency model of design-by-treatment interaction [33].

Network plots were constructed to visualize the treatment network at the outcome level. The surface under the cumulative ranking curve (SUCRA) values were calculated for efficacy ranks. The SUCRA value of a treatment indicates the chance of this treatment to be the best [30]. Publication bias was assessed by funnel plots [30].

The above analyses used code packages (*mvmeta*, *network*, and *network graphs*) based on STATA (Stata Corp, College Station, TX, USA) [34,35].

### 2.6. Efficacy Classification

We referred to a novel and succinct approach to efficacy classification proposed by Morgan et al., and the interventions were categorized into three levels as follows [36].

- Level A (among the most effective): probiotics that are significantly superior to placebo and at least one probiotic at Level B;
- Level B: probiotics that are more effective than placebo, but not superior to any other probiotic(s) superior to placebo;
- Level C (among the least effective): probiotics with no significant difference compared with placebo.

## 3. Results

**Figure 1:**
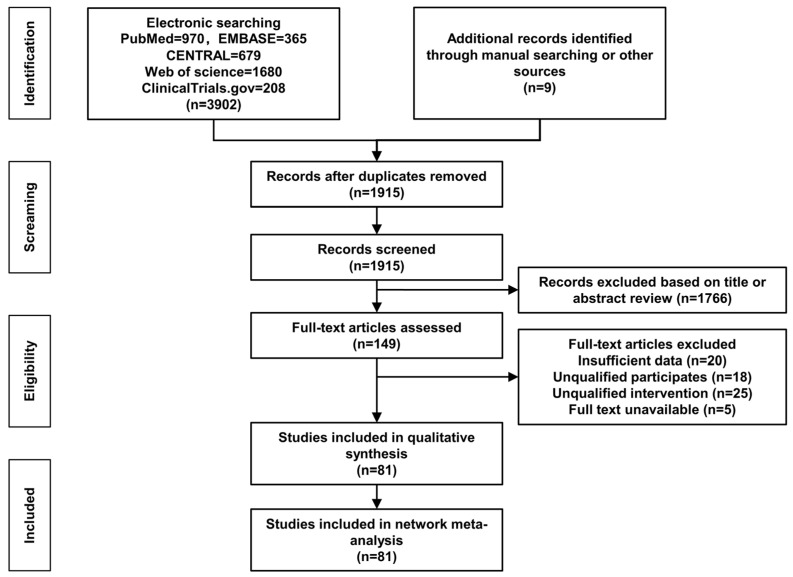
Flow chart illustrating the procedures of literature search and refinement.

The literature search and refinement procedure are illustrated in Figure 1. Electronic and manual searches yielded 3903 initial records. After removing duplicates and reviewing the titles and abstracts, we evaluated the full texts of 149 articles. Ultimately, the qualitative synthesis and network meta-analysis included 81 RCTs. The references to the RCTs included in this NMA are listed in Supplementary Text S4.

### 3.1. Characteristics of Included Studies

**Figure 2:**
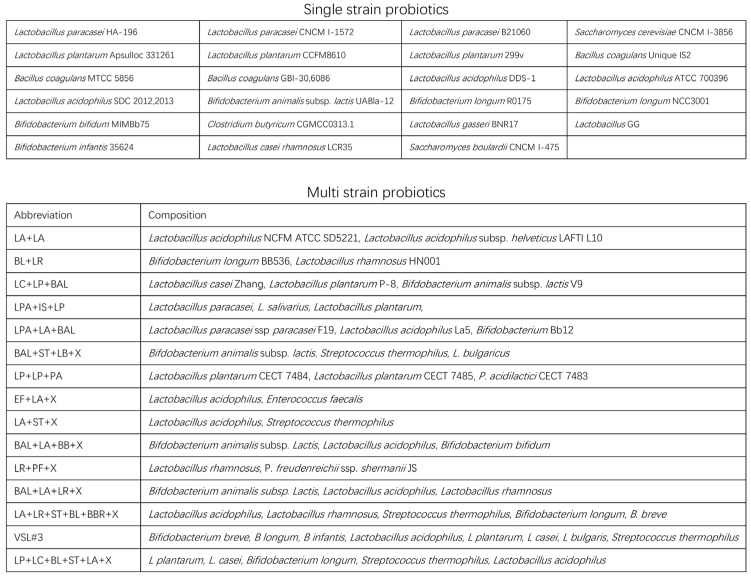
Single strain and multistrain probiotics included in network meta-analyses.

The characteristics of the included RCTs are summarized in Tables S1 and S2. This NMA included 9253 participants from 81 RCTs [15,37,38,39,40,41,42,43,44,45,46,47,48,49,50,51,52,53,54,55,56,57,58,59,60,61,62,63,64,65,66,67,68,69,70,71,72,73,74,75,76,77,78,79,80,81,82,83,84,85,86,87,88,89,90,91,92,93,94,95,96,97,98,99,100,101,102,103,104,105,106,107,108,109,110,111,112,113,114,115,116]. Participants in this study came from 25 countries ranging across Europe, North America, Asia, and Africa. The sample size ranged from 19 to 456 patients. The average age ranged from 21.8 to 63 years. Most studies (*n* = 53) involved recruiting all subtypes of IBS, while 18 studies focused on IBS-D and five on IBS-C. The distribution of effect modifiers is summarized in Figure S1.The classification of the included probiotics is summarized in Figure 2.

### 3.2. Risk of Bias within Studies

Twenty-four trials were evaluated as “some concern” on bias arising from the randomization process. Thirty-two trials did not provide sufficient information about deviations from the intended interventions. Three trials were evaluated as “some concern” for bias due to missing outcome data. Two trials were evaluated as “some concern” on bias in measurement of the outcome. Twenty-two trials did not provide details on the selection of reported results. Notably, 45 RCTs in this NMA were funded by commercial companies. The ROB 2 figures for each outcome are shown in Figure S2.

### 3.3. Critical Results of Network Meta-Analysis

The results of the pairwise meta-analysis are summarized in Table S3. League tables for the different outcomes are summarized in Table S4. Network plots are shown in Figure S3. SUCRAs are summarized in Figure S4 and Table S5.

#### 3.3.1. IBS-SSS

**Figure 3:**
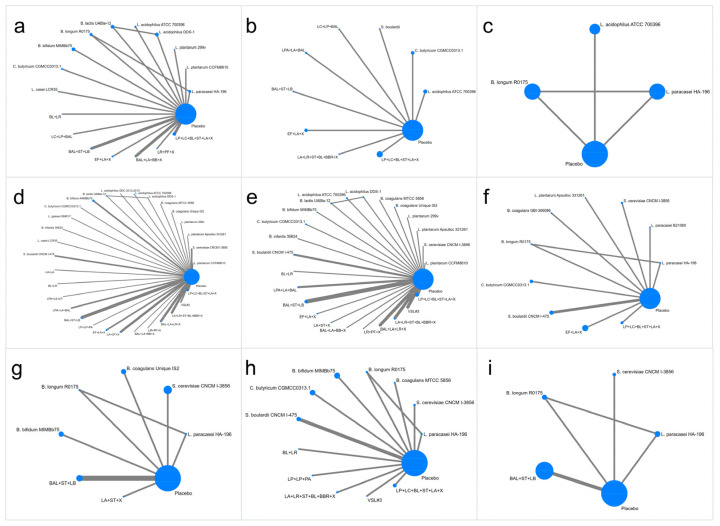
Network plots for different outcomes. (**a**). IBS Symptom Severity Scale; (**b**). IBS-Quality of Life Measure; (**c**). the Hospital Anxiety and Depression Scale; (**d**). abdominal pain score; (**e**). abdominal bloating score; (**f**). bowel movement frequency (per week) in IBS-D; (**g**). bowel movement frequency (per week) in IBS-C; (**h**). Bristol stool form scale in IBS-D; (**i**). Bristol stool form scale in IBS-C. Different probiotics are represented by nodes. The size of each node is proportional to the number of patients. The width of the edges represents the number of RCTs.

The evidence network was constructed with ten single-strain probiotics and seven multistrain groups from 18 RCTs (2628 patients, Figure 3). Moderate-certainty evidence indicated that *Lactobacillus acidophilus* DDS-1 (MD, −77.70; 95% CI, −101.72 to −53.68), BL + LR (MD, −80.99; 95% CI, −130.73 to −31.26), LC + LP + BAL (MD, −76.42; 95% CI, −114.90 to −37.95), LP + LC + BL + ST + LA + X (MD, −63.96; 95% CI, −78.66 to −49.26) and *Bifidobacterium animalis* subsp. *lactis* UABla-12 (MD, −48.80; 95% CI, −73.00 to −24.60) were classified as efficacy level A (Table 1). EF + LA + X, *Bifidobacterium bifidum* MIMBb75, *Clostridium butyricum* CGMCC0313.1, and BAL + LA + BB + X were also significantly superior to the placebo in improving IBS-SSS (efficacy level B, Table 1). The top three treatments based on SUCRAs were *Lactobacillus acidophilus* DDS-1 (92.9%), BL + LR (91.6%), and LC + LP + BAL (90.9%) (moderate certainty).

#### 3.3.2. IBS-QOL

Nine RCTs involving three single-strain probiotics and six multistrain groups reported IBS-QOL (1323 patients, Figure 3). The NMA indicated that LP + LC + BL + ST + LA + X (MD, 15.35; 95% CI, 4.45 to 26.26; moderate certainty) was considered efficacy level A (Table 1). *Clostridium butyricum* CGMCC0313.1 (MD, 4.07; 95% CI, 0.50 to 7.65) was also significantly superior to the placebo in improving IBS-QOL (Table 1).

#### 3.3.3. HADS Score

Evidence networks were constructed with five single-strain probiotics and a multistrain group from five RCTs (622 patients, Figure 3). *Bifidobacterium longum* R0175 significantly reduced the HADS total score (MD, −0.34; 95% CI, −0.48 to −0.20; high certainty). *Bifidobacterium longum* NCC3001 significantly reduced the HADS-depression score (MD, −3.0; 95% CI, −4.92 to −1.08; high certainty) (Table 1). No significant improvement was found in the HADS-anxiety score among the included studies.

#### 3.3.4. Abdominal Pain Score

The abdominal pain score was reported in 47 RCTs (4680 patients) that involved 16 single-strain probiotics and 14 multistrain groups (Figure 3). NMA indicated that *Bacillus coagulans* MTCC 5856 (SMD, −41.80; 95% CI, −61.59 to −22.00; Moderate certainty) and *Bacillus coagulans* Unique IS2 (SMD, −32.00; 95% CI, −45.35 to −18.65; Moderate certainty) were classified as efficacy level A (Table 2). *Lactobacillus gasseri* BNR17, *Lactobacillus plantarum* Apsulloc 331261, *Lactobacillus acidophilus* DDS-1, LPA + LS + LP, *Saccharomyces cerevisiae* CNCM I-3856, VSL#3, EF + LA + X, and LA + ST + X were significantly superior to the placebo in reducing abdominal pain score (Table 2). The top three treatments based on SUCRAs were *Bacillus coagulans* MTCC 5856 (96.9%), *Bacillus coagulans* Unique IS2 (92.6%), and *Lactobacillus gasseri* BNR17 (91.3%).

#### 3.3.5. Abdominal Bloating Score

Thirty-nine studies involving 13 single-strain probiotics and 11 multistrain groups found improvements in abdominal bloating scores (3383 patients, Figure 3). NMA indicated that BL + LR, *Lactobacillus plantarum* CCFM8610, *Lactobacillus plantarum* 299v, VSL#3, *Bifidobacterium bifidum* MIMBb75 were significantly superior to placebo in reducing the abdominal bloating score (Table 2). The top three treatments based on SUCRAs were BL + LR (SMD, −34.00; 95% CI, −56.94 to −11.06; SUCRA 94.5%), *Bacillus coagulans* MTCC 5856 (SMD, −19.92; 95% CI, −34.91 to −4.91; SUCRA 85%), and *Lactobacillus plantarum* CCFM8610 (SMD, −14.79; 95% CI, −29.11 to −0.48; 82.6%).

#### 3.3.6. Bowel Movement Frequency (Per Week) in the IBS-D and IBS-C Groups

The IBS-D evidence network was constructed using eight single-strain probiotics and two multistrain groups from 10 RCTs (877 patients, Figure 3). The NMA indicated that EF + LA + X (MD, −3.95; 95% CI, −5.02 to −2.88; Moderate certainty) were classed as efficacy level A (Table 3). *Lactobacillus paracasei* B21060, *Lactobacillus paracasei* HA-196, *Bacillus coagulans* GBI-306086, and *Bifidobacterium longum* R0175 significantly reduced bowel movement frequency in patients with IBS-D compared to placebo (Efficacy level B, Table 3). The top three treatments based on SUCRAs were EF + LA + X (89.7%), *Lactobacillus paracasei* B21060 (89.2%), and *Lactobacillus paracasei* HA-196 (78.7%). 

The IBS-C evidence network was constructed using five single-strain probiotics and two multistrain groups from eight RCTs (585 patients, Figure 3). However, no significant difference was found between the pooled probiotic and placebo groups (Table 3).

#### 3.3.7. Bristol Stool form Scale in IBS-D and IBS-C

The IBS-D evidence network was constructed with seven single-strain probiotics and five multistrain groups from 12 RCTs (833 patients, Figure 3). NMA indicated that *Bacillus coagulans* MTCC 5856 (MD, −3.28; 95% CI, −5.21 to −1.34) and *Saccharomyces cerevisiae* CNCM I-3856 (MD, −1.24; 95% CI, −1.63 to −0.86) were classified as efficacy level A (Table 3). NMA indicated that BL + LR, LA + LR + ST + BL + BBR + X, and LP + LP + PA were significantly superior to placebo in improving the Bristol stool form score (efficacy level B, Table 3). The top three treatments based on SUCRAs were *Bacillus coagulans* MTCC 5856 (99.6%), *Saccharomyces cerevisiae* CNCM I-3856 (89.7%), and BL + LR (75%).

The IBS-C evidence network was constructed with three single-strain probiotics and one multistrain group from four RCTs (161 patients, Figure 3). No significant difference was found between pooled probiotics and placebo (Table 3).

### 3.4. Adverse Events

Most studies did not assess causality between intervention and adverse events. In some studies, abdominal symptoms (pain, bloating, or diarrhea) were recorded as adverse events. But there was no identification of whether these symptoms were attributed to a flare-up of IBS. Hence, there may be inherent heterogeneity among data on adverse events, and we did not conduct a meta-analysis. The overall rate of adverse events was 11.62% (452/3891) in the probiotic group and 10.61% (379/3572) in the placebo group. The rate of serious adverse events was 0.15% (6/4081) in the probiotics group and 0.22% (8/3679) in the placebo group.

### 3.5. Heterogeneity and Inconsistency

Prediction interval plots are presented in Figure S5. No significant inconsistency between direct and indirect evidence was observed in the abdominal pain score, abdominal bloating score, or bowel movement frequency in IBS-D. The treatment loops of five outcomes (IBS-SSS, HADS-total score, abdominal pain score, abdominal bloating score, Bowel movement frequency, and Bristol stool form scale) were formed only by triple-arm trials; therefore, the NMA was consistent by definition. Inconsistency could not be assessed in the remaining five outcomes because there were no loops in their evidence networks (Table S6). Funnel plots are shown in Figure S6.

### 3.6. Quality of Evidence

The quality of efficacy rankings was high in one outcome, moderate in eight outcomes, and low in two outcomes (Table S7).

### 3.7. Alteration of Gut Microbiota

The alterations of bacterial abundance and diversity in the probiotic group are presented in Tables S8 and S9. Six studies calculated the gut microbiota diversity before and after the intervention. In five studies, the differences between probiotics and placebo in modulating alpha diversity were reported as not being significant. Regarding beta diversity, three studies revealed significant differences between the two groups.

## 4. Discussion

### 4.1. Key Findings

In this systematic review and network meta-analysis, we compared and ranked the efficacy of different probiotic strains or mixtures based on symptom-specific outcomes. We found that only some probiotic strains and combinations were more effective than the placebo for each specific outcome of IBS. This NMA provides an initial indication of promising probiotic strains or mixtures that can be used in the treatment of IBS patients.

For the global condition and mental health stage of IBS, moderate-certainty evidence indicates that *Lactobacillus acidophilus* DDS-1, BL + LR, LC + LP + BAL, LP + LC + BL + ST + LA + X, and *Bifidobacterium animalis* subsp. *lactis* UABla-12 are among the most effective probiotics for improving IBS-SSS. The mixture (LP + LC + BL + ST + LA + X) was the most effective probiotic for improving IBS-QOL (moderate certainty). High-certainty evidence indicates that *Bifidobacterium longum* R0175 and *Bifidobacterium longum* NCC3001 are the most effective probiotics for improving HADS-total and HADS-depression scores, respectively. 

In terms of the core and specific gastrointestinal symptoms of IBS, moderate-certainty evidence indicates that *Bacillus coagulans* MTCC 5856 and *Bacillus coagulans* Unique IS2 are among the most effective probiotics for improving abdominal pain. BL + LR, *Lactobacillus plantarum* CCFM8610, *Lactobacillus plantarum* 299v, VSL#3, *Bifidobacterium bifidum* MIMBb75 were significantly superior to placebo in alleviating abdominal bloating. EF + LA + X was most effective in reducing bowel movement frequency in patients with IBS-D (moderate certainty). *Bacillus coagulans* MTCC 5856 and *Saccharomyces cerevisiae* CNCM I-3856 were among the most effective probiotics for improving the Bristol stool form scale in IBS-D. No significant difference was found among the pooled probiotics and placebo regarding bowel movement frequency and Bristol stool form scale in the IBS-C group.

### 4.2. Associations with Current Studies

The three current guidelines (BSG, ACG, and The American Gastroenterological Association) still have reservations about the use of probiotics in IBS due to the concern of between-study heterogeneity [12,18,19]. The main challenges in interpreting the existing evidence regarding probiotics in IBS treatments were the numerous strains and combinations of probiotics, different doses, the lack of standard outcomes, and the inconsistency of results [18]. In terms of probiotic taxonomy, the ideal NMA of probiotics in IBS treatment should be analyzed based on the strain level [19,117]. McFarland et al. conducted the first meta-analysis to evaluate the strain- and outcome-specific efficacy of probiotics for IBS [118]. This study included only 14 probiotic formulas because the inclusion criteria required at least two trials within each type of probiotic. The comparative efficacy of different probiotics was also lacking in McFarland’s work owing to the limitations of a traditional pairwise meta-analysis. Although some probiotics were confirmed by a single RCT, we advocate that clinical trials with large sample sizes and high quality should not be excluded from related meta-analyses. Additionally, the efficacy hierarchy is beneficial for probiotic selection in clinical practice. Given the numerous types of probiotics, NMA is ideal for comparing the efficacy.

A standard system for efficacy evaluation is needed in IBS clinical trials. The system should be composed of endpoints that can precisely reflect the change in the core signs and symptoms of IBS. In this NMA, we chose the abdominal pain score, bowel movement frequency (per week), and Bristol stool form scale to assess the most important symptoms of IBS in accordance with the diagnostic elements in ROME IV and the guidance of the US Food and Drug Administration [119,120]. The global condition of IBS was evaluated by the IBS-SSS and IBS-QOL. The IBS-SSS is widely used in IBS symptom severity assessment [121]. IBS-QOL is a valid scoring system for assessing the physical and mental condition of IBS patients [122]. The evaluation of psychological conditions is indispensable for IBS assessments. An increasing number of psychological problems have been associated with worse gastrointestinal symptoms in IBS [123]. Hence, the HADS was also included in this NMA. The above seven outcomes present a panorama of efficacy evaluation in IBS treatment, including disease-defined core symptoms, global conditions, psychological conditions, and subtype-specific symptoms. The binary outcomes of IBS were not included in this NMA because related RCTs used various definitions of “improvement/response rate”, which incur potential heterogeneity.

ROME IV indicates that identifying the main and/or most troubling symptoms is the first step in the treatment of patients with IBS [119]. This was reflected in the different pharmacological treatments of the guidelines, such as antispasmodics, guanylate cyclase-C agonists, and antidepressants [12,124,125]. These drugs have definite pharmacological effects targeting particular IBS symptoms. However, the effects of probiotics on specific symptoms of IBS are still unclear. Ford et al. conducted a comprehensive and rigorous meta-analysis to evaluate the efficacy of probiotics in IBS patients. The results indicated that combinations of probiotics were associated with significant improvements in IBS symptom and flatulence scores as well as a trend of decreasing bloating scores. However, these benefits were not observed when specific combinations or strains were analyzed. The results supported the use of combinations of probiotics as a group [20]. In other words, the use of probiotic combinations may be beneficial from the perspective of the entire IBS population. On the other hand, identifying the comparative efficacy of specific probiotics was also significant for individual patients. Zhang et al. performed a large NMA that calculated the relative ranking of 12 different probiotics on 7 outcomes. The results showed that, based on SUCRA analysis, *Bacillus coagulans* was ranked first in improving the symptom relief rate, global symptoms, abdominal pain, bloating, and straining [126]. Our NMA yielded results similar to those of the above two studies. *Lactobacillus acidophilus* DDS-1, *Bifidobacterium animalis* subsp. *lactis* UABla-12, and the three probiotic mixtures may improve the global condition of IBS. The two strains of *Bacillus coagulans* may improve the abdominal pain score of patients with IBS. We used a novel and succinct approach to classify efficacy. Seven probiotic strains and five mixtures were evaluated as level A (among the most effective) for different outcomes. A list of probiotics that may be effective for each outcome is also provided. Different probiotics should be selected according to the specific symptoms of IBS patients.

### 4.3. Study Merits and Limitations

A plethora of meta-analyses have been published on this topic, but our NMA has several merits. To our knowledge, this study is the first NMA to compare the outcome-specific and strain-level efficacies of different probiotics in IBS. To date, it is the most comprehensive systematic review of the probiotic efficacy in IBS. We included 81 RCTs and 9253 patients, which was attributed to a rigorous literature search and the inclusion of probiotic combinations. This NMA used seven outcomes to conduct full-scale evaluations of probiotics in IBS treatments, including disease-defined core symptoms, global conditions, psychological conditions, and subtype-specific symptoms. 

Our study had several limitations. First, there was an inherent heterogeneity among the included RCTs. The study regions, diagnostic criteria, treatment durations, and probiotic doses varied in the pooled studies, undermining the reliability of the results. The classification of probiotic combinations was partly based on the strain level, which resulted in inherent heterogeneity. A more rational classification of probiotic combinations in NMA with less heterogeneity will enhance the quality of evidence in this field. In future studies, the above factors should be considered in NMA, which will help evaluate the efficacy of probiotics in the treatment of IBS. Second, the robustness and complexity of the evidence network are unsatisfactory. The comparative efficacy and ranking of some probiotics, especially single-strain products, are often based on single studies. The scarcity of RCTs has a negative impact on the certainty of the NMA results. On the other hand, the evidence network of the outcomes was “star-shaped”. Most probiotics have been directly compared with placebo. Only three three-arm RCTs provided direct comparisons between different probiotics. This prevents the use of NMA for evaluating the efficacy of different probiotics by combining direct and indirect comparisons. Third, the evaluation of long-term efficacy was not available because few studies reported long-term results with more than one year of follow-up.

The results of this study only reflect the relative efficacy of probiotics assessed by meta-analyses based on the available studies. Further studies are required to confirm the efficacy of probiotics in the real world. A standard evaluation system of efficacy, safety, and gut microbiota alteration is needed in future clinical trials of IBS. Multicenter RCTs are necessary to evaluate the efficacy of strain-specific probiotics in IBS treatment.

## 5. Conclusions

In conclusion, this NMA provides the first efficacy ranking of different probiotic strains and combinations for specific IBS outcomes. *Lactobacillus acidophilus* DDS-1, *Bifidobacterium animalis* subsp. *Lactis* UABla-12, *Bifidobacterium longum* R0175, *Bifidobacterium longum* NCC3001, *Bacillus coagulans* MTCC 5856, *Bacillus coagulans* Unique IS2, *Saccharomyces cerevisiae* CNCM I-3856, and four mixtures may be the most promising probiotics. Probiotics should be selected according to the specific symptoms of IBS patients. Due to the inherent heterogeneity, this evidence should be interpreted with caution. Further studies are needed to confirm these results.

## Figures and Tables

**Table 1:** Critical results of network meta-analysis for the global condition and mental health condition in patients with IBS.

Outcome	Efficacy Level A ^1^			Efficacy Level B			Efficacy Level C
	Probiotic	NMA ^2^	GRADE	Probiotic	NMA	GRADE	Probiotic
IBS-SSS	*L. acidophilus* DDS-1	−77.70 (−101.72, −53.68)	M	EF + LA + X	−35.00 (−60.44, −9.56)	M	*L. acidophilus* ATCC 700396, *L. plantarum* 299v, *L. plantarum* CCFM8610, *L. casei* LCR35, *L. paracasei* HA-196, *B. longum* R0175, BAL + ST + LB, LR + PF + X
	BL + LR	−80.99 (−130.73, −31.26)	M	*B. bifidum* MIMBb75	−29.83 (−48.24, −11.42)	H	
	LC + LP + BAL	−76.42 (−114.90, −37.95)	M	*C. butyricum* CGMCC0313.1	−21.38 (−40.47, −2.29)	H	
	LP + LC + BL + ST + LA + X	−63.96 (−78.66, −49.26)	M	BAL + LA + BB + X	−18.86 (−25.88, −11.85)	M	
	*B. lactis* UABla-12	−48.80 (−73.00, −24.60)	M				
IBS-QOL	LP + LC + BL + ST + LA + X	24.80 (20.65, 28.95)	M	*C. butyricum* CGMCC0313.1	4.07 (0.50, 7.65)	H	LP + LC + BL + ST + LA + X, *S. boulardii* CNCM I-475, LC + LP + BAL, LA + LR + ST + BL + BBR + X, EF + LA + X, BAL + ST + LB, LPA + LA + BAL, *L. acidophilus* ATCC 700396
HADS-total score	*B. longum* R0175	−0.34 (−0.48, −0.20)	H	None			*L. paracasei* HA-196, *L. acidophilus* ATCC 700396
HADS-anxiety	None			None			*B. longum* NCC3001, *L. acidophilus* ATCC 700396, LPA + LA + BAL
HADS-depression	*B. longum* NCC3001	−3.00 (−4.92, −1.08)	H	None			*L. paracasei* CNCM I-1572, *L. acidophilus* ATCC 700396, LPA + LA + BAL

^1^ Efficacy level A (among the most effective): probiotics that are significantly superior to placebo and at least 1 probiotic in level B. Efficacy level B: probiotics that are more effective than placebo, but not superior to any other of the probiotic(s) superior to placebo. Efficacy level C (among the least effective): probiotics with no significant difference compared to placebo. ^2^ The network meta-analysis results were the efficacy of probiotics compared to placebo. Mean difference with 95% confidence intervals in parentheses. Abbreviations: IBS-SSS, IBS Symptom Severity Scale; IBS-QOL, IBS-Quality of Life Measure; HADS, the Hospital Anxiety and Depression Scale; GRADE, The Grading of Recommendations, Assessment, Development and Evaluation system; H, high; M, moderate; L, low.

**Table 2:** Critical results of network meta-analysis for abdominal pain and bloating in patients with IBS.

Outcome	Efficacy Level A ^1^			Efficacy Level B			Efficacy Level C
	Probiotic	NMA ^2^	GRADE	Probiotic	NMA	GRADE	Probiotic
Abdominal pain score	*B. coagulans* MTCC 5856	−41.80 (−61.59, −22.00)	M	*L. gasseri* BNR17	−36.10 (−64.53, −7.67)	M	BL + LR, *L. acidophilus* SDC 2012,2013, *B. lactis* UABla-12, BAL + LA + BB + X, LR + PF + X, LA + LR + ST + BL + BBR + X, *B. bifidum* MIMBb75, LP + LP + PA, LP + LC + BL + ST + LA + X, LA + LA, *C. butyricum* CGMCC0313.1, *L. plantarum* 299v, *L. acidophilus* ATCC 700396, *L. casei* LCR35, BAL + ST + LB, LPA + LA + BAL, BAL + LA + LR + X, *S. boulardii* CNCM I-475, *B. infantis* 35624, *L. plantarum* CCFM8610
	*B. coagulans* Unique IS2	−32.00 (−45.35, −18.65)	M	*L. plantarum* Apsulloc 331261	−26.59 (−47.07, −6.11)	M	
				*L. acidophilus* DDS-1	−19.53 (−33.49, −5.57)	M	
				LPA + LS + LP	−20.00 (−35.97, −4.03)	L	
				*S. cerevisiae* CNCM I-3856	−15.24 (−24.62, −5.87)	M	
				VSL#3	−12.93 (−25.59, −0.26)	L	
				EF + LA + X	−11.37 (−21.68, −1.06)	M	
				LA + ST + X	−8.14 (−15.54, −0.75)	M	
Abdominal bloating score	None			BL + LR	−34.00 (−56.94, −11.06)	M	*B. coagulans* MTCC 5856, *L. plantarum* Apsulloc 331261, *L. acidophilus* DDS-1, LR + PF + X, LA + ST + X, *B. coagulans* Unique IS2, *S. cerevisiae* CNCM I-3856, BAL + LA + BB + X, *B. lactis* UABla-12, LP + LC + BL + ST + LA + X, EF + LA + X, LA + LR + ST + BL + BBR + X, *L. acidophilus* ATCC 700396, BAL + LA + LR + X, *S. boulardii* CNCM I-475, *C. butyricum* CGMCC0313.1, BAL + ST + LB, *B. infantis* 35624, LPA + LA + BAL
				*L. plantarum* CCFM8610	−19.92 (−34.91, −4.94)	M	
				*L. plantarum* 299v	−14.79 (−29.11, −0.48)	M	
				VSL#3	−13.71 (−22.12, −5.30)	L	
				*B. bifidum* MIMBb75	−11.83 (−22.93, −0.74)	M	

^1^ Efficacy level A (among the most effective): probiotics that are significantly superior to placebo and at least 1 probiotic in level B. Efficacy level B: probiotics that are more effective than placebo, but not superior to any other of the probiotic(s) superior to placebo. Efficacy level C (among the least effective): probiotics with no significant difference compared to placebo. ^2^ The network meta-analysis results were the efficacy of probiotics compared to placebo. Mean difference with 95% confidence intervals in parentheses. Abbreviations: GRADE, The Grading of Recommendations, Assessment, Development and Evaluation system; H, high; M, moderate; L, low.

**Table 3:** Critical results of network meta-analysis for bowel movement frequency and Bristol stool form scale in patients with IBS.

Outcome	Efficacy Level A ^1^			Efficacy Level B			Efficacy Level C
	Probiotic	Network Meta-Analysis ^2^	GRADE	Probiotic	Network Meta-Analysis	GRADE	Probiotic
Bowel movement frequency (IBS-D)	EF + LA + X	−3.95 (−5.02, −2.88)	M	*L. paracasei* B21060	−5.11 (−9.98, −0.24)	H	*L. plantarum* Apsulloc 331261, LP + LC + BL + ST + LA + X, *C. butyricum* CGMCC0313.1, *S. cerevisiae* CNCM I-3856, *S. boulardii* CNCM I-475
				*L. paracasei* HA-196	−3.13 (−4.63, −1.62)	H	
				*B. coagulans* GBI-306086	−2.11 (−3.00, −1.23)	M	
				*B. longum* R0175	−1.95 (−3.45, −0.45)	H	
Bowel movement frequency (IBS-C)	None			None			*B. bifidum* MIMBb75, LA + ST + X, *S. cerevisiae* CNCM I-3856, *L. paracasei* HA-196, *B. coagulans* Unique IS2, *B. longum* R0175, BAL + ST + LB
Bristol stool form scale (IBS-D)	*B. coagulans* MTCC 5856	−3.28 (−5.21, −1.34)	L	BL + LR	−0.80 (−1.57, −0.03)	L	*L. paracasei* HA-196, *B. bifidum* MIMBb75, VSL#3, *C. butyricum* CGMCC0313.1, *B. longum* R0175, LP + LC + BL + ST + LA + X, *S. boulardii* CNCM I-475
	*S. cerevisiae* CNCM I-3856	−1.24 (−1.63, −0.86)	L	LA + LR + ST + BL + BBR + X	−0.70 (−1.32, −0.08)	M	
				LP + LP + PA	−0.50 (−0.76, −0.24)	L	
Bristol stool form scale (IBS-C)	None			None			*L. paracasei* HA-196, *S. cerevisiae* CNCM I-3856, *B. longum* R0175, BAL + ST + LB

^1^ Efficacy level A (among the most effective): probiotics that are significantly superior to placebo and at least 1 probiotic in level B. Efficacy level B: probiotics that are more effective than placebo, but not superior to any other of the probiotic(s) superior to placebo. Efficacy level C (among the least effective): probiotics with no significant difference compared to placebo. ^2^ The network meta-analysis results were the efficacy of probiotics compared to placebo. Mean difference with 95% confidence intervals in parentheses. Abbreviations: IBS-D, IBS with predominant diarrhea; IBS-C, IBS with predominant constipation; GRADE, The Grading of Recommendations, Assessment, Development and Evaluation system; H, high; M, moderate; L, low.

## Data Availability

Not applicable.

## Supplementary Materials

The following supporting information can be downloaded at: https://www.mdpi.com/article/10.3390/nu15173856/s1. Text S1. Supplements and alterations of the original protocol; Text S2. Search strategy; Text S3. Data collection process; Text S4. References to RCTs included in this network meta-analysis; Figure S1. The distribution of effect modifiers; Figure S2. Risk of bias 2 evaluations for each outcome; Figure S3. Network plots; Figure S4. Cumulative ranking curves for each outcome; Figure S5. Predictive interval plots for each pair of interventions; Figure S6. Funnel plots for each outcome; Table S1. Characteristics of included randomized controlled trials (1); Table S2. Characteristics of included randomized controlled trials (2); Table S3. Critical results of pairwise meta-analysis; Table S4. League table demonstrating the relative efficacy for each pair of comparison in the NMA; Table S5. The surface under the cumulative ranking curve (SUCRA) values; Table S6. Results of loop specific analysis, node-splitting method, and design-by-treatment interaction inconsistency model; Table S7. GRADE-based assessment of the quality of evidence for each outcome; Table S8. Alterations of gut microbiota abundance; Table S9. The alteration of gut microbiota diversity before and after intervention.
