# Supplementary material for: Outcome-Specific Efficacy of Different Probiotic Strains and Mixtures in Irritable Bowel Syndrome: A Systematic Review and Network Meta-Analysis
Source: Nutrients. 2023 Sep 4;15(17):3856. doi: 10.3390/nu15173856 (PMC10490209; doi:10.3390/nu15173856)
Supplement: Supplementary file 1 [file nutrients-15-03856-s001.zip › nutrients-2586871-supplementary.pdf]

Supplementary Materials

Supplement to:

Outcome-Specific Efficacy of Different Probiotic Strains and Mixtures in Irritable Bowel Syndrome: A Systematic Review and Network Meta-Analysis

Appendix

Text S1. Supplements and alterations of the original protocol 2

Text S2. Search strategy 3

Text S3. Data collection process 5

Text S4. References to RCTs included in this network meta-analysis 6

Figure S1. The distribution of effect modifiers 10

Figure S2. Risk of bias 2 evaluations for each outcome 13

Figure S3. Network plots 17

Figure S4. Cumulative ranking curves for each outcome 22

Figure S5. Predictive interval plots for each pair of interventions 27

Figure S6. Funnel plots for each outcome 33

Table S1. Characteristics of included randomized controlled trials (1) 37

Table S2. Characteristics of included randomized controlled trials (2) 40

Table S3. Critical results of pairwise meta-analysis 43

Table S4. League table demonstrating the relative efficacy for each pair of comparison in the NMA 49

Table S5. The surface under the cumulative ranking curve (SUCRA) values 55

Table S6. Results of loop specific analysis, node-splitting method and design-by-treatment interaction inconsistency model 60

Table S7. GRADE-based assessment of the quality of evidence for each outcome 61

Table S8. Alterations of gut microbiota abundance 65

Table S9. The alteration of gut microbiota diversity before and after intervention 66

Text S1. Supplements and alterations of the original protocol.

In order to make our NMA more comprehensive and rigorous, we have made the following additions and alterations based on the original protocol.

1. Literatures were searched from inception until June 1, 2023.
2. Outcomes regarding the change in the Hospital Anxiety and Depression Scale, bowel movement frequency (per week), Bristol stool form scale score were added to this NMA.
3. Data regarding the adverse events for each study were collected.

Text S2. Search Strategy

1. Web of science

#1	(TS=(irritable bowel syndrome)) OR TS=(IBS) and Preprint Citation Index (Exclude – Database)
#2	(((TS=(clinical trial)) OR TS=(clinical trials)) OR TS=(random*)) OR TS=(random allocation) and Preprint Citation Index (Exclude – Database)
#3	(((((((TS=(Probiotic*)) OR TS=(Lactobacillus)) OR TS=(Saccharomyces)) OR TS=(Bacillus)) OR TS=(Bifidobacterium)) OR TS=(Clostridium)) OR TS=(Streptococcus)) OR TS=(Enterococcus) and Preprint Citation Index (Exclude – Database)
#4	#3 AND #2 AND #1 and Preprint Citation Index (Exclude – Database)
#5	#4 and Preprint Citation Index (Exclude – Database) Timespan: 1950-01-01 to 2023-06-01 (Publication Date)

2. Pubmed

#1	Search: ((irritable bowel syndrome[Title/Abstract]) OR (IBS[Title/Abstract])) OR (irritable bowel syndrome[MeSH Terms]) Sort by: Most Recent
#2	Search: ((((((((Probiotic*[Title/Abstract]) OR (Probiotic[MeSH Terms])) OR (Lactobacillus[Title/Abstract])) OR (Saccharomyces[Title/Abstract])) OR (Bacillus[Title/Abstract])) OR (Bifidobacterium[Title/Abstract])) OR (Clostridium[Title/Abstract])) OR (Streptococcus[Title/Abstract])) OR (Enterococcus[Title/Abstract]) Sort by: Most Recent
#3	Search: (((((clinical trial[Title/Abstract]) OR (clinical trials[MeSH Terms])) OR (clinical trial[Publication Type])) OR (random*[Title/Abstract])) OR (random allocation[MeSH Terms])) OR (therapeutic use[MeSH Subheading]) Sort by: Most Recent
#4	Search: ((((irritable bowel syndrome[Title/Abstract]) OR (IBS[Title/Abstract])) OR (irritable bowel syndrome[MeSH Terms])) AND (((((((((Probiotic*[Title/Abstract]) OR (Probiotic[MeSH Terms])) OR (Lactobacillus[Title/Abstract])) OR (Saccharomyces[Title/Abstract])) OR (Bacillus[Title/Abstract])) OR (Bifidobacterium[Title/Abstract])) OR (Clostridium[Title/Abstract])) OR (Streptococcus[Title/Abstract])) OR (Enterococcus[Title/Abstract]))) AND ((((((clinical trial[Title/Abstract]) OR (clinical trials[MeSH Terms])) OR (clinical trial[Publication Type])) OR (random*[Title/Abstract])) OR (random allocation[MeSH Terms])) OR (therapeutic use[MeSH Subheading])) Sort by: Most Recent
#5	Search: ((((irritable bowel syndrome[Title/Abstract]) OR (IBS[Title/Abstract])) OR (irritable bowel syndrome[MeSH Terms])) AND (((((((((Probiotic*[Title/Abstract]) OR (Probiotic[MeSH Terms])) OR (Lactobacillus[Title/Abstract])) OR (Saccharomyces[Title/Abstract])) OR (Bacillus[Title/Abstract])) OR (Bifidobacterium[Title/Abstract])) OR (Clostridium[Title/Abstract])) OR (Streptococcus[Title/Abstract])) OR (Enterococcus[Title/Abstract]))) AND ((((((clinical trial[Title/Abstract]) OR (clinical trials[MeSH Terms])) OR (clinical trial[Publication Type])) OR (random*[Title/Abstract])) OR (random allocation[MeSH Terms])) OR (therapeutic use[MeSH Subheading])) Filters: from 1950/1/1 - 2023/6/1 Sort by: Most Recent

3. Cochrane Central Register of Controlled Trials

#1	MeSH descriptor: [Irritable Bowel Syndrome] explode all trees
#2	(irritable bowel syndrome):ti,ab,kw OR (IBS):ti,ab,kw
#3	(probiotic):ti,ab,kw OR (probiotics):ti,ab,kw
#4	(Lactobacillus):ti,ab,kw OR (Saccharomyces):ti,ab,kw OR (Bacillus):ti,ab,kw OR (Bifidobacterium):ti,ab,kw OR (Clostridium):ti,ab,kw
#5	(Streptococcus):ti,ab,kw OR (Enterococcus):ti,ab,kw
#6	#1 OR #2
#7	#3 OR #4 OR #5
#8	#6 AND #7 with Cochrane Library publication date from Jan 1950 to Jun 2023

4. ClinicalTrials.gov

Condition or disease:	irritable bowel syndrome
Study type:	Interventional Studies (Clinical Trials)
Age	Adult (18 - 64) OR Older adult (65+)
Date Range (Primary Completion):	From 01/01/1950 To 06/01/2023
All the rest options were default	

208

5. Embase

Sources：Embase, MEDLINE and Preprints

#1	'irritable bowel syndrome':ti,ab,kw OR 'ibs':ti,ab,kw OR 'irritable bowel syndrome'/exp OR 'ibs' OR 'irritable colon'/exp
#2	'probiotic':ti,ab,kw OR 'lactobacillus':ti,ab,kw OR 'saccharomyces':ti,ab,kw OR 'bacillus':ti,ab,kw OR 'bifidobacterium':ti,ab,kw OR 'clostridium':ti,ab,kw OR 'streptococcus':ti,ab,kw OR 'enterococcus':ti,ab,kw OR 'probiotic agent'/exp OR 'probiotics':ti,ab,kw
#3	'clinical trial':ti,ab,kw OR 'clinical trials':ti,ab,kw OR 'random*':ti,ab,kw OR 'clinical trial'/exp
#4	#1 AND #2 AND #3
#5	#4 AND 'randomized controlled trial'/de

Text S3. Data collection process

Two authors (PW Xie and M Luo) independently screened titles and abstracts of the search results. The full-text of remaining articles will be examined based on the defined eligibility criteria. The senior investigator (LS Xiong) conducted a discussion when disagreements occurred. Disagreements will be resolved by consensus. Two authors (XH Deng and JH Fan) used a piloted electronic form to extract data independently. The form was based on the Cochrane Collaboration's Data Collection Form Template. Data extraction includes the following items: RCT information (article title, first author, publication year, trial location, trial design, inclusion and exclusion criteria, composition and dose of probiotics, follow-up period), population characteristics (IBS diagnosis criteria, IBS duration and severity, sample size, gender, mean age or age range, BMI), outcomes of interest. For RCTs reported at different follow-up time, all relevant articles were included in the same group for data extraction. Corresponding authors were requested for original data by e-mail if the data of outcomes did not report in the full-text. Intention-to-treat analyses were used in data collection.

Text S4. References to RCTs included in this network meta-analysis

1. Mourey F, Decherf A, Jeanne J-F, et al. Saccharomyces cerevisiae I-3856 in irritable bowel syndrome with predominant constipation. *World J Gastroenterol* 2022; 28(22): 2509-22.
2. Jung K, Kim A, Lee J-H, et al. Effect of Oral Intake of Lactiplantibacillus plantarum APsulloc 331261 (GTB1 (TM)) on Diarrhea-Predominant Irritable Bowel Syndrome: A Randomized, Double-Blind, Placebo-Controlled Study. *Nutrients* 2022; 14(10).
3. Bai T, Xu Z, Xia P, et al. The Short-Term Efficacy of Bifidobacterium Quadruple Viable Tablet in Patients With Diarrhea-Predominant Irritable Bowel Syndrome: Potentially Mediated by Metabolism Rather Than Diversity Regulation. *Am J Gastroenterol.* 2022: 10.14309/ajg.0000000000002147.
4. Xu H, Ma C, Zhao F, et al. Adjunctive treatment with probiotics partially alleviates symptoms and reduces inflammation in patients with irritable bowel syndrome. *Eur J Nutr.* 2021; 60(5): 2553-65.
5. Skrzydlo-Radomanska B, Prozorow-Krol B, Cichoz-Lach H, et al. The Effectiveness and Safety of Multi-Strain Probiotic Preparation in Patients with Diarrhea-Predominant Irritable Bowel Syndrome: A Randomized Controlled Study. *Nutrients* 2021; 13(3).
6. Liu Y, Yu X, Yu L, et al. Lactobacillus plantarum CCFM8610 Alleviates Irritable Bowel Syndrome and Prevents Gut Microbiota Dysbiosis: A Randomized, Double-Blind, Placebo-Controlled, Pilot Clinical Trial. *Engineering* 2021; 7(3): 376-85.
7. Gupta AK, Maity C. Efficacy and safety of Bacillus coagulans LBSC in irritable bowel syndrome A prospective, interventional, randomized, double-blind, placebo-controlled clinical study CONSORT Compliant. *Medicine* 2021; 100(3).
8. Barraza-Ortiz DA, Perez-Lopez N, Medina-Lopez VM, et al. Combination of a Probiotic and an Antispasmodic Increases Quality of Life and Reduces Symptoms in Patients with Irritable Bowel Syndrome: A Pilot Study. *Dig Dis.* 2021; 39(3): 294-300.
9. Sadrin S, Sennoune S, Gout B, et al. A 2-strain mixture of Lactobacillus acidophilus in the treatment of irritable bowel syndrome: A placebo-controlled randomized clinical trial. *Dig Liver Dis.* 2020; 52(5): 534-40.
10. Martoni CJ, Srivastava S, Leyer GJ. Lactobacillus acidophilus DDS-1 and Bifidobacterium lactis UABla-12 Improve Abdominal Pain Severity and Symptomology in Irritable Bowel Syndrome: Randomized Controlled Trial. *Nutrients* 2020; 12(2).
11. Lewis ED, Antony JM, Crowley DC, et al. Efficacy of Lactobacillus paracasei HA-196 and Bifidobacterium longum R0175 in Alleviating Symptoms of Irritable Bowel Syndrome (IBS): A Randomized, Placebo-Controlled Study. *Nutrients* 2020; 12(4).
12. Kim J, Cho K, Kim JS, et al. Probiotic treatment induced change of inflammation related metabolites in IBS-D patients/double-blind, randomized, placebo-controlled trial. *Food Sci Biotechnol.* 2020; 29(6): 837-44.
13. Gayathri R, Aruna T, Malar S, Shilpa B, Dhanasekar KR. Efficacy of Saccharomyces cerevisiae CNCM I-3856 as an add-on therapy for irritable bowel syndrome. *Int J Colorectal Dis.* 2020; 35(1): 139-45.
14. Bonfrate L, Di Palo DM, Celano G, et al. Effects of Bifidobacterium longum BB536 and Lactobacillus rhamnosus HN001 in IBS patients. *Eur J Clin Invest.* 2020; 50(3).
15. Andresen V, Gschossmann J, Layer P. Heat-inactivated Bifidobacterium bifidum MIMBb75 (SYN-HI-001) in the treatment of irritable bowel syndrome: a multicentre, randomised, double-blind, placebo-controlled clinical trial. *Lancet Gastroenterol Hepatol.* 2020; 5(7): 658-66.
16. Oh JH, Jang YS, Kang D, Chang DK, Min YW. Efficacy and Safety of New Lactobacilli Probiotics for Unconstipated Irritable Bowel Syndrome: A Randomized, Double-Blind, Placebo-Controlled Trial. *Nutrients* 2019; 11(12).
17. Madempudi RS, Ahire JJ, Neelamraju J, Tripathi A, Nanal S. Randomized clinical trial: the effect of probiotic Bacillus coagulans Unique IS2 vs. placebo on the symptoms management of irritable bowel syndrome in adults. *Sci Rep.* 2019; 9.
18. Ashtiani SY, Amery M. Effect of Multispecies Probiotic Supplementation on Irritable Bowel Syndrome. *J Pharm Res Int.* 2019; 28(6).
19. Sun Y-Y, Li M, Li Y-Y, et al. The effect of Clostridium butyricum on symptoms and fecal microbiota in diarrhea-dominant irritable bowel syndrome: a randomized, double-blind, placebo-controlled trial. *Sci Rep.* 2018; 8.
20. Shin SP, Choi YM, Kim WH, et al. A double blind, placebo-controlled, randomized clinical trial that breast milk derived Lactobacillus gasseri BNR17 mitigated diarrhea-dominant irritable bowel syndrome. *J Clin Biochem Nutr.* 2018; 62(2): 179-

86.
21. Preston K, Krumian R, Hattner J, de Montigny D, Stewart M, Gaddam S. Lactobacillus acidophilus CL1285, Lactobacillus casei LBC80R and Lactobacillus rhamnosus CLR2 improve quality-of-life and IBS symptoms: a double-blind, randomised, placebo-controlled study. *Benefic Microbes.* 2018; 9(5): 697-706.
22. Kim JY, Park YJ, Lee HJ, Park MY, Kwon O. Effect of Lactobacillus gasseri BNR17 on irritable bowel syndrome: a randomized, double-blind, placebo-controlled, dose-finding trial. *Food Sci Biotechnol.* 2018; 27(3): 853-7.
23. Ishaque SM, Khosruzzaman SM, Ahmed DS, Sah MP. A randomized placebo-controlled clinical trial of a multi-strain probiotic formulation (Bio-Kult (R)) in the management of diarrhea- predominant irritable bowel syndrome. *BMC Gastroenterol.*2018; 18.
24. Cremon C, Guglielmetti S, Gargari G, et al. Effect of Lactobacillus paracasei CNCM I-1572 on symptoms, gut microbiota, short chain fatty acids, and immune activation in patients with irritable bowel syndrome: A pilot randomized clinical trial. *United Eur Gastroenterol J.* 2018; 6(4): 604-13.
25. Pinto-Sanchez MI, Hall GB, Ghajar K, et al. Probiotic Bifidobacterium longum NCC3001 Reduces Depression Scores and Alters Brain Activity: A Pilot Study in Patients With Irritable Bowel Syndrome. *Gastroenterology* 2017; 153(2): 448-+.
26. Nobutani K, Sawada D, Fujiwara S, et al. The effects of administration of the Lactobacillus gasseri strain CP2305 on quality of life, clinical symptoms and changes in gene expression in patients with irritable bowel syndrome. *J Appl Microbiol.* 2017; 122(1): 212-24.
27. Hod K, Sperber AD, Ron Y, et al. A double-blind, placebo-controlled study to assess the effect of a probiotic mixture on symptoms and inflammatory markers in women with diarrhea-predominant IBS. *Neurogastroenterol Motil.* 2017; 29(7).
28. Thijssen AY, Clemens CHM, Vankerckhoven V, Goossens H, Jonkers DMAE, Masclee AAM. Efficacy of Lactobacillus casei Shirota for patients with irritable bowel syndrome. *Eur J Gastroenterol Hepatol.* 2016; 28(1): 8-14.
29. Spiller R, Pelerin F, Decherf AC, et al. Randomized double blind placebo-controlled trial of Saccharomyces cerevisiae CNCM I-3856 in irritable bowel syndrome: improvement in abdominal pain and bloating in those with predominant constipation. *United Eur Gastroenterol J.* 2016; 4(3): 353-62.
30. 68 Mezzasalma V, Manfrini E, Ferri E, et al. A Randomized, Double-Blind, Placebo-Controlled Trial: The Efficacy of Multispecies Probiotic Supplementation in Alleviating Symptoms of Irritable Bowel Syndrome Associated with Constipation. *Biomed Research International* 2016; 2016.
31. Majeed M, Nagabhushanam K, Natarajan S, et al. Bacillus coagulans MTCC 5856 supplementation in the management of diarrhea predominant Irritable Bowel Syndrome: a double blind randomized placebo controlled pilot clinical study. *Nutr J.* 2016; 15.
32. Lyra A, Hillila M, Huttunen T, et al. Irritable bowel syndrome symptom severity improves equally with probiotic and placebo. *World J Gastroenterol* 2016; 22(48): 10631-42.
33. Yoon H, Park YS, Lee DH, Seo J-G, Shin CM, Kim N. Effect of administering a multi-species probiotic mixture on the changes in fecal microbiota and symptoms of irritable bowel syndrome: a randomized, double-blind, placebo-controlled trial. *J Clin Biochem Nutr.* 2015; 57(2): 129-34.
34. Wong RK, Yang C, Song G-H, Wong J, Ho K-Y. Melatonin Regulation as a Possible Mechanism for Probiotic (VSL#3) in Irritable Bowel Syndrome: A Randomized Double-Blinded Placebo Study. *Dig Dis Sci* 2015; 60(1): 186-94.
35. de Chambrun GP, Neut C, Chau A, et al. A randomized clinical trial of Saccharomyces cerevisiae versus placebo in the irritable bowel syndrome. *Dig Liver Dis.* 2015; 47(2): 119-24.
36. Yoon JS, Sohn W, Lee OY, et al. Effect of multispecies probiotics on irritable bowel syndrome: A randomized, double-blind, placebo-controlled trial. *J Gastroenterol Hepatol.* 2014; 29(1): 52-9.
37. Stevenson C, Blaauw R, Fredericks E, Visser J, Roux S. Randomized clinical trial: Effect of Lactobacillus plantarum 299 v on symptoms of irritable bowel syndrome. *Nutrition* 2014; 30(10): 1151-7.
38. Sisson G, Ayis S, Sherwood RA, Bjarnason I. Randomised clinical trial: a liquid multi-strain probiotic vs. placebo in the irritable bowel syndrome - a 12 week double-blind study. *Aliment Pharmacol Ther* 2014; 40(1): 51-62.
39. Shavakhi A, Minakari M, Farzamnia S, et al. The effects of multi-strain probiotic compound on symptoms and quality-of-life in patients with irritable bowel syndrome: A randomized placebo-controlled trial. *Adv Biomed Res.* 2014; 3: 140-.
40. Ludidi S, Jonkers DM, Koning CJ, et al. Randomized clinical trial on the effect of a multispecies probiotic on visceroperception in hypersensitive IBS patients. *Neurogastroenterol Motil.* 2014; 26(5): 705-14.

41. Lorenzo-Zuniga V, Llop E, Suarez C, et al. I.31, a new combination of probiotics, improves irritable bowel syndrome-related quality of life. *World J Gastroenterol* 2014; 20(26): 8709-16.
42. Jafari E, Vahedi H, Merat S, Momtahen S, Riahi A. Therapeutic Effects, Tolerability and Safety of a Multi-strain Probiotic in Iranian Adults with Irritable Bowel Syndrome and Bloating. *Arch Iran Med.* 2014; 17(7): 466-70.
43. Abbas Z, Yakoob J, Jafri W, et al. Cytokine and clinical response to Saccharomyces boulardii therapy in diarrhea-dominant irritable bowel syndrome: a randomized trial. *Eur J Gastroenterol Hepatol.* 2014; 26(6): 630-9.
44. Roberts LM, McCahon D, Holder R, Wilson S, Hobbs FDR. A randomised controlled trial of a probiotic 'functional food' in the management of irritable bowel syndrome. *BMC Gastroenterol.*2013; 13.
45. Lee J, Rheem S, Yun B, et al. Effects of probiotic yoghurt on symptoms and intestinal microbiota in patients with irritable bowel syndrome. *Int J Dairy Technol.* 2013; 66(2): 243-55.
46. Charbonneau D, Gibb RD, Quigley EMM. Fecal excretion of Bifidobacterium infantis 35624 and changes in fecal microbiota after eight weeks of oral supplementation with encapsulated probiotic. *Gut Microbes* 2013; 4(3): 201-11.
47. Cappello C, Tremolaterra F, Pascariello A, Ciacci C, Iovino P. A randomised clinical trial (RCT) of a symbiotic mixture in patients with irritable bowel syndrome (IBS): effects on symptoms, colonic transit and quality of life. *Int J Colorectal Dis.* 2013; 28(3): 349-58.
48. Begtrup LM, de Muckadell OBS, Kjeldsen J, Christensen Rd, Jarbol DE. Long-term treatment with probiotics in primary care patients with irritable bowel syndrome - a randomised, double-blind, placebo controlled trial. *Scand J Gastroenterol.* 2013; 48(10): 1127-35.
49. Amirimani B, Nikfam S, Albaji M, et al. Probiotic vs. Placebo in Irritable Bowel Syndrome:A Randomized Controlled Trial. *Middle East J Dig Dis.* 2013; 5(2): 98-102.
50. Kruis W, Chrubasik S, Boehm S, Stange C, Schulze J. A double-blind placebo-controlled trial to study therapeutic effects of probiotic Escherichia coli Nissle 1917 in subgroups of patients with irritable bowel syndrome. *Int J Colorectal Dis.* 2012; 27(4): 467-74.
51. Ducrotte P, Sawant P, Jayanthi V. Clinical trial: Lactobacillus plantarum 299v (DSM 9843) improves symptoms of irritable bowel syndrome. *World J Gastroenterol* 2012; 18(30): 4012-8.
52. Dapoigny M, Piche T, Ducrotte P, Lunaud B, Cardot J-M, Bernalier-Donadille A. Efficacy and safety profile of LCR35 complete freeze-dried culture in irritable bowel syndrome: A randomized, double-blind study. *World J Gastroenterol* 2012; 18(17): 2067-75.
53. Cui S, Hu Y. Multistrain probiotic preparation significantly reduces symptoms of irritable bowel syndrome in a double-blind placebo-controlled study. *Int J Clin Exp Med.* 2012; 5(3): 238-44.
54. Cha BK, Jung SM, Choi CH, et al. The Effect of a Multispecies Probiotic Mixture on the Symptoms and Fecal Microbiota in Diarrhea-dominant Irritable Bowel Syndrome A Randomized, Double-blind, Placebo-controlled Trial. *J Clin Gastroenterol.* 2012; 46(3): 220-7.
55. Sondergaard B, Olsson J, Ohlson K, Svensson U, Bytzer P, Ekesbo R. Effects of probiotic fermented milk on symptoms and intestinal flora in patients with irritable bowel syndrome: A randomized, placebo-controlled trial. *Scand J Gastroenterol.* 2011; 46(6): 663-72.
56. Michail S, Kenche H. Gut Microbiota is Not Modified by Randomized, Double-Blind, Placebo-Controlled Trial of VSL#3 in Diarrhea-Predominant Irritable Bowel Syndrome. *Probiotics Antimicrob Proteins.*2011; 3(1): 1-7.
57. Guglielmetti S, Mora D, Gschwender M, Popp K. Randomised clinical trial: Bifidobacterium bifidum MIMBb75 significantly alleviates irritable bowel syndrome and improves quality of life - a double-blind, placebo-controlled study. *Aliment Pharmacol Ther.* 2011; 33(10): 1123-32.
58. Choi CH, Jo SY, Park HJ, Chang SK, Byeon J-S, Myung S-J. A Randomized, Double-blind, Placebo-controlled Multicenter Trial of Saccharomyces boulardii in Irritable Bowel Syndrome Effect on Quality of Life. *J Clin Gastroenterol.* 2011; 45(8): 679-83.
59. Simren M, Ohman L, Olsson J, et al. Clinical trial: the effects of a fermented milk containing three probiotic bacteria in patients with irritable bowel syndrome - a randomized, double-blind, controlled study. *Aliment Pharmacol Ther* 2010; 31(2): 218-27.
60. Ligaarden SC, Axelsson L, Naterstad K, Lydersen S, Farup PG. A candidate probiotic with unfavourable effects in subjects with irritable bowel syndrome: a randomised controlled trial. *BMC Gastroenterol.*2010; 10.
61. Hun L. Original Research: Bacillus coagulans Significantly Improved Abdominal Pain and Bloating in Patients with IBS.

Postgrad Med. 2009; 121(2): 119-24.

62. Hong KS, Kang HW, Im JP, et al. Effect of Probiotics on Symptoms in Korean Adults with Irritable Bowel Syndrome. *Gut Liver.* 2009; 3(2): 101-7.
63. Enck P, Zimmermann K, Menke G, Klosterhalfen S. Randomized Controlled Treatment Trial of Irritable Bowel Syndrome with a Probiotic E.-coli Preparation (DSM17252) Compared to Placebo. *Z Gastroenterol.* 2009; 47(2): 209-14.
64. Dolin BJ. Effects of a Proprietary Bacillus Coagulans Preparation on Symptoms of Diarrhea-Predominant Irritable Bowel Syndrome. *Methods Find Exp Clin Pharmacol.* 2009; 31(10): 655-9.
65. Zeng J, Li YQ, Zuo XL, Zhen YB, Yang J, Liu CH. Clinical trial: effect of active lactic acid bacteria on mucosal barrier function in patients with diarrhoea-predominant irritable bowel syndrome. *Aliment Pharmacol Ther* 2008; 28(8): 994-1002.
66. Williams EA, Stimpson J, Wang D, et al. Clinical trial: a multistrain probiotic preparation significantly reduces symptoms of irritable bowel syndrome in a double-blind placebo-controlled study. *Aliment Pharmacol Ther* 2008; 29(1): 97-103.
67. Sinn DH, Song JH, Kim HJ, et al. Therapeutic effect of Lactobacillus acidophilus-SDC 2012, 2013 in patients with irritable bowel syndrome. *Dig Dis Sci* 2008; 53(10): 2714-8.
68. Kajander K, Myllyluoma E, Rajilic-Stojanovic M, et al. Clinical trial: multispecies probiotic supplementation alleviates the symptoms of irritable bowel syndrome and stabilizes intestinal microbiota. *Aliment Pharmacol Ther* 2008; 27(1): 48-57.
69. Enck P, Zimmermann K, Menke G, Mueller-Lissner S, Martens U, Klosterhalfen S. A mixture of Escherichia coli (DSM 17252) and Enterococcus faecalis (DSM 16440) for treatment of the irritable bowel syndrome - A randomized controlled trial with primary care physicians. *Neurogastroenterol Motil.* 2008; 20(10): 1103-9.
70. Drouault-Holowacz S, Bieuvelet S, Burckel A, Cazaubiel M, Dray X, Marteau P. A double blind randomized controlled trial of a probiotic combination in 100 patients with irritable bowel syndrome. *Gastroenterol Clin Biol.* 2008; 32(2): 147-52.
71. Andriulli A, Neri M, Loguercio C, et al. Clinical trial on the efficacy of a new symbiotic formulation, Flortec, in patients with irritable bowel syndrome - A multicenter, randomized study. *J Clin Gastroenterol.* 2008; 42(8): S218-S23.
72. Agrawal A, Houghton LA, Morris J, et al. Clinical trial: the effects of a fermented milk product containing Bifidobacterium lactis DN-173 010 on abdominal distension and gastrointestinal transit in irritable bowel syndrome with constipation. *Aliment Pharmacol Ther* 2008; 29(1): 104-14.
73. Guyonnet D, Chassany O, Ducrotte P, et al. Effect of a fermented milk containing Bifidobacterium animalis DN-173 010 on the health-related quality of life and symptoms in irritable bowel syndrome in adults in primary care: a multicentre, randomized, double-blind, controlled trial. *Aliment Pharmacol Ther* 2007; 26(3): 475-86.
74. Whorwell PJ, Altringer L, Morel J, et al. Efficacy of an encapsulated probiotic Bifidobacterium infantis 35624 in women with irritable bowel syndrome. *Am J Gastroenterol* 2006; 101(7): 1581-90.
75. O'Mahony L, McCarthy J, Kelly P, et al. Lactobacillus and Bifidobacterium in irritable bowel syndrome: Symptom responses and relationship to cytokine profiles. *Gastroenterology* 2005; 128(3): 541-51.
76. Niv E, Naftali T, Hallak R, Vaisman N. The efficacy of Lactobacillus reuteri ATCC 55730 in the treatment of patients with irritable bowel syndrome - a double blind, placebo-controlled, randomized study. *Clin Nutr.* 2005; 24(6): 925-31.
77. Kim HJ, Roque MIV, Camilleri M, et al. A randomized controlled trial of a probiotic combination VSL# 3 and placebo in irritable bowel syndrome with bloating. *Neurogastroenterol Motil.* 2005; 17(5): 687-96.
78. Kajander K, Hatakka K, Poussa T, Markkila M, Korpela R. A probiotic mixture alleviates symptoms in irritable bowel syndrome patients: a controlled 6-month intervention. *Aliment Pharmacol Ther* 2005; 22(5): 387-94.
79. Kim HJ, Camilleri M, McKinzie S, et al. A randomized controlled trial of a probiotic, VSL#3, on gut transit and symptoms in diarrhoea-predominant irritable bowel syndrome. *Aliment Pharmacol Ther* 2003; 17(7): 895-904.
80. Niedzielin K, Kordecki H, Birkenfeld B. A controlled, double-blind, randomized study on the efficacy of Lactobacillus plantarum 299V in patients with irritable bowel syndrome. *Eur J Gastroenterol Hepatol.* 2001; 13(10): 1143-7.
81. Nobaek S, Johansson ML, Molin G, Ahrne S, Jeppsson B. Alteration of intestinal microflora is associated with reduction in abdominal bloating and pain in patients with irritable bowel syndrome. *Am J Gastroenterol* 2000; 95(5): 1231-8.

Figure S1. The distribution of effect modifiers.

1. **The mean age of participants in probiotic groups.**

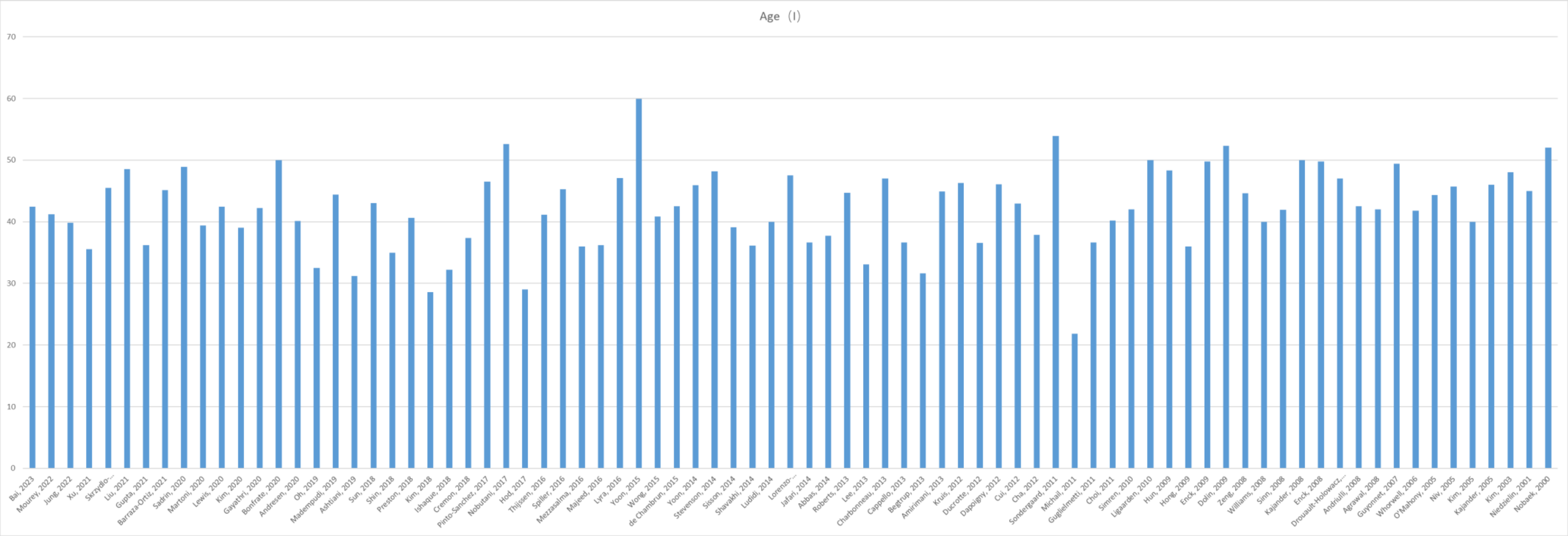

2. The mean age of participants in control groups.

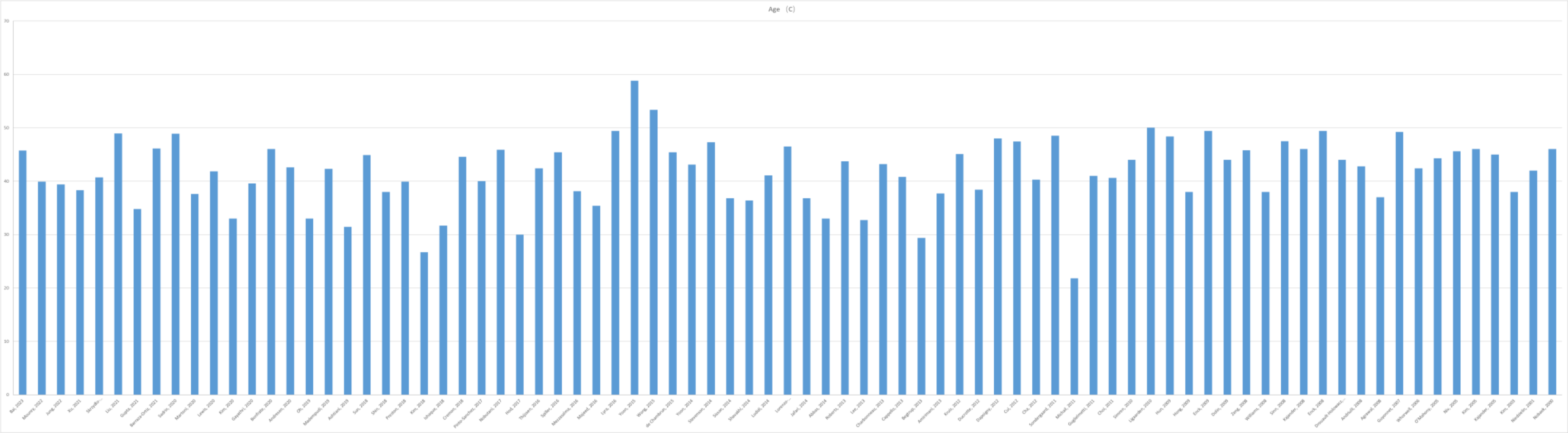

3. **The proportion of females in included studies.**

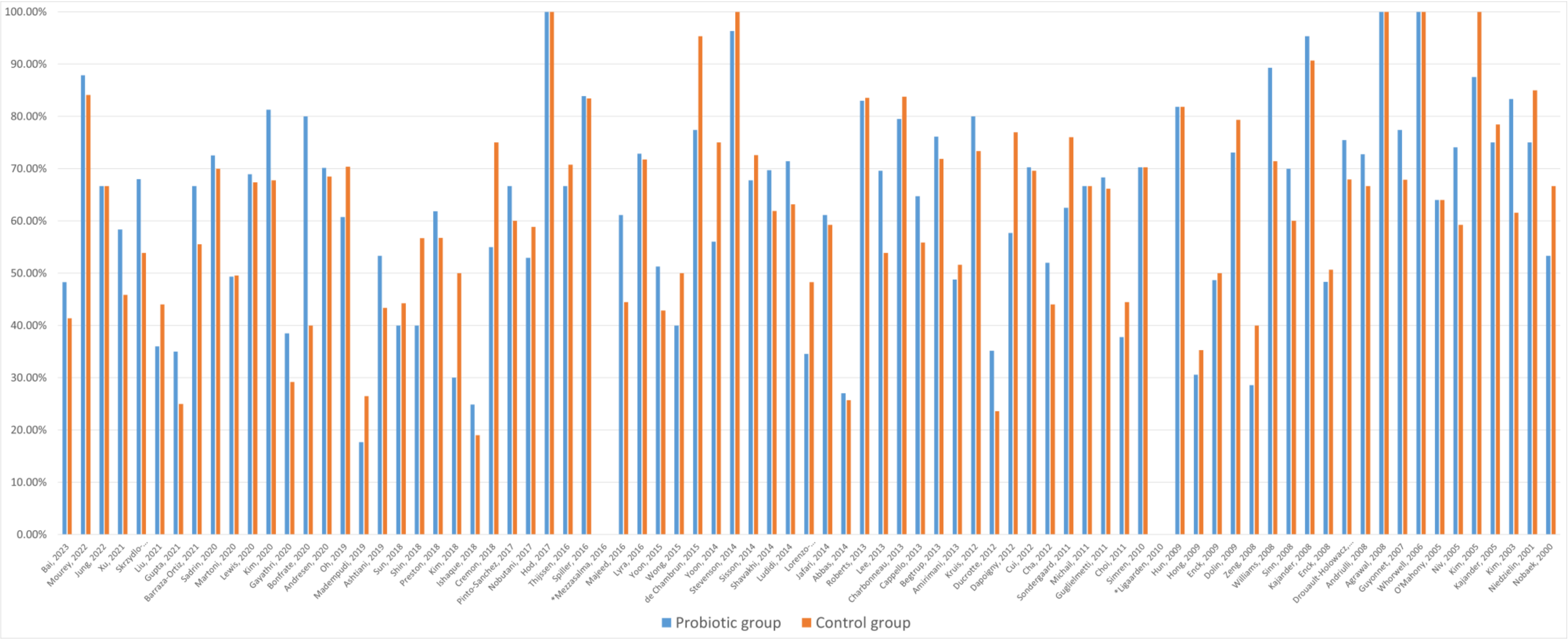

Figure S2. Risk of bias 2 evaluations for each outcome

1. **IBS-SSS (a) and IBS-QOL(b)**

a

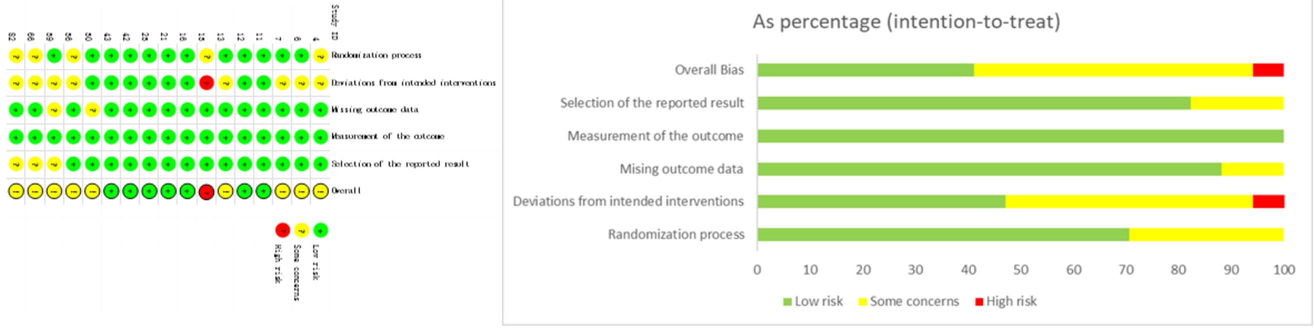

b

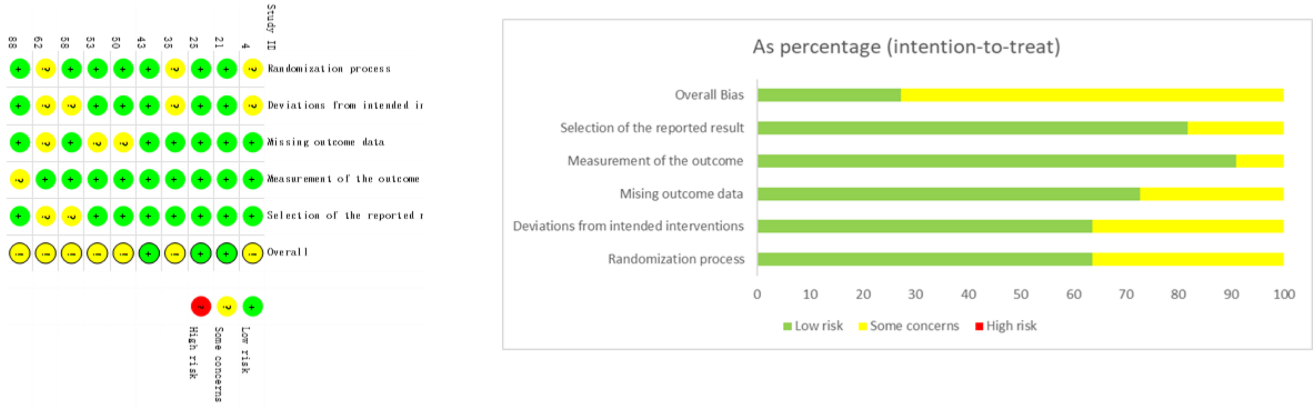

2. **HADS-total score (a), HADS-anxiety (b), and HADS-depression (c)**

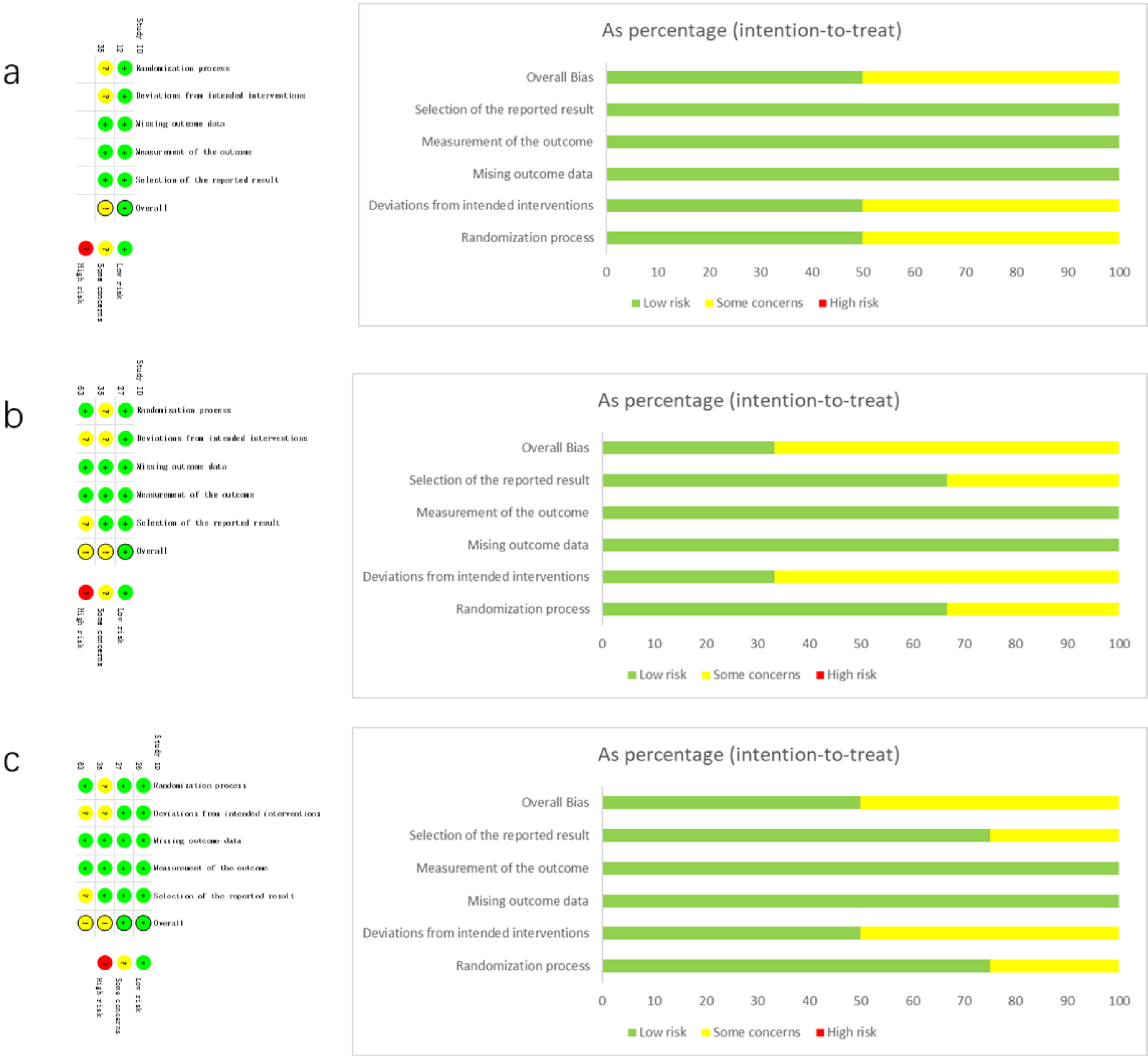

3. **Abdominal pain score (a) and abdominal bloating score (b)**

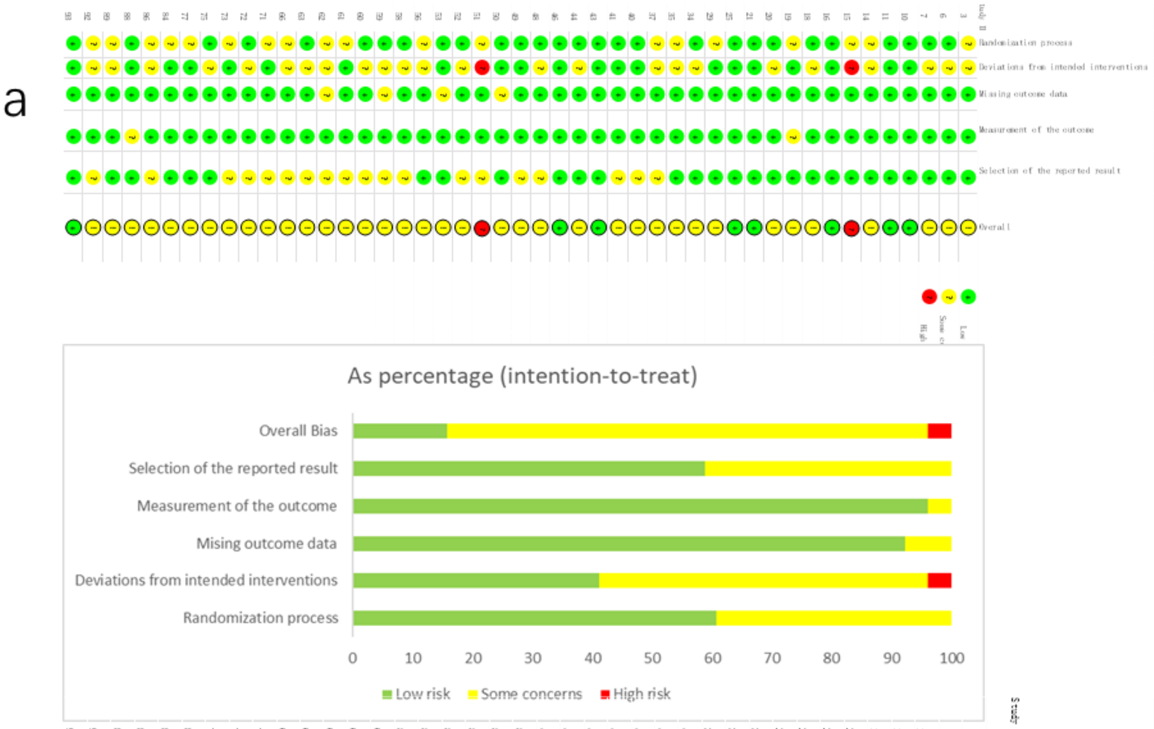

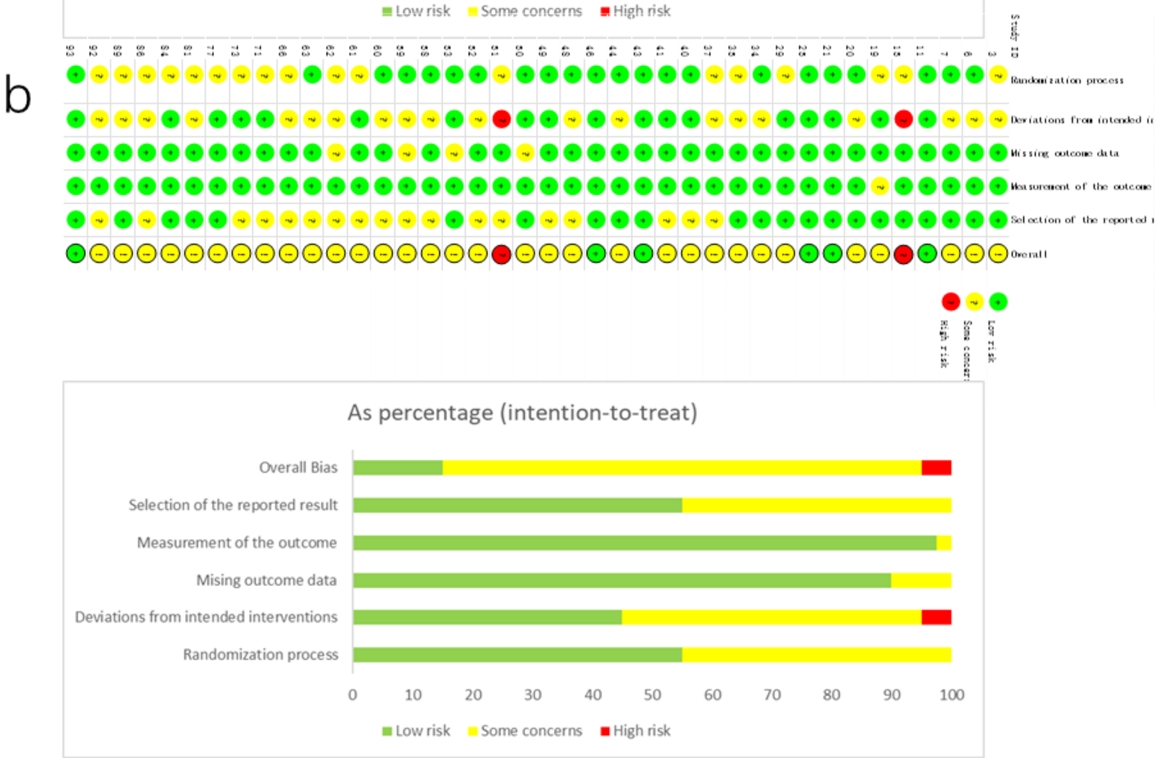

4. **Bowel movement frequency in IBS-D (a) and IBS-C (b)**

a

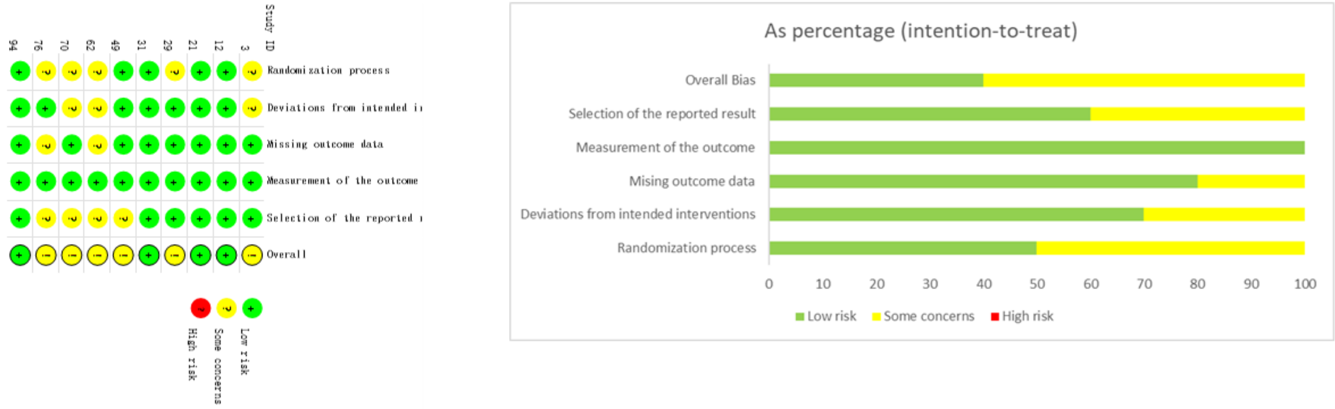

b

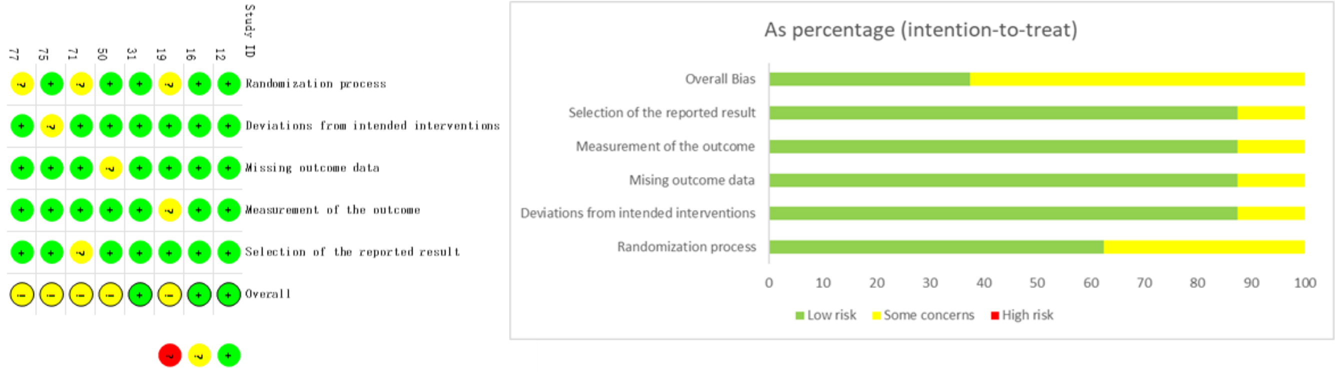

5. **Bristol stool form scale in IBS-D (a) and IBS-C (b)**

a

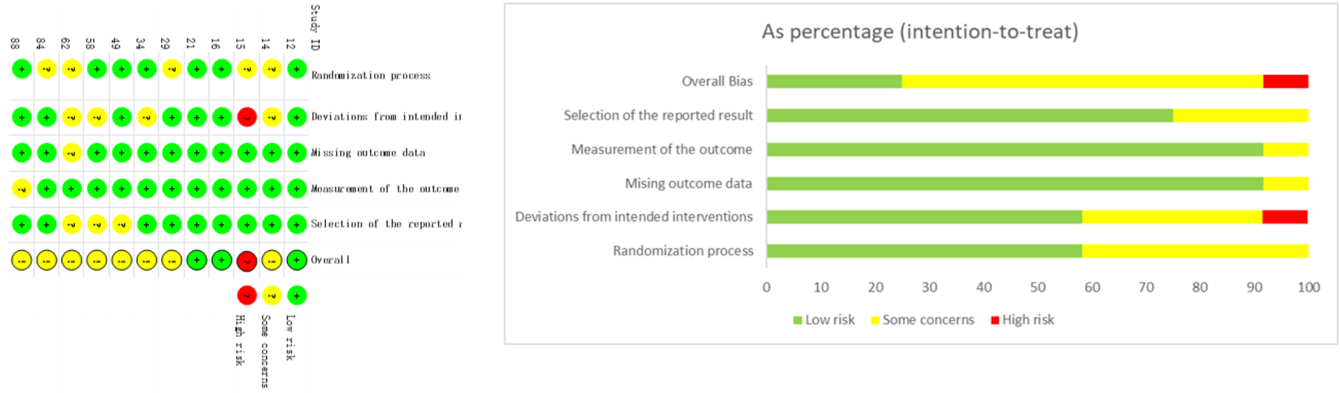

b

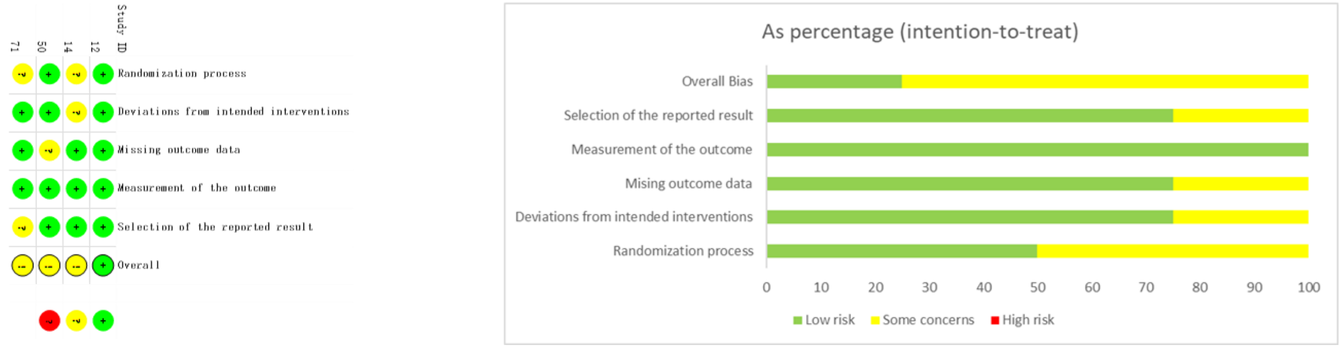

Figure S3. Network plots

Different probiotics are represented by nodes. The size of each node is proportional to the number of patients. The width of the edges represents the number of RCTs.

1. IBS-SSS

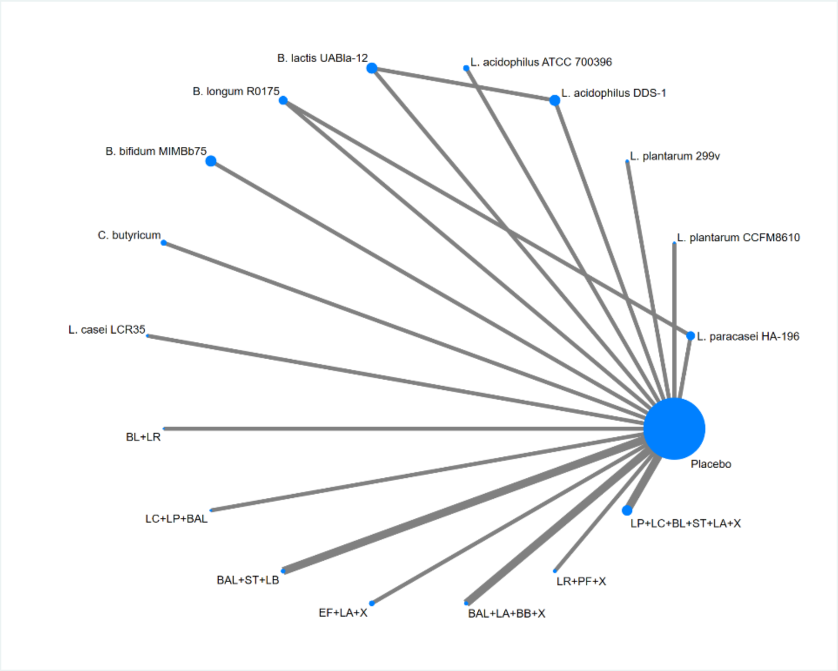

2. IBS-QOL

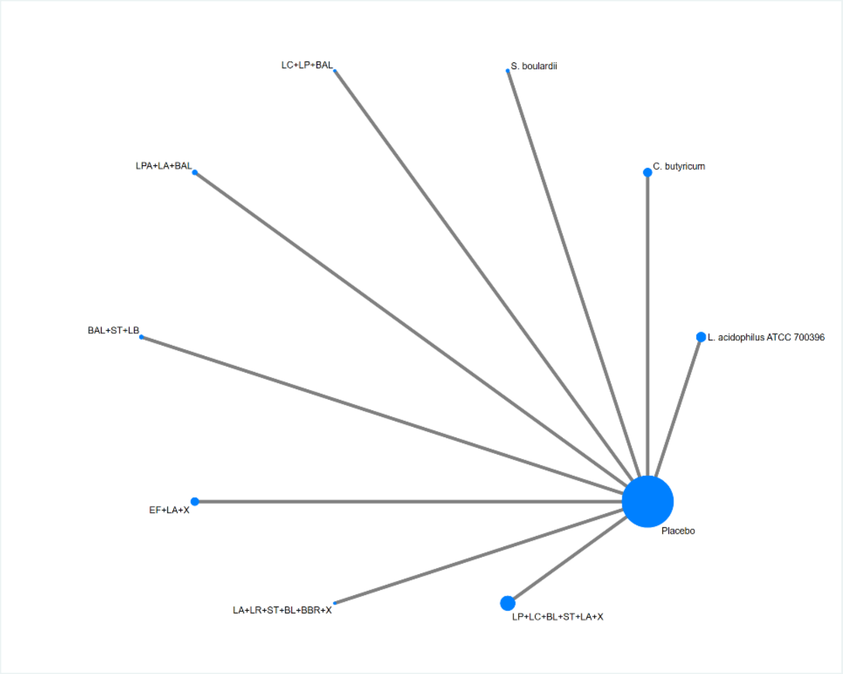

3. HADS-total score (a), HADS-anxiety (b), and HADS-depression (c)

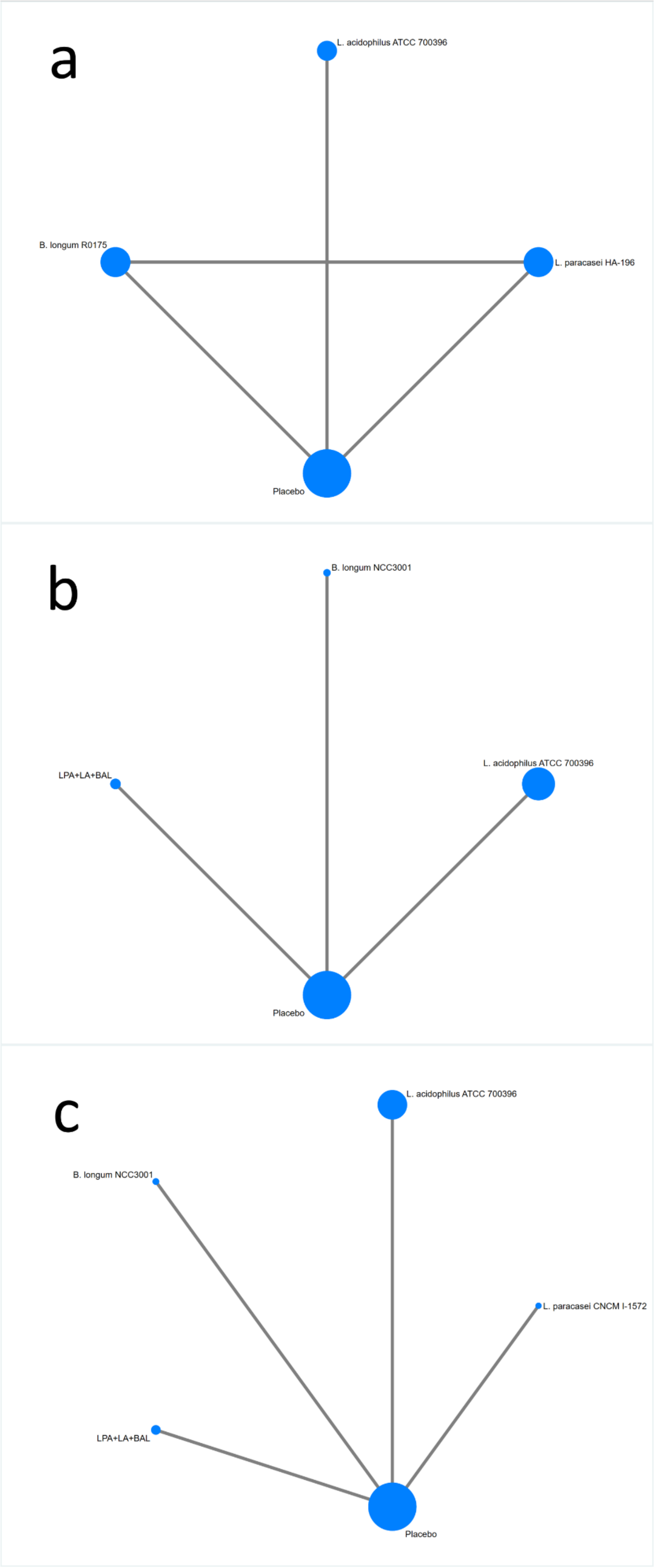

4. Abdominal pain score

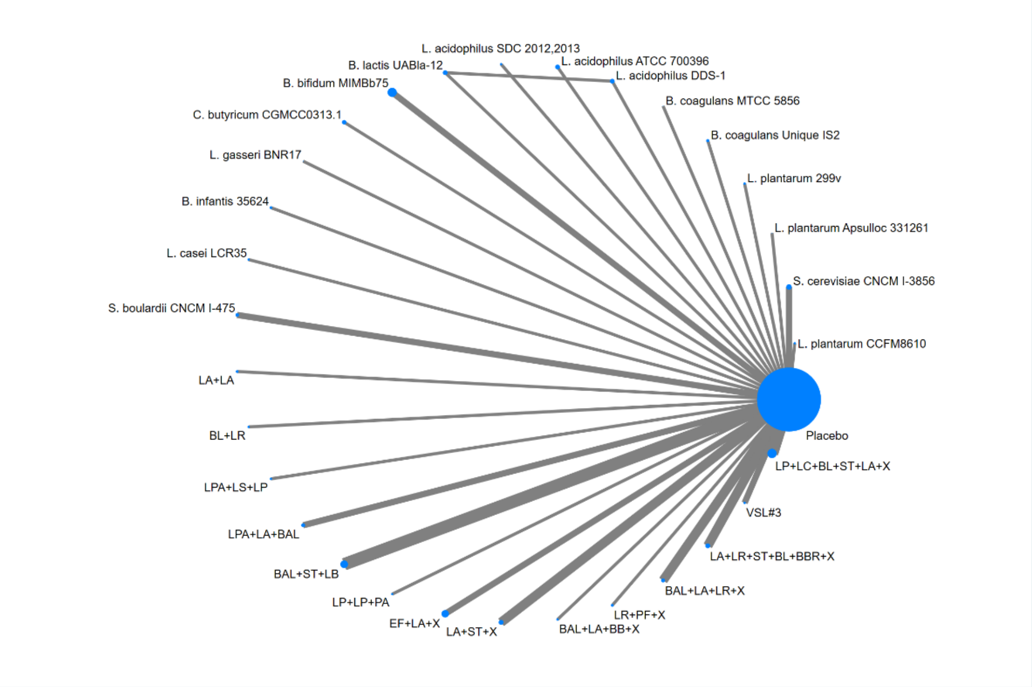

5. Abdominal bloating score

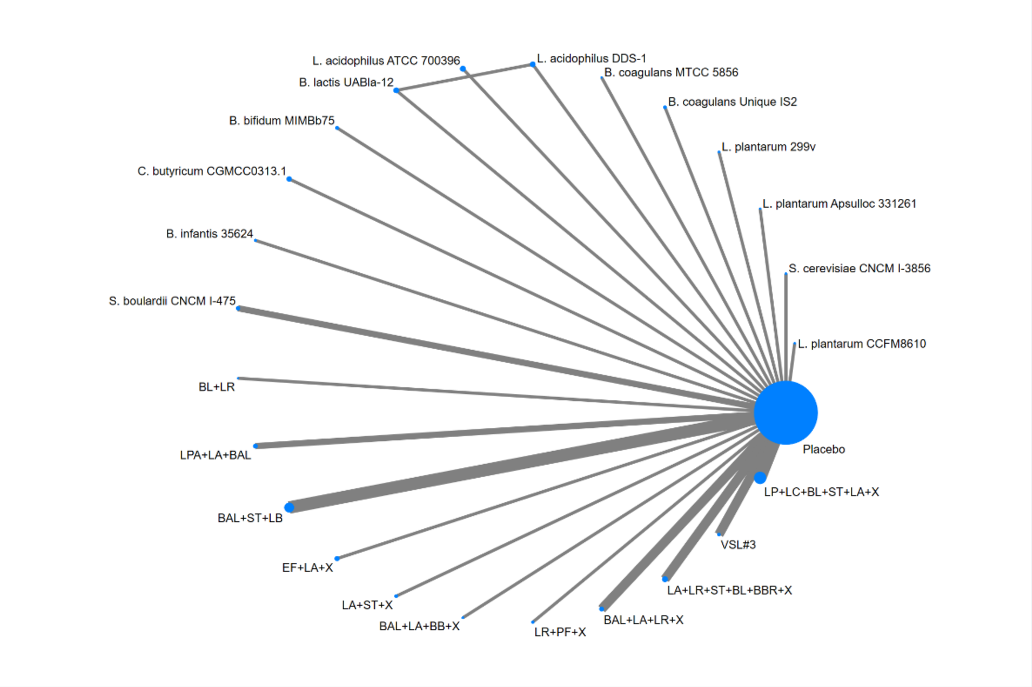

6. **Bowel movement frequency (per week) in IBS-D (a) and IBS-C (b).**

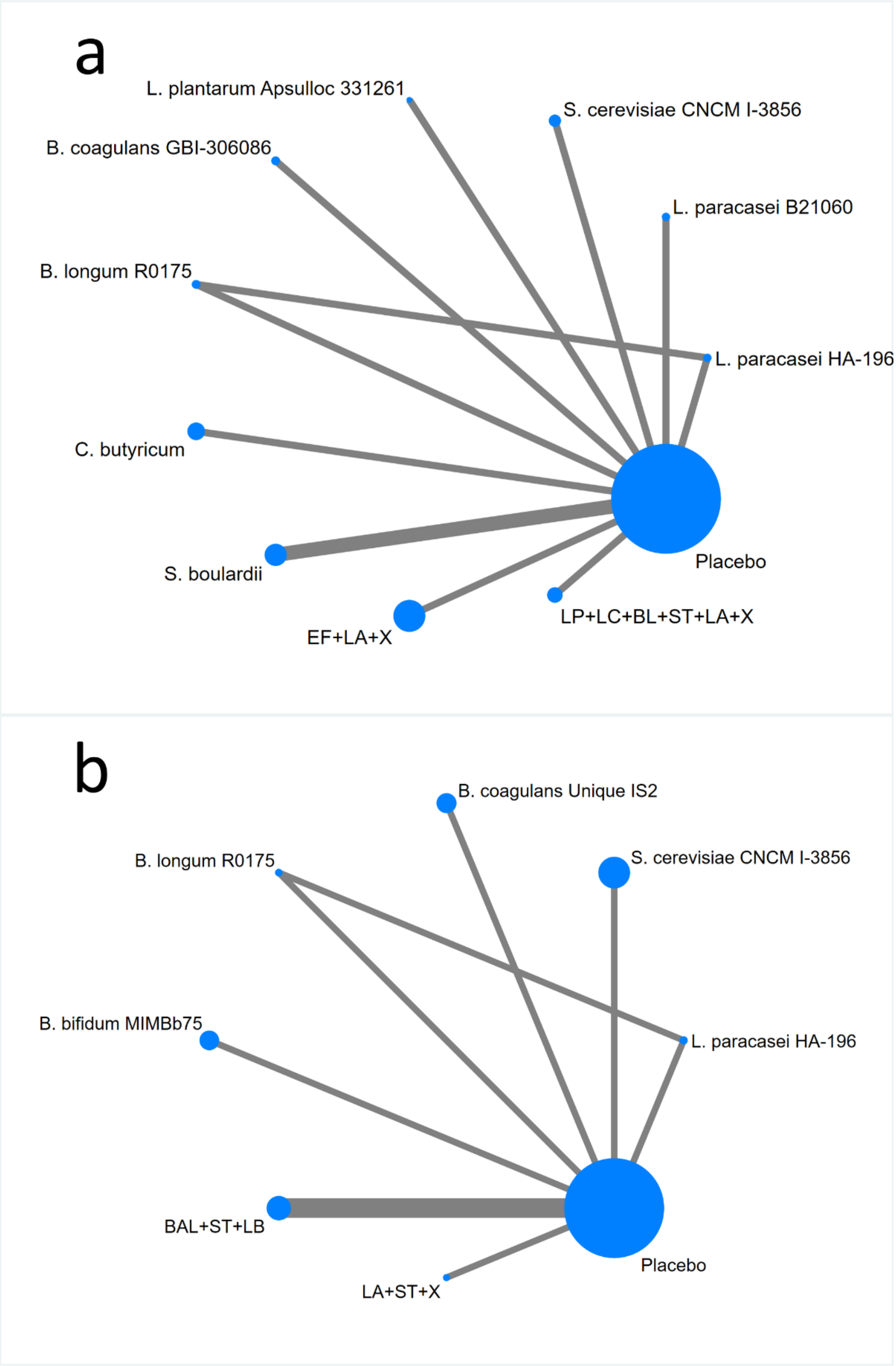

7. **Bristol stool form scale in IBS-D (a) and IBS-C (b)**

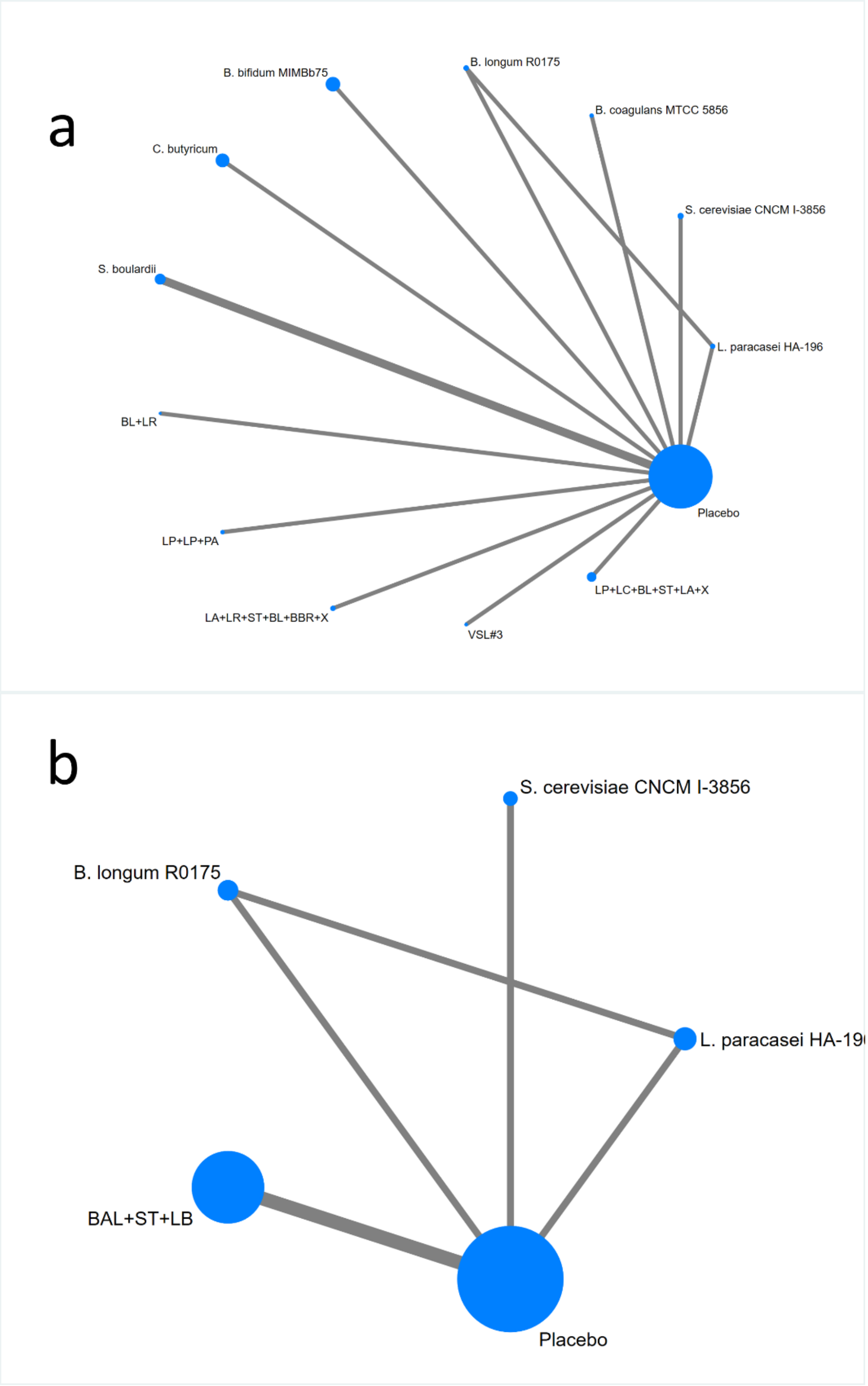

Figure S4. Cumulative Ranking Curves for each Outcome

IBS-SSS, IBS Symptom Severity Scale; IBS-QOL, IBS-Quality of Life Measure; HADS, the Hospital Anxiety and Depression Scale.

1. **IBS-SSS**

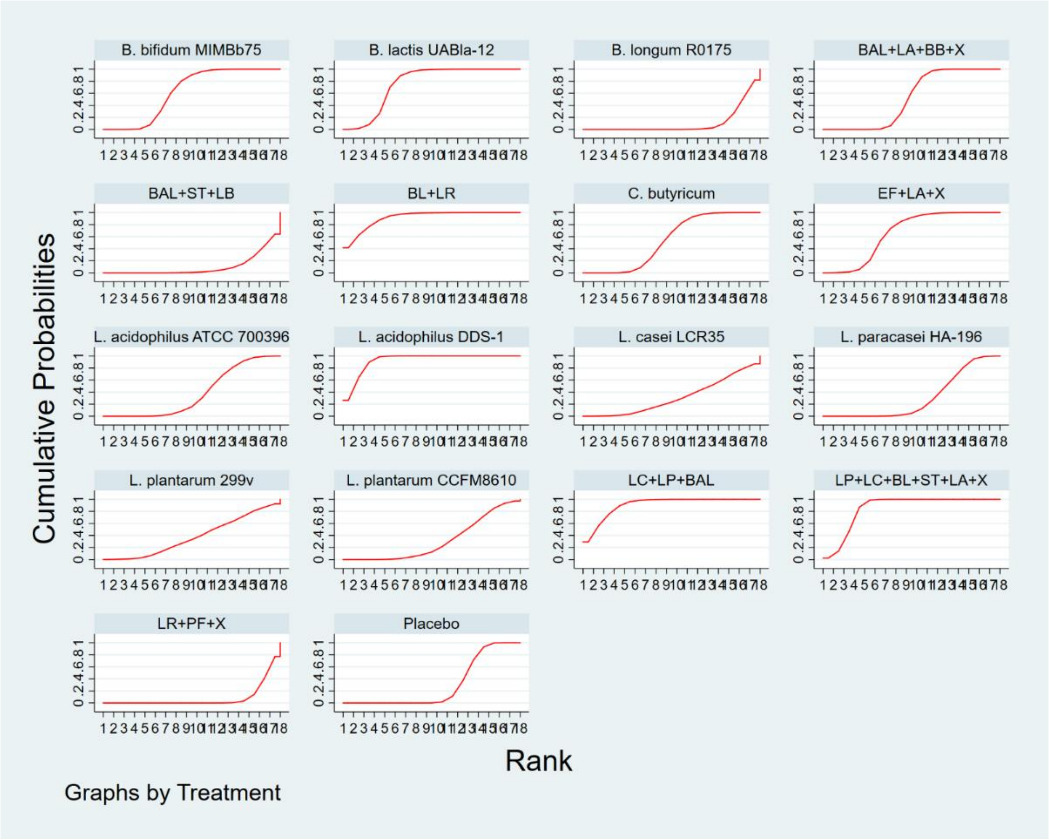

2. **IBS-QOL**

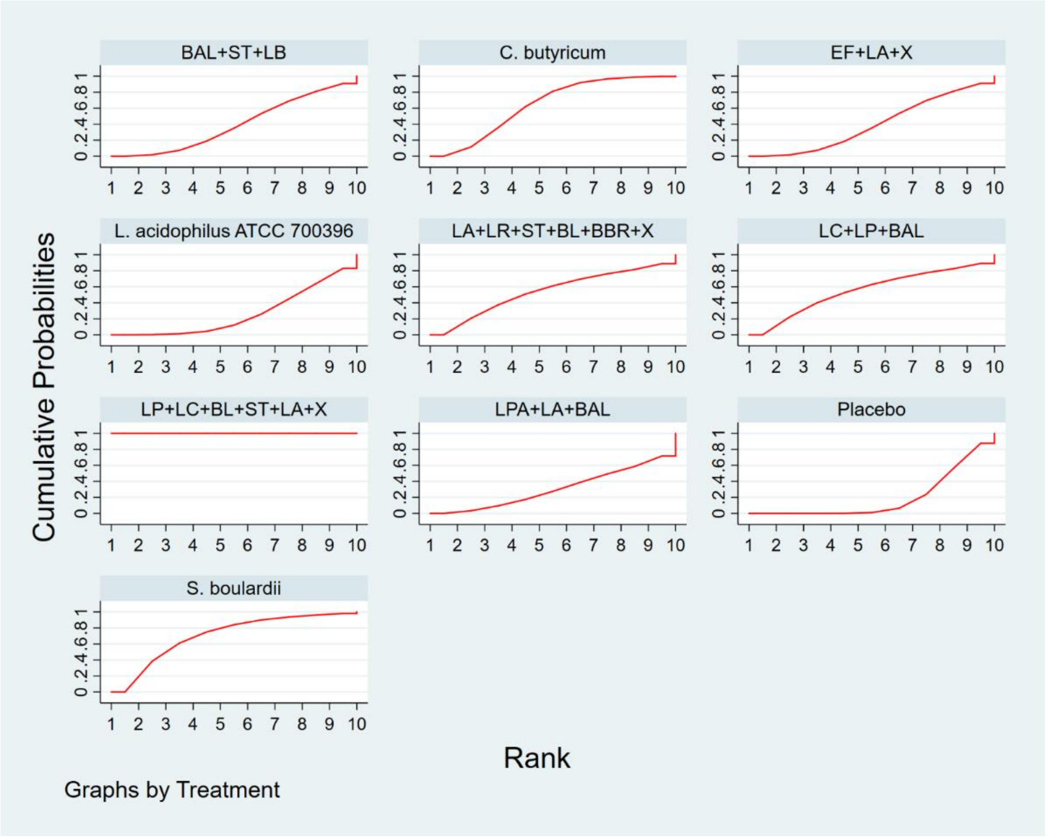

3. **HADS-total score (a), HADS-anxiety (b), and HADS-depression (c)**

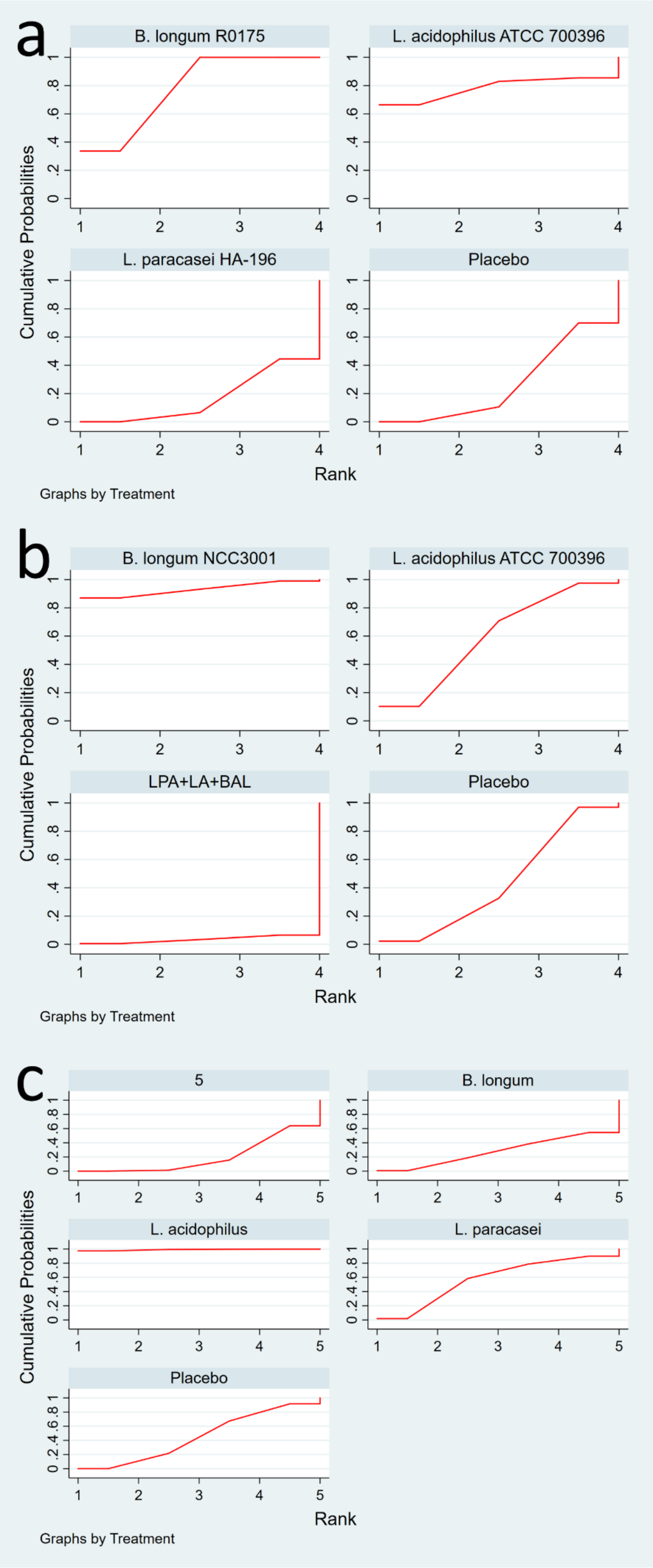

4. Abdominal pain score

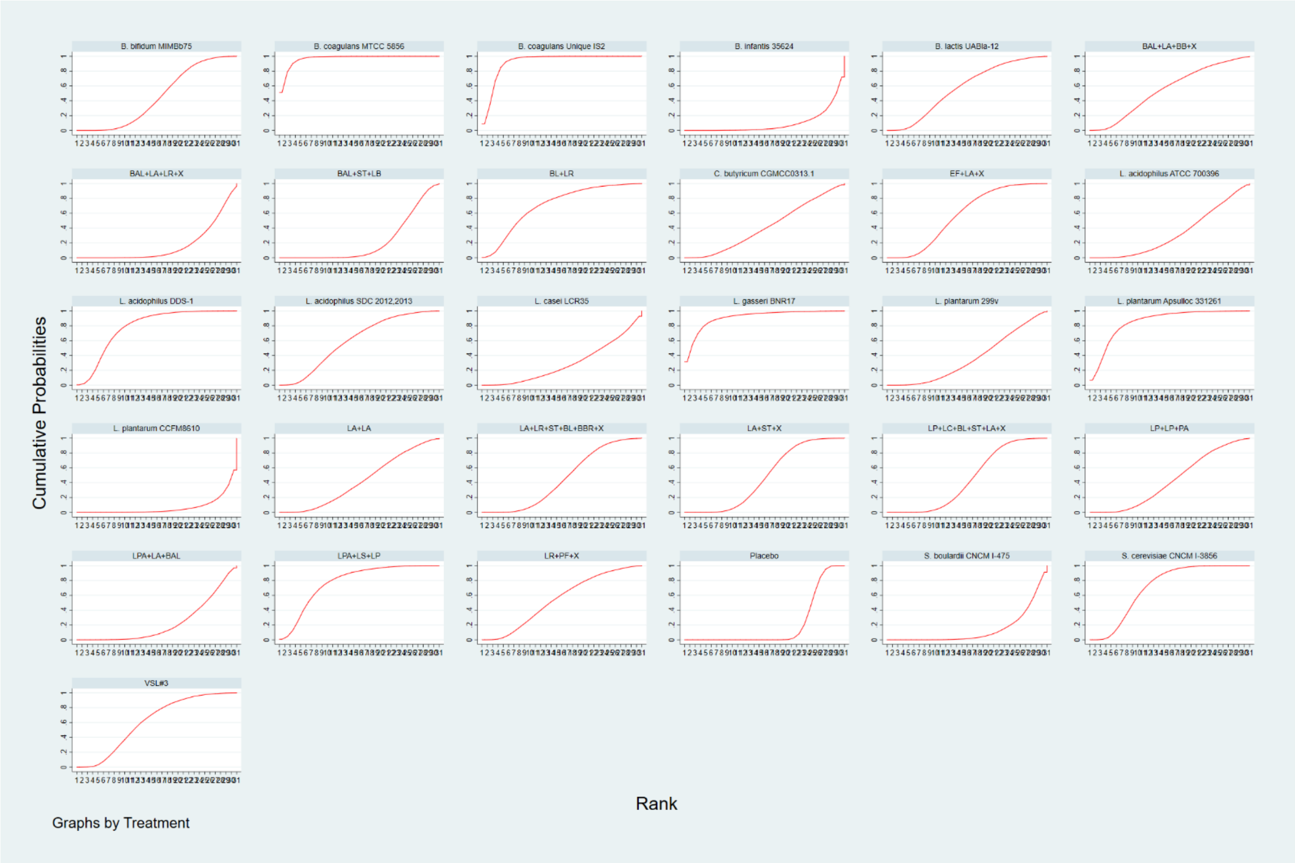

5. Abdominal bloating score

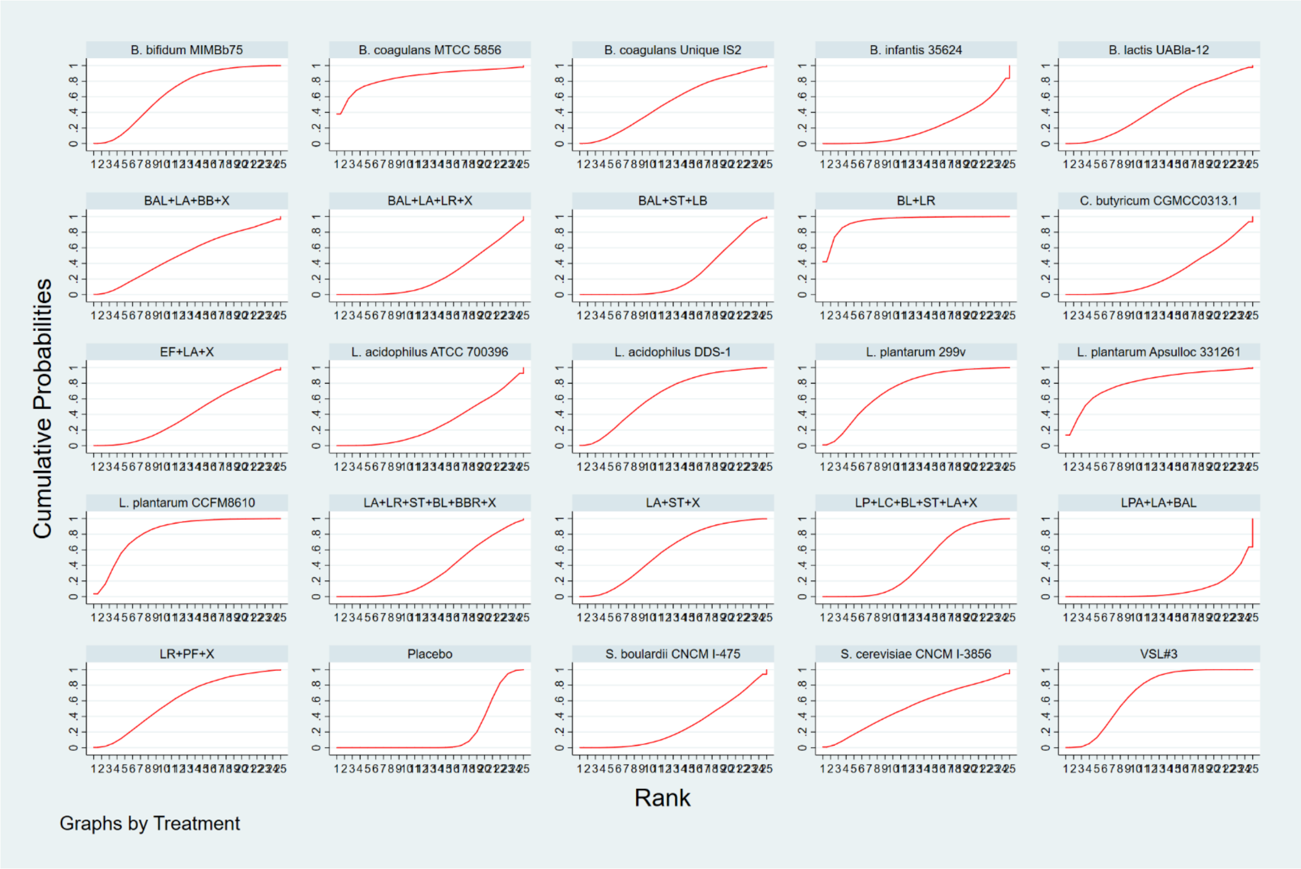

6. **Bowel movement frequency (per week) in IBS-D (a) and IBS-D (b)**

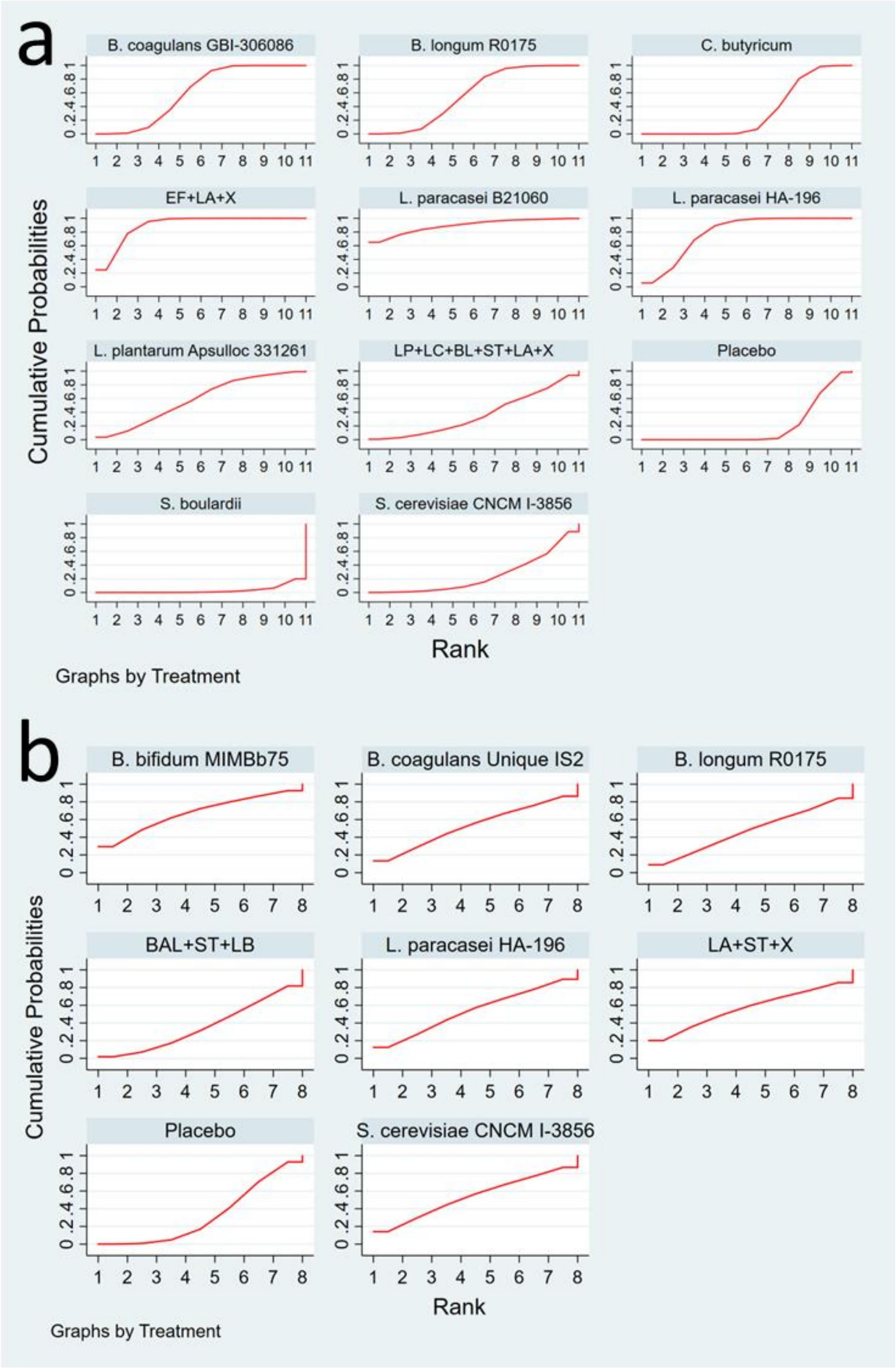

7. Bristol stool form scale in IBS-D (a) and IBS-C (b)

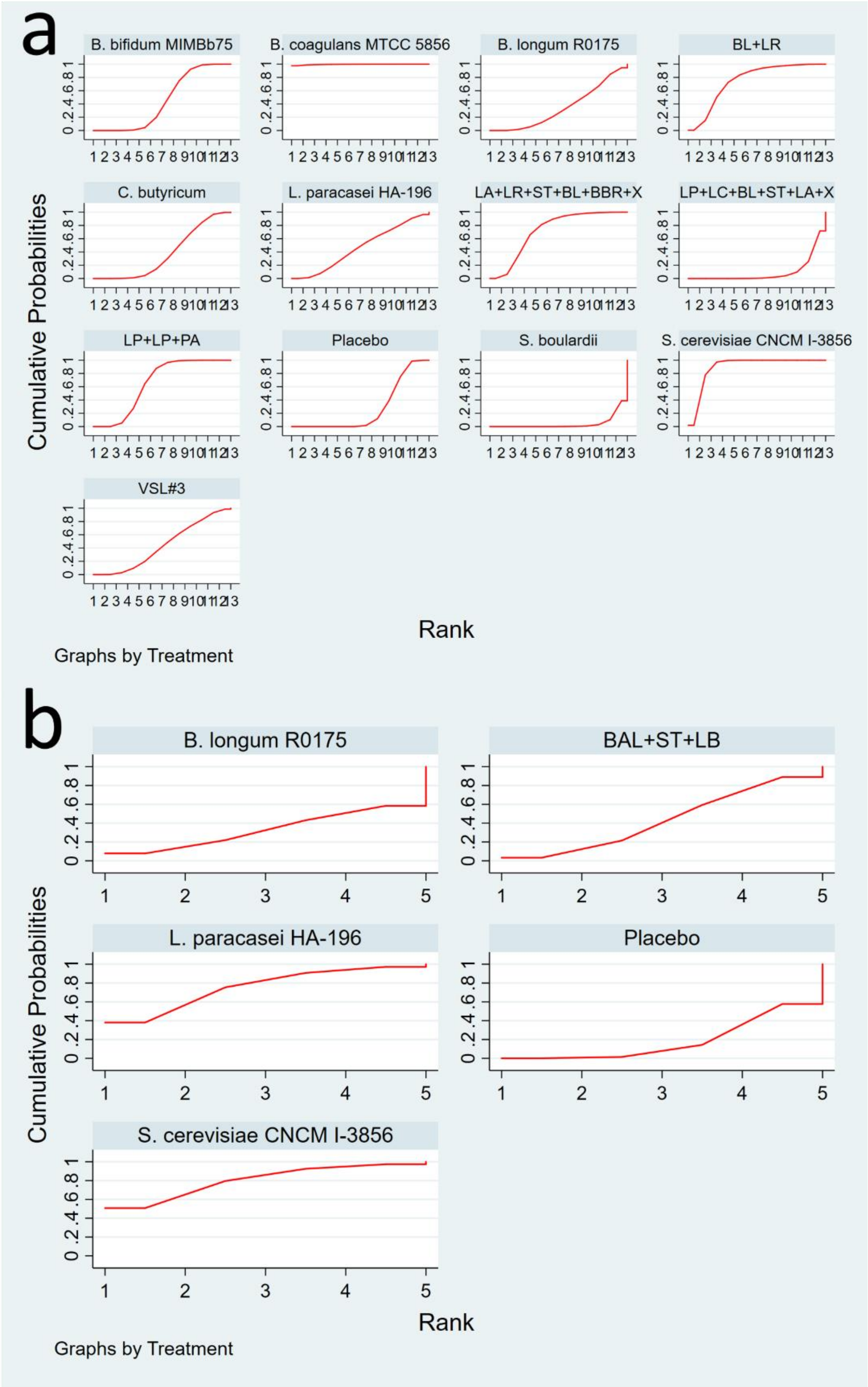

Figure S5. Predictive interval plots for each pair of interventions.

Effects were calculated as standardized mean difference and 95% confidence intervals (CI, black horizontal lines); red extensions represent predictive intervals (PrI). IBS-SSS, IBS Symptom Severity Scale; IBS-QOL, IBS-Quality of Life Measure; HADS, the Hospital Anxiety and Depression Scale.

1. **IBS-SSS**

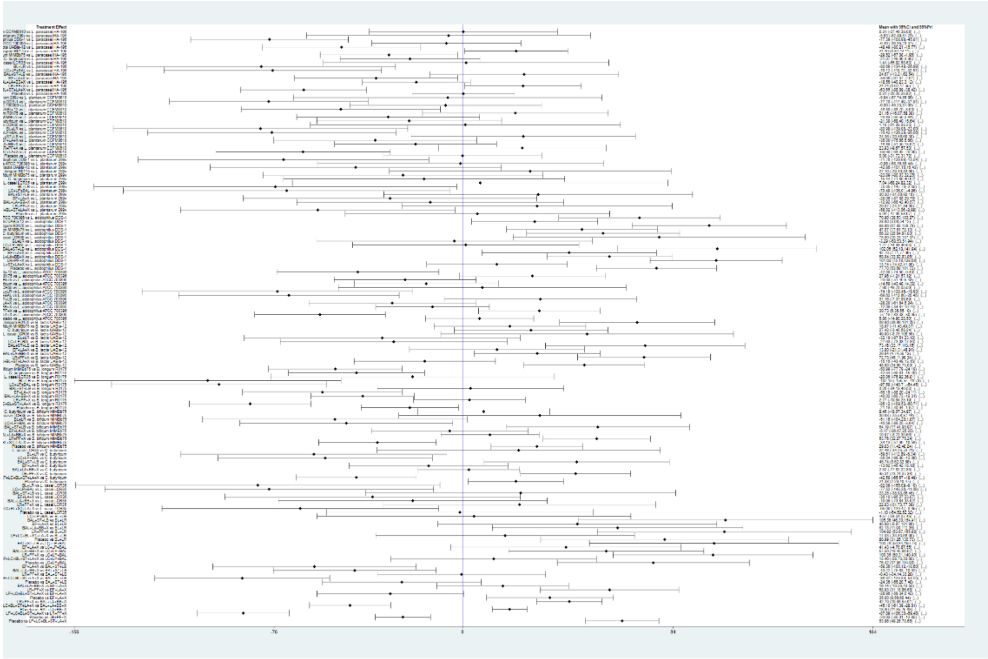

2. **IBS-QOL**

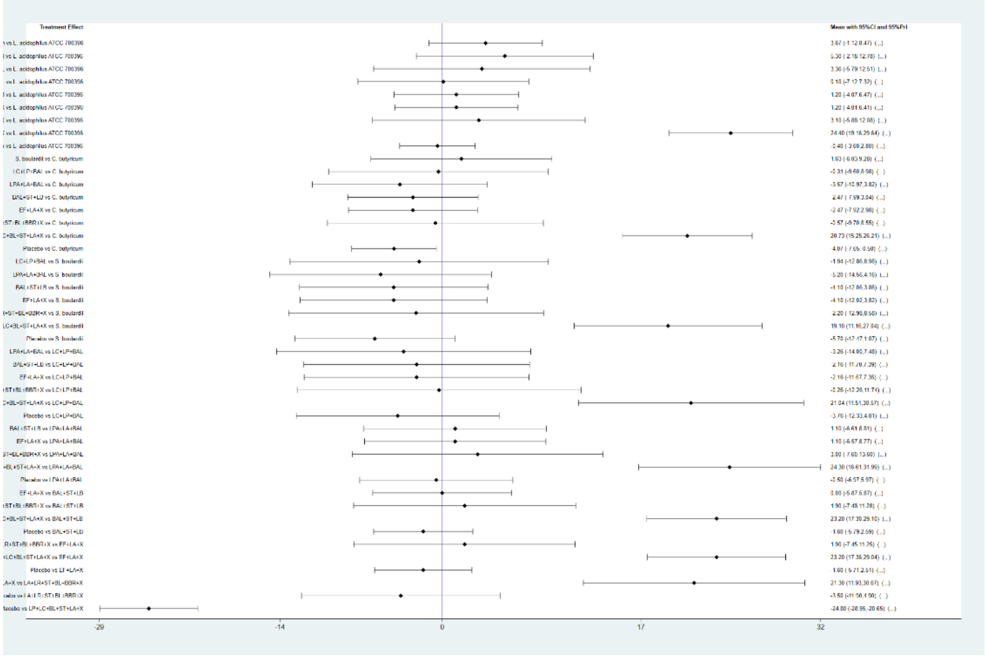

3. **HADS-total score (a), HADS-anxiety (b), and HADS-depression (c)**

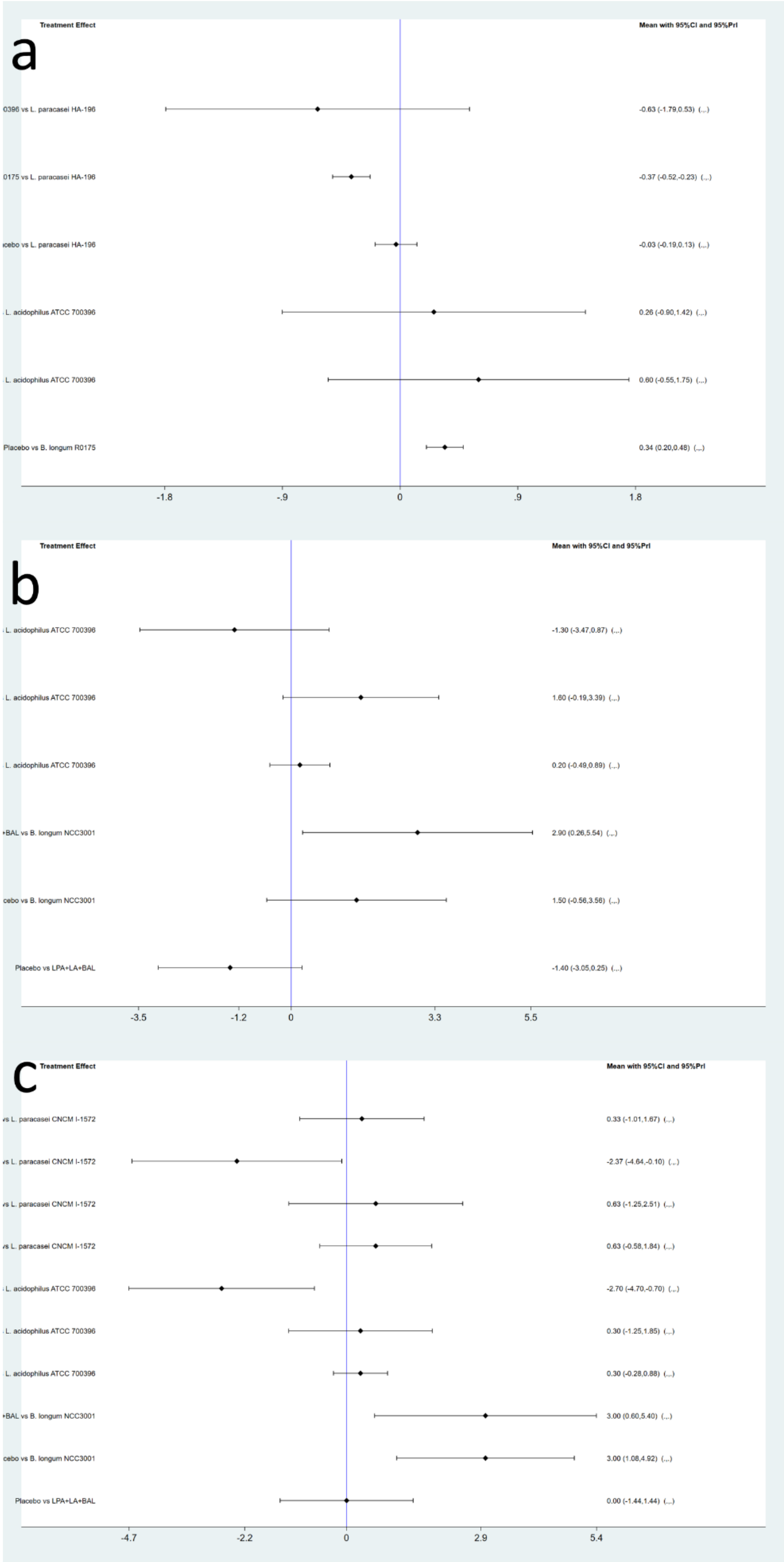

4. Abdominal pain score

_Comparison	_Effect_Size	_Standard_Error	_LCI	_UCI	_LPrI	_UPrI
S. cerevisiae CNCM I-3856 vs L. plantarum CCFM8610	-25.072	9.149122	-43.00395	-7.140047	-48.28197	-1.862025
L. plantarum Apsulloc 331261 vs L. plantarum CCFM8610	-36.4206	13.0369	-61.97245	-10.86874	-66.92573	-5.91547
L. plantarum 299v vs L. plantarum CCFM8610	-13.82707	10.43884	-34.28682	6.632679	-39.39021	11.73607
B. coagulans Unique IS2 vs L. plantarum CCFM8610	-41.827	10.35337	-62.11923	-21.53477	-67.23174	-16.42226
B. coagulans MTCC 5856 vs L. plantarum CCFM8610	-51.62402	12.75818	-76.62959	-26.61846	-81.58899	-21.65905
L. acidophilus DDS-1 vs L. plantarum CCFM8610	-29.35785	10.56205	-50.05908	-8.656614	-55.14989	-3.565807
L. acidophilus ATCC 700396 vs L. plantarum CCFM8610	-12.82841	10.25332	-32.92455	7.267725	-38.04813	12.39131
L. acidophilus SDC 2012,2013 vs L. plantarum CCFM8610	-21.82606	10.45752	-42.32243	-1.329697	-47.42387	3.771746
B. lactis UABla-12 vs L. plantarum CCFM8610	-20.93785	10.67082	-41.85227	-0.0234248	-46.9325	5.056802
B. bifidum MIMBb75 vs L. plantarum CCFM8610	-16.88433	8.948145	-34.42237	0.6537111	-39.73557	5.966905
C. butyricum CGMCC0313.1 vs L. plantarum CCFM8610	-15.87856	11.08913	-37.61285	5.855725	-42.65681	10.89968
L. gasseri BNR17 vs L. plantarum CCFM8610	-45.92713	16.47023	-78.20818	-13.64607	-83.22071	-8.633544
B. infantis 35624 vs L. plantarum CCFM8610	-2.428413	10.71917	-23.4376	18.58078	-28.51328	23.65646
L. casei LCR35 vs L. plantarum CCFM8610	-11.028	11.94749	-34.44465	12.38864	-39.43413	17.37812
S. boulardii CNCM I-475 vs L. plantarum CCFM8610	-5.968123	9.517033	-24.62116	12.68492	-29.84075	17.9045
LA+LA vs L. plantarum CCFM8610	-16.02767	10.42558	-36.46144	4.406104	-41.56622	9.510889
BL+LR vs L. plantarum CCFM8610	-26.8276	11.81	-49.97477	-3.680434	-54.97131	1.316106
LPA+LS+LP vs L. plantarum CCFM8610	-29.82715	11.27882	-51.93323	-7.721076	-56.96294	-2.69137
LPA+LA+BAL vs L. plantarum CCFM8610	-9.567669	9.685089	-28.55009	9.414758	-33.74541	14.61007
BAL+ST+LB vs L. plantarum CCFM8610	-10.41634	8.625674	-27.32235	6.489674	-32.69712	11.86444
LP+LP+PA vs L. plantarum CCFM8610	-16.81911	9.923886	-36.26957	2.631348	-41.43285	7.794628
EF+LA+X vs L. plantarum CCFM8610	-21.19558	9.403167	-39.62544	-2.765707	-44.86232	2.471169
LA+ST+X vs L. plantarum CCFM8610	-17.97355	8.662162	-34.95108	-0.9960253	-40.31855	4.371447
BAL+LA+BB+X vs L. plantarum CCFM8610	-20.95761	11.56919	-43.63282	1.71759	-48.6432	6.727975
LR+PF+X vs L. plantarum CCFM8610	-20.53776	10.73328	-41.5746	0.4990704	-46.64897	5.573442
BAL+LA+LR+X vs L. plantarum CCFM8610	-8.190621	9.273899	-26.36713	9.985886	-31.62449	15.24325
LA+LR+ST+BL+BBR+X vs L. plantarum CCFM8610	-17.45082	9.166649	-35.41712	0.5154825	-40.69219	5.790549
VSL#3 vs L. plantarum CCFM8610	-22.75667	10.12689	-42.60501	-2.908324	-47.74323	2.229898
LP+LC+BL+ST+LA+X vs L. plantarum CCFM8610	-16.73029	8.654253	-33.69232	0.231731	-39.06136	5.600778
Placebo vs L. plantarum CCFM8610	-9.828868	7.799122	-25.11487	5.457131	-30.67969	11.02195
L. plantarum Apsulloc 331261 vs S. cerevisiae CNCM I-3856	-11.3486	11.49099	-33.87053	11.17333	-38.88583	16.18863
L. plantarum 299v vs S. cerevisiae CNCM I-3856	11.24493	8.428203	-5.274047	27.7639	-10.68983	33.17969
B. coagulans Unique IS2 vs S. cerevisiae CNCM I-3856	-16.755	8.321521	-33.06488	-0.4451216	-38.50393	4.993921
B. coagulans MTCC 5856 vs S. cerevisiae CNCM I-3856	-26.55202	11.17297	-48.45064	-4.653404	-53.48813	0.3840908
L. acidophilus DDS-1 vs S. cerevisiae CNCM I-3856	-4.285849	8.579615	-21.10159	12.52989	-26.48569	17.91399
L. acidophilus ATCC 700396 vs S. cerevisiae CNCM I-3856	12.24358	8.196482	-3.821225	28.3084	-9.288541	33.77571
L. acidophilus SDC 2012,2013 vs S. cerevisiae CNCM I-3856	3.245936	8.451493	-13.31869	19.81056	-18.7295	25.22137
B. lactis UABla-12 vs S. cerevisiae CNCM I-3856	4.134151	8.71317	-12.94335	21.21165	-18.30076	26.56907
B. bifidum MIMBb75 vs S. cerevisiae CNCM I-3856	8.187666	6.492005	-4.536429	20.91176	-10.51771	26.89304
C. butyricum CGMCC0313.1 vs S. cerevisiae CNCM I-3856	9.193435	9.220546	-8.878503	27.26537	-14.14459	32.53146
L. gasseri BNR17 vs S. cerevisiae CNCM I-3856	-20.85513	15.27492	-50.79343	9.083171	-55.76105	14.0508
B. infantis 35624 vs S. cerevisiae CNCM I-3856	22.64358	8.772495	5.449809	39.83736	0.1038832	45.18328
L. casei LCR35 vs S. cerevisiae CNCM I-3856	14.04399	10.23716	-6.020471	34.10846	-11.14588	39.23387
S. boulardii CNCM I-475 vs S. cerevisiae CNCM I-3856	19.10387	7.249386	4.895339	33.31241	-0.8253265	39.03307
LA+LA vs S. cerevisiae CNCM I-3856	9.044332	8.411352	-7.441615	25.53028	-12.86102	30.94968
BL+LR vs S. cerevisiae CNCM I-3856	-1.755607	10.07627	-21.50473	17.99352	-26.64902	23.13781
LPA+LS+LP vs S. cerevisiae CNCM I-3856	-4.755157	9.448215	-23.27332	13.763	-28.50327	18.99295
LPA+LA+BAL vs S. cerevisiae CNCM I-3856	15.50433	7.482574	0.8387546	30.1699	-4.812656	35.82132
BAL+ST+LB vs S. cerevisiae CNCM I-3856	14.65566	6.039204	2.819038	26.49228	-3.347479	32.6588
LP+LP+PA vs S. cerevisiae CNCM I-3856	8.252885	7.785703	-7.006813	23.51258	-12.57514	29.08091
EF+LA+X vs S. cerevisiae CNCM I-3856	3.876422	7.126738	-10.09173	17.84457	-15.85078	23.60363
LA+ST+X vs S. cerevisiae CNCM I-3856	7.098446	6.094342	-4.846243	19.04314	-10.98891	25.1858
BAL+LA+BB+X vs S. cerevisiae CNCM I-3856	4.114383	9.792993	-15.07953	23.3083	-20.26002	28.48878
LR+PF+X vs S. cerevisiae CNCM I-3856	4.534234	8.789701	-12.69326	21.76173	-18.0359	27.10436
BAL+LA+LR+X vs S. cerevisiae CNCM I-3856	16.88138	6.923908	3.310766	30.45199	-2.514896	36.27765
LA+LR+ST+BL+BBR+X vs S. cerevisiae CNCM I-3856	7.621178	6.793944	-5.694707	20.93706	-11.56517	26.80752
VSL#3 vs S. cerevisiae CNCM I-3856	2.315331	8.040007	-13.44279	18.07345	-18.94708	23.57775
LP+LC+BL+ST+LA+X vs S. cerevisiae CNCM I-3856	8.341704	6.071534	-3.558283	20.24169	-9.71077	26.39418
Placebo vs S. cerevisiae CNCM I-3856	15.24313	4.782598	5.86941	24.61685	-0.9535642	31.43982
L. plantarum 299v vs L. plantarum Apsulloc 331261	22.59353	12.54319	-1.990667	47.17773	-6.956215	52.14327
B. coagulans Unique IS2 vs L. plantarum Apsulloc 331261	-5.406402	12.47176	-29.8506	19.03779	-34.81847	24.00566
B. coagulans MTCC 5856 vs L. plantarum Apsulloc 331261	-15.20342	14.53039	-43.68246	13.27562	-48.63406	18.22722
L. acidophilus DDS-1 vs L. plantarum Apsulloc 331261	7.062752	12.64542	-17.72182	31.84732	-22.68429	36.80979
L. acidophilus ATCC 700396 vs L. plantarum Apsulloc 331261	23.59219	12.38867	-0.6891584	47.87353	-5.659915	52.84429
L. acidophilus SDC 2012,2013 vs L. plantarum Apsulloc 331261	14.59454	12.55886	-10.02037	39.20944	-14.98542	44.1745
B. lactis UABla-12 vs L. plantarum Apsulloc 331261	15.48275	12.73641	-9.480157	40.44566	-14.44013	45.40563
B. bifidum MIMBb75 vs L. plantarum Apsulloc 331261	19.53627	11.33267	-2.675362	41.74789	-7.70126	46.77379
C. butyricum CGMCC0313.1 vs L. plantarum Apsulloc 331261	20.54204	13.08874	-5.111431	46.1955	-10.06378	51.14786
L. gasseri BNR17 vs L. plantarum Apsulloc 331261	-9.506528	17.87792	-44.54661	25.53356	-49.63728	30.62422
B. infantis 35624 vs L. plantarum Apsulloc 331261	33.99218	12.77707	8.949592	59.03477	3.990667	63.9937
L. casei LCR35 vs L. plantarum Apsulloc 331261	25.39259	13.82376	-1.701469	52.48666	-6.647738	57.43292
S. boulardii CNCM I-475 vs L. plantarum Apsulloc 331261	30.45247	11.78594	7.352456	53.55249	2.354619	58.55033
LA+LA vs L. plantarum Apsulloc 331261	20.39293	12.53187	-4.169078	44.95494	-9.134985	49.92085
BL+LR vs L. plantarum Apsulloc 331261	9.592994	13.70504	-17.26839	36.45438	-22.21479	41.40078
LPA+LS+LP vs L. plantarum Apsulloc 331261	6.593444	13.25012	-19.37631	32.5632	-24.32623	37.51312
LPA+LA+BAL vs L. plantarum Apsulloc 331261	26.85293	11.92406	3.48221	50.22365	-1.508431	55.21429
BAL+ST+LB vs L. plantarum Apsulloc 331261	26.00426	11.07967	4.288507	47.72001	-0.7561939	52.76472
LP+LP+PA vs L. plantarum Apsulloc 331261	19.60149	12.12088	-4.154996	43.35797	-9.136418	48.33939
EF+LA+X vs L. plantarum Apsulloc 331261	15.22502	11.6982	-7.70302	38.15306	-12.70575	43.15579
LA+ST+X vs L. plantarum Apsulloc 331261	18.44705	11.10916	-3.32651	40.22061	-8.368898	45.26299
BAL+LA+BB+X vs L. plantarum Apsulloc 331261	15.46298	13.49813	-10.99287	41.91883	-15.94027	46.86624
LR+PF+X vs L. plantarum Apsulloc 331261	15.88283	12.78889	-9.182927	40.9486	-14.14156	45.90723
BAL+LA+LR+X vs L. plantarum Apsulloc 331261	28.22998	11.59009	5.513815	50.94614	0.5047081	55.95525
LA+LR+ST+BL+BBR+X vs L. plantarum Apsulloc 331261	18.96978	11.50639	-3.582327	41.52188	-8.596643	46.5362
VSL#3 vs L. plantarum Apsulloc 331261	13.66393	12.28492	-10.41408	37.74194	-15.38871	42.71658
LP+LC+BL+ST+LA+X vs L. plantarum Apsulloc 331261	19.69031	11.10078	-2.066817	41.44743	-7.109859	46.49047
Placebo vs L. plantarum Apsulloc 331261	26.59173	10.44844	6.113158	47.0703	1.010769	52.17269
B. coagulans Unique IS2 vs L. plantarum 299v	-27.99993	9.72298	-47.05662	-8.943238	-52.24666	-3.7532
B. coagulans MTCC 5856 vs L. plantarum 299v	-37.79695	12.2525	-61.81141	-13.78248	-66.78733	-8.806567
L. acidophilus DDS-1 vs L. plantarum 299v	-15.53078	9.944761	-35.02215	3.960598	-40.18276	9.121211
L. acidophilus ATCC 700396 vs L. plantarum 299v	0.9986575	9.616171	-17.84869	19.84601	-23.05378	25.05109
L. acidophilus SDC 2012,2013 vs L. plantarum 299v	-7.998991	9.834454	-27.27417	11.27618	-32.44911	16.45113
B. lactis UABla-12 vs L. plantarum 299v	-7.110776	10.06021	-26.82842	12.60687	-31.97466	17.75311
B. bifidum MIMBb75 vs L. plantarum 299v	-3.057261	8.211032	-19.15059	13.03607	-24.61456	18.50004
C. butyricum CGMCC0313.1 vs L. plantarum 299v	-2.051492	10.50271	-22.63642	18.53343	-27.73321	23.63022
L. gasseri BNR17 vs L. plantarum 299v	-32.10006	16.08141	-63.61904	-0.5810708	-68.61459	4.414477
B. infantis 35624 vs L. plantarum 299v	11.39866	10.11163	-8.419776	31.21709	-13.55981	36.35713
L. casei LCR35 vs L. plantarum 299v	2.799065	11.40561	-19.55551	25.15364	-24.57642	30.17455
S. boulardii CNCM I-475 vs L. plantarum 299v	7.858947	8.826104	-9.439899	25.15779	-14.77563	30.49353
LA+LA vs L. plantarum 299v	-2.200595	9.799968	-21.40818	17.00699	-26.58773	22.18654
BL+LR vs L. plantarum 299v	-13.00053	11.26143	-35.07252	9.071453	-40.10348	14.10241
LPA+LS+LP vs L. plantarum 299v	-16.00008	10.70314	-36.97786	4.977692	-42.05503	10.05487
LPA+LA+BAL vs L. plantarum 299v	4.259402	9.009692	-13.39927	21.91807	-18.70144	27.22024
BAL+ST+LB vs L. plantarum 299v	3.410733	7.85816	-11.99098	18.81244	-17.54055	24.36201
LP+LP+PA vs L. plantarum 299v	-2.992042	9.26862	-21.1582	15.17412	-26.41642	20.43233
EF+LA+X vs L. plantarum 299v	-7.368505	8.708557	-24.43696	9.699953	-29.79528	15.05827
LA+ST+X vs L. plantarum 299v	-4.146481	7.899689	-19.62959	11.33662	-25.16859	16.87563
BAL+LA+BB+X vs L. plantarum 299v	-7.130544	11.00869	-28.70717	14.44608	-33.75758	19.49649
LR+PF+X vs L. plantarum 299v	-6.710693	10.12656	-26.5584	13.13701	-31.69666	18.27527
BAL+LA+LR+X vs L. plantarum 299v	5.636449	8.562828	-11.14639	22.41928	-16.53393	27.80683
LA+LR+ST+BL+BBR+X vs L. plantarum 299v	-3.623749	8.44917	-20.18382	12.93632	-25.59513	18.34763
VSL#3 vs L. plantarum 299v	-8.929596	9.482138	-27.51424	9.655053	-32.73906	14.87986
LP+LC+BL+ST+LA+X vs L. plantarum 299v	-2.903223	7.887902	-18.36323	12.55678	-23.90522	18.09877
Placebo vs L. plantarum 299v	3.998202	6.939856	-9.603664	17.60007	-15.42395	23.42035
B. coagulans MTCC 5856 vs B. coagulans Unique IS2	-9.797019	12.17937	-33.66814	14.0741	-38.64705	19.05301
L. acidophilus DDS-1 vs B. coagulans Unique IS2	12.46915	9.854513	-6.845337	31.78365	-12.01763	36.95594
L. acidophilus ATCC 700396 vs B. coagulans Unique IS2	28.99859	9.522809	10.33422	47.66295	5.115498	52.88168
L. acidophilus SDC 2012,2013 vs B. coagulans Unique IS2	20.00094	9.743184	0.9046479	39.09723	-4.282609	44.28448
B. lactis UABla-12 vs B. coagulans Unique IS2	20.88915	9.971007	1.34634	40.43197	-3.81095	45.58926
B. bifidum MIMBb75 vs B. coagulans Unique IS2	24.94267	8.101495	9.064031	40.82131	3.574485	46.31085
C. butyricum CGMCC0313.1 vs B. coagulans Unique IS2	25.94844	10.41729	5.530919	46.36596	0.4252583	51.47162
L. gasseri BNR17 vs B. coagulans Unique IS2	-4.100126	16.02576	-35.51003	27.30978	-40.50333	32.30308
B. infantis 35624 vs B. coagulans Unique IS2	39.39859	10.02289	19.75409	59.04308	14.60327	64.1939
L. casei LCR35 vs B. coagulans Unique IS2	30.799	11.327	8.598478	52.99952	3.572184	58.02581
S. boulardii CNCM I-475 vs B. coagulans Unique IS2	35.85888	8.72429	18.75958	52.95817	13.40434	58.31341
LA+LA vs B. coagulans Unique IS2	25.79934	9.708374	6.771273	44.8274	1.579211	50.01946
BL+LR vs B. coagulans Unique IS2	14.9994	11.18181	-6.916546	36.91534	-11.95337	41.95217
LPA+LS+LP vs B. coagulans Unique IS2	11.99985	10.61934	-8.813683	32.81338	-13.89886	37.89855
LPA+LA+BAL vs B. coagulans Unique IS2	32.25933	8.909983	14.79609	49.72258	9.475942	55.04272
BAL+ST+LB vs B. coagulans Unique IS2	31.41066	7.743633	16.23342	46.58791	10.65402	52.16731
LP+LP+PA vs B. coagulans Unique IS2	25.00789	9.171721	7.031644	42.98413	1.75743	48.25834
EF+LA+X vs B. coagulans Unique IS2	20.63142	8.605366	3.765218	37.49763	-1.613651	42.8765
LA+ST+X vs B. coagulans Unique IS2	23.85345	7.785773	8.593615	39.11328	3.025309	44.68159
BAL+LA+BB+X vs B. coagulans Unique IS2	20.86939	10.92723	-0.5475874	42.28636	-5.604771	47.34354
LR+PF+X vs B. coagulans Unique IS2	21.28924	10.03795	1.615213	40.96326	-3.533748	46.11222
BAL+LA+LR+X vs B. coagulans Unique IS2	33.63638	8.457843	17.05931	50.21345	11.64985	55.62291
LA+LR+ST+BL+BBR+X vs B. coagulans Unique IS2	24.37618	8.342762	8.024667	40.7277	2.590318	46.16204
VSL#3 vs B. coagulans Unique IS2	19.07033	9.387444	0.6712803	37.46939	-4.568038	42.70871
LP+LC+BL+ST+LA+X vs B. coagulans Unique IS2	25.09671	7.773809	9.860321	40.33309	4.288879	45.90453
Placebo vs B. coagulans Unique IS2	31.99813	6.809901	18.65097	45.34529	12.7861	51.21016
L. acidophilus DDS-1 vs B. coagulans MTCC 5856	22.26617	12.35714	-1.95338	46.48573	-6.925283	51.45763
L. acidophilus ATCC 700396 vs B. coagulans MTCC 5856	38.7956	12.09427	15.09127	62.49994	10.10867	67.48254
L. acidophilus SDC 2012,2013 vs B. coagulans MTCC 5856	29.79796	12.26854	5.752056	53.84386	0.7767763	58.81914
B. lactis UABla-12 vs B. coagulans MTCC 5856	30.68617	12.45024	6.28415	55.0882	1.31555	60.0568
B. bifidum MIMBb75 vs B. coagulans MTCC 5856	34.73969	11.01007	13.16034	56.31903	8.110051	61.36933
C. butyricum CGMCC0313.1 vs B. coagulans MTCC 5856	35.74546	12.81045	10.63744	60.85347	5.679345	65.81157
L. gasseri BNR17 vs B. coagulans MTCC 5856	5.696893	17.67519	-28.94585	40.33963	-34.02378	45.41756
B. infantis 35624 vs B. coagulans MTCC 5856	49.19561	12.49183	24.71207	73.67914	19.74487	78.64634
L. casei LCR35 vs B. coagulans MTCC 5856	40.59602	13.56055	14.01783	67.1742	9.070826	72.12121
S. boulardii CNCM I-475 vs B. coagulans MTCC 5856	45.6559	11.4761	23.16316	68.14863	18.1469	73.16489
LA+LA vs B. coagulans MTCC 5856	35.59635	12.24092	11.6046	59.58811	6.628218	64.56448
BL+LR vs B. coagulans MTCC 5856	24.79642	13.43951	-1.544534	51.13736	-6.492403	56.08523
LPA+LS+LP vs B. coagulans MTCC 5856	21.79687	12.97528	-3.63422	47.22795	-8.588675	52.18241
LPA+LA+BAL vs B. coagulans MTCC 5856	42.05635	11.61788	19.28572	64.82699	14.27829	69.83442
BAL+ST+LB vs B. coagulans MTCC 5856	41.20768	10.74948	20.13909	62.27628	15.06621	67.34916
LP+LP+PA vs B. coagulans MTCC 5856	34.8049	11.81981	11.6385	57.97131	6.642483	62.96733
EF+LA+X vs B. coagulans MTCC 5856	30.42844	11.38594	8.112404	52.74448	3.090169	57.76672
LA+ST+X vs B. coagulans MTCC 5856	33.65047	10.77988	12.5223	54.77864	7.452191	59.84874
BAL+LA+BB+X vs B. coagulans MTCC 5856	30.6664	13.22845	4.739124	56.59369	-0.2110843	61.54389
LR+PF+X vs B. coagulans MTCC 5856	31.08626	12.50392	6.579026	55.59349	1.612218	60.5603
BAL+LA+LR+X vs B. coagulans MTCC 5856	43.4334	11.27487	21.33506	65.53173	16.30507	70.56172
LA+LR+ST+BL+BBR+X vs B. coagulans MTCC 5856	34.1732	11.1888	12.24356	56.10284	7.207257	61.13914
VSL#3 vs B. coagulans MTCC 5856	28.86735	11.98798	5.371349	52.36336	0.3838335	57.35087
LP+LC+BL+ST+LA+X vs B. coagulans MTCC 5856	34.89373	10.77124	13.78248	56.00497	8.711593	61.07586
Placebo vs B. coagulans MTCC 5856	41.79515	10.09764	22.00415	61.58615	16.86243	66.72787
L. acidophilus ATCC 700396 vs L. acidophilus DDS-1	16.52943	9.749146	-2.578539	35.63741	-7.764979	40.82385
L. acidophilus SDC 2012,2013 vs L. acidophilus DDS-1	7.531785	9.964516	-11.99831	27.06188	-17.15642	32.21999
B. lactis UABla-12 vs L. acidophilus DDS-1	8.42	7.35673	-5.998925	22.83892	-11.68711	28.52711
B. bifidum MIMBb75 vs L. acidophilus DDS-1	12.47352	8.366368	-3.924266	28.8713	-9.353436	34.30046
C. butyricum CGMCC0313.1 vs L. acidophilus DDS-1	13.47928	10.62459	-7.344532	34.3031	-12.4292	39.38776
L. gasseri BNR17 vs L. acidophilus DDS-1	-16.56928	16.16128	-48.2448	15.10624	-53.24366	20.1051
B. infantis 35624 vs L. acidophilus DDS-1	26.92943	10.23817	6.862983	46.99588	1.737685	52.12118
L. casei LCR35 vs L. acidophilus DDS-1	18.32984	11.51794	-4.244905	40.90459	-9.258488	45.91817
S. boulardii CNCM I-475 vs L. acidophilus DDS-1	23.38972	8.970802	5.807273	40.97217	0.4981641	46.28128
LA+LA vs L. acidophilus DDS-1	13.33018	9.930481	-6.133204	32.79356	-11.29564	37.956
BL+LR vs L. acidophilus DDS-1	2.530242	11.37518	-19.76471	24.82519	-24.78767	29.84816
LPA+LS+LP vs L. acidophilus DDS-1	-0.4693079	10.82277	-21.68155	20.74293	-26.7478	25.80919
LPA+LA+BAL vs L. acidophilus DDS-1	19.79018	9.151475	1.853618	37.72674	-3.424006	43.00436
BAL+ST+LB vs L. acidophilus DDS-1	18.94151	8.020334	3.221942	34.66108	-2.287125	40.17014
LP+LP+PA vs L. acidophilus DDS-1	12.53873	9.406508	-5.897685	30.97515	-11.13404	36.21151
EF+LA+X vs L. acidophilus DDS-1	8.162271	8.855151	-9.193507	25.51805	-14.52379	30.84833
LA+ST+X vs L. acidophilus DDS-1	11.3843	8.061026	-4.415024	27.18361	-9.914243	32.68283
BAL+LA+BB+X vs L. acidophilus DDS-1	8.400232	11.12503	-13.40442	30.20489	-18.44558	35.24604
LR+PF+X vs L. acidophilus DDS-1	8.820083	10.25292	-11.27527	28.91544	-16.3989	34.03907
BAL+LA+LR+X vs L. acidophilus DDS-1	21.16722	8.711903	4.092209	38.24224	-1.265453	43.5999
LA+LR+ST+BL+BBR+X vs L. acidophilus DDS-1	11.90703	8.600203	-4.94906	28.76311	-10.32897	34.14303
VSL#3 vs L. acidophilus DDS-1	6.60118	9.616964	-12.24772	25.45008	-17.4527	30.65506
LP+LC+BL+ST+LA+X vs L. acidophilus DDS-1	12.62755	8.049483	-3.149144	28.40425	-8.651144	33.90625
Placebo vs L. acidophilus DDS-1	19.52898	7.122969	5.568216	33.48974	-0.1920421	39.25
L. acidophilus SDC 2012,2013 vs L. acidophilus ATCC 700396	-8.997648	9.636599	-27.88503	9.889738	-33.0872	15.0919
B. lactis UABla-12 vs L. acidophilus ATCC 700396	-8.109433	9.866883	-27.44817	11.2293	-32.61884	16.39997
B. bifidum MIMBb75 vs L. acidophilus ATCC 700396	-4.055918	7.97299	-19.68269	11.57086	-25.20337	17.09154
C. butyricum CGMCC0313.1 vs L. acidophilus ATCC 700396	-3.050149	10.31767	-23.27242	17.17212	-28.38883	22.28853
L. gasseri BNR17 vs L. acidophilus ATCC 700396	-33.09871	15.96118	-64.38206	-1.815372	-69.3728	3.175371
B. infantis 35624 vs L. acidophilus ATCC 700396	10.4	9.919307	-9.041484	29.84148	-14.20535	35.00535
L. casei LCR35 vs L. acidophilus ATCC 700396	1.800408	11.23545	-20.22067	23.82149	-25.25352	28.85434
S. boulardii CNCM I-475 vs L. acidophilus ATCC 700396	6.86029	8.605104	-10.00541	23.72598	-15.38433	29.10491
LA+LA vs L. acidophilus ATCC 700396	-3.199253	9.601401	-22.01765	15.61915	-27.22487	20.82636
BL+LR vs L. acidophilus ATCC 700396	-13.99919	11.08906	-35.73335	7.734967	-40.77731	12.77893
LPA+LS+LP vs L. acidophilus ATCC 700396	-16.99874	10.52164	-37.62077	3.623288	-42.71563	8.718152
LPA+LA+BAL vs L. acidophilus ATCC 700396	3.260745	8.793283	-13.97377	20.49526	-19.31573	25.83722
BAL+ST+LB vs L. acidophilus ATCC 700396	2.412076	7.609088	-12.50146	17.32561	-18.11727	22.94142
LP+LP+PA vs L. acidophilus ATCC 700396	-3.9907	9.058414	-21.74487	13.76347	-27.03847	19.05707
EF+LA+X vs L. acidophilus ATCC 700396	-8.367163	8.484451	-24.99638	8.262055	-30.40022	13.66589
LA+ST+X vs L. acidophilus ATCC 700396	-5.145138	7.651965	-20.14271	9.852438	-25.74676	15.45648
BAL+LA+BB+X vs L. acidophilus ATCC 700396	-8.129201	10.8323	-29.36012	13.10172	-34.42553	18.16713
LR+PF+X vs L. acidophilus ATCC 700396	-7.709351	9.934528	-27.18067	11.76197	-32.34259	16.92389
BAL+LA+LR+X vs L. acidophilus ATCC 700396	4.637792	8.334855	-11.69822	20.97381	-17.13432	26.4099
LA+LR+ST+BL+BBR+X vs L. acidophilus ATCC 700396	-4.622406	8.218022	-20.72943	11.48462	-26.1918	16.94699
VSL#3 vs L. acidophilus ATCC 700396	-9.928254	9.27677	-28.11039	8.25388	-33.36728	13.51078
LP+LC+BL+ST+LA+X vs L. acidophilus ATCC 700396	-3.90188	7.639812	-18.87564	11.07188	-24.483	16.67924
Placebo vs L. acidophilus ATCC 700396	2.999545	6.656512	-10.04698	16.04607	-15.96668	21.96577
B. lactis UABla-12 vs L. acidophilus SDC 2012,2013	0.8882153	10.07974	-18.86771	20.64414	-24.01158	25.78801
B. bifidum MIMBb75 vs L. acidophilus SDC 2012,2013	4.94173	8.234949	-11.19847	21.08193	-16.65697	26.54043
C. butyricum CGMCC0313.1 vs L. acidophilus SDC 2012,2013	5.947499	10.52141	-14.67409	26.56909	-19.76898	31.66398
L. gasseri BNR17 vs L. acidophilus SDC 2012,2013	-24.10106	16.09363	-55.64401	7.441879	-60.64006	12.43793
B. infantis 35624 vs L. acidophilus SDC 2012,2013	19.39765	10.13106	-0.4588654	39.25416	-5.596593	44.39189
L. casei LCR35 vs L. acidophilus SDC 2012,2013	10.79806	11.42283	-11.59029	33.1864	-16.61004	38.20615
S. boulardii CNCM I-475 vs L. acidophilus SDC 2012,2013	15.85794	8.848349	-1.484506	33.20038	-6.816063	38.53194
LA+LA vs L. acidophilus SDC 2012,2013	5.798396	9.820013	-13.44848	25.04527	-18.62534	30.22213
BL+LR vs L. acidophilus SDC 2012,2013	-5.001543	11.27887	-27.10773	17.10464	-32.13743	22.13435
LPA+LS+LP vs L. acidophilus SDC 2012,2013	-8.001093	10.7215	-29.01485	13.01266	-34.09031	18.08813
LPA+LA+BAL vs L. acidophilus SDC 2012,2013	12.25839	9.031507	-5.443034	29.95982	-10.74135	35.25814
BAL+ST+LB vs L. acidophilus SDC 2012,2013	11.40972	7.883147	-4.04096	26.86041	-9.584156	32.4036
LP+LP+PA vs L. acidophilus SDC 2012,2013	5.006949	9.289811	-13.20075	23.21464	-18.45554	28.46943
EF+LA+X vs L. acidophilus SDC 2012,2013	0.6304858	8.731145	-16.48224	17.74322	-21.83615	23.09712
LA+ST+X vs L. acidophilus SDC 2012,2013	3.852511	7.924546	-11.67931	19.38434	-17.21206	24.91708
BAL+LA+BB+X vs L. acidophilus SDC 2012,2013	0.8684474	11.02653	-20.74316	22.48006	-25.79211	27.52901
LR+PF+X vs L. acidophilus SDC 2012,2013	1.288298	10.14596	-18.59743	21.17402	-23.73339	26.30999
BAL+LA+LR+X vs L. acidophilus SDC 2012,2013	13.63544	8.585749	-3.192317	30.4632	-8.575171	35.84605
LA+LR+ST+BL+BBR+X vs L. acidophilus SDC 2012,2013	4.375242	8.472422	-12.2304	20.98088	-17.63677	26.38726
VSL#3 vs L. acidophilus SDC 2012,2013	-0.9306051	9.502856	-19.55586	17.69465	-24.77756	22.91635
LP+LC+BL+ST+LA+X vs L. acidophilus SDC 2012,2013	5.095768	7.912782	-10.413	20.60454	-15.9487	26.14024
Placebo vs L. acidophilus SDC 2012,2013	11.99719	6.968134	-1.660099	25.65449	-7.470899	31.46529
B. bifidum MIMBb75 vs B. lactis UABla-12	4.053515	8.503274	-12.61059	20.71762	-18.01248	26.11951
C. butyricum CGMCC0313.1 vs B. lactis UABla-12	5.059284	10.73273	-15.97648	26.09505	-21.0509	31.16947
L. gasseri BNR17 vs B. lactis UABla-12	-24.96928	16.25257	-56.80454	6.825976	-61.80045	11.82787
B. infantis 35624 vs B. lactis UABla-12	18.50943	10.35035	-1.776877	38.79575	-6.889711	43.90858
L. casei LCR35 vs B. lactis UABla-12	9.909842	11.61777	-12.86056	32.68024	-17.868	37.68768
S. boulardii CNCM I-475 vs B. lactis UABla-12	14.96972	9.098618	-2.86324	32.80268	-8.149878	38.08932
LA+LA vs B. lactis UABla-12	4.910181	10.04609	-14.7798	24.60016	-19.92776	29.74813
BL+LR vs B. lactis UABla-12	-5.889758	11.47625	-28.3828	16.60328	-33.39904	21.61953
LPA+LS+LP vs B. lactis UABla-12	-8.889308	10.92895	-30.30965	12.53104	-35.36669	17.58808
LPA+LA+BAL vs B. lactis UABla-12	11.37018	9.276801	-6.812017	29.55237	-12.06891	34.80927
BAL+ST+LB vs B. lactis UABla-12	10.52151	8.163045	-5.477766	26.52078	-10.95283	31.99585
LP+LP+PA vs B. lactis UABla-12	4.118733	9.528481	-14.55675	22.79421	-19.77463	28.0121
EF+LA+X vs B. lactis UABla-12	-0.2577295	8.984612	-17.86724	17.35179	-23.17388	22.65842
LA+ST+X vs B. lactis UABla-12	2.964295	8.203029	-13.11335	19.04194	-18.57915	24.50774
BAL+LA+BB+X vs B. lactis UABla-12	-0.019768	11.22835	-22.02693	21.98739	-27.0603	27.02076
LR+PF+X vs B. lactis UABla-12	0.4000825	10.36494	-19.91482	20.71499	-25.02608	25.82624
BAL+LA+LR+X vs B. lactis UABla-12	12.74722	8.84346	-4.585639	30.08009	-9.918111	35.41256
LA+LR+ST+BL+BBR+X vs B. lactis UABla-12	3.487027	8.733443	-13.63021	20.60426	-18.98367	25.95773
VSL#3 vs B. lactis UABla-12	-1.81882	9.7363	-20.90162	17.26398	-26.08982	22.45218
LP+LC+BL+ST+LA+X vs B. lactis UABla-12	4.207553	8.191687	-11.84786	20.26296	-17.31628	25.73139
Placebo vs B. lactis UABla-12	11.10898	7.283284	-3.165997	25.38395	-8.876294	31.09425
C. butyricum CGMCC0313.1 vs B. bifidum MIMBb75	1.005769	9.022453	-16.67792	18.68945	-21.97783	23.98936
L. gasseri BNR17 vs B. bifidum MIMBb75	-29.0428	15.15617	-58.74835	0.662759	-63.71276	5.627169
B. infantis 35624 vs B. bifidum MIMBb75	14.45592	8.564048	-2.329308	31.24114	-7.716599	36.62843
L. casei LCR35 vs B. bifidum MIMBb75	5.856326	10.05911	-13.85917	25.57182	-19.00554	30.71819
S. boulardii CNCM I-475 vs B. bifidum MIMBb75	10.91621	7.000774	-2.805058	24.63747	-8.605012	30.43743
LA+LA vs B. bifidum MIMBb75	0.8566653	8.193729	-15.20275	16.91608	-20.6707	22.38403
BL+LR vs B. bifidum MIMBb75	-9.943274	9.895329	-29.33776	9.451214	-34.50472	14.61818
LPA+LS+LP vs B. bifidum MIMBb75	-12.94282	9.255006	-31.0823	5.196655	-36.34274	10.45709
LPA+LA+BAL vs B. bifidum MIMBb75	7.316663	7.228386	-6.850714	21.48404	-12.57785	27.21118
BAL+ST+LB vs B. bifidum MIMBb75	6.467994	5.731174	-4.764902	17.70089	-11.07172	24.00771
LP+LP+PA vs B. bifidum MIMBb75	0.0652184	7.5502	-14.7329	14.86334	-20.36511	20.49555
EF+LA+X vs B. bifidum MIMBb75	-4.311245	6.847157	-17.73143	9.108937	-23.58334	14.96085
LA+ST+X vs B. bifidum MIMBb75	-1.08922	5.787703	-12.43291	10.25447	-18.71306	16.53462
BAL+LA+BB+X vs B. bifidum MIMBb75	-4.073283	9.606716	-22.9021	14.75554	-28.10855	19.96198
LR+PF+X vs B. bifidum MIMBb75	-3.653433	8.581676	-20.47321	13.16634	-25.85689	18.55003
BAL+LA+LR+X vs B. bifidum MIMBb75	8.69371	6.666371	-4.372137	21.75956	-10.28825	27.67566
LA+LR+ST+BL+BBR+X vs B. bifidum MIMBb75	-0.5664883	6.517172	-13.33991	12.20693	-19.31158	18.1786
VSL#3 vs B. bifidum MIMBb75	-5.872335	7.810538	-21.18071	9.436038	-26.74256	14.99789
LP+LC+BL+ST+LA+X vs B. bifidum MIMBb75	0.1540378	5.773362	-11.16154	11.46962	-17.44842	17.75649
Placebo vs B. bifidum MIMBb75	7.055463	4.388554	-1.545945	15.65687	-8.626213	22.73714
L. gasseri BNR17 vs C. butyricum CGMCC0313.1	-30.04856	16.51046	-62.40848	2.311352	-67.42289	7.32576
B. infantis 35624 vs C. butyricum CGMCC0313.1	13.45015	10.78094	-7.680112	34.58041	-12.75012	39.65042
L. casei LCR35 vs C. butyricum CGMCC0313.1	4.850557	12.00298	-18.67485	28.37596	-23.66165	33.36276
S. boulardii CNCM I-475 vs C. butyricum CGMCC0313.1	9.910439	9.585612	-8.877016	28.69789	-14.08652	33.90739
LA+LA vs C. butyricum CGMCC0313.1	-0.1491033	10.48919	-20.70753	20.40932	-25.8057	25.5075
BL+LR vs C. butyricum CGMCC0313.1	-10.94904	11.86606	-34.20609	12.30801	-39.19968	17.3016
LPA+LS+LP vs C. butyricum CGMCC0313.1	-13.94859	11.33759	-36.16986	8.272676	-41.19542	13.29823
LPA+LA+BAL vs C. butyricum CGMCC0313.1	6.310894	9.75489	-12.80834	25.43013	-17.99399	30.61578
BAL+ST+LB vs C. butyricum CGMCC0313.1	5.462225	8.702547	-11.59445	22.5189	-16.95395	27.8784
LP+LP+PA vs C. butyricum CGMCC0313.1	-0.9405502	9.994548	-20.5295	18.6484	-25.68384	23.80274
EF+LA+X vs C. butyricum CGMCC0313.1	-5.317013	9.47744	-23.89245	13.25843	-29.11797	18.48395
LA+ST+X vs C. butyricum CGMCC0313.1	-2.094989	8.740061	-19.22519	15.03522	-24.57737	20.3874
BAL+LA+BB+X vs C. butyricum CGMCC0313.1	-5.079052	11.62647	-27.86652	17.70841	-32.87343	22.71533
LR+PF+X vs C. butyricum CGMCC0313.1	-4.659201	10.79495	-25.81692	16.49851	-30.88566	21.56726
BAL+LA+LR+X vs C. butyricum CGMCC0313.1	7.687942	9.343767	-10.6255	26.00139	-15.87168	31.24756
LA+LR+ST+BL+BBR+X vs C. butyricum CGMCC0313.1	-1.572257	9.239695	-19.68173	16.53721	-24.94466	21.80015
VSL#3 vs C. butyricum CGMCC0313.1	-6.878104	10.19287	-26.85576	13.09955	-31.98625	18.23004
LP+LC+BL+ST+LA+X vs C. butyricum CGMCC0313.1	-0.8517308	8.729425	-17.96109	16.25763	-23.31533	21.61187
Placebo vs C. butyricum CGMCC0313.1	6.049694	7.883226	-9.401146	21.50053	-14.94432	27.04371
B. infantis 35624 vs L. gasseri BNR17	43.49871	16.26449	11.6209	75.37653	6.617624	80.37981
L. casei LCR35 vs L. gasseri BNR17	34.89912	17.09902	1.385662	68.41258	-3.658713	73.45695
S. boulardii CNCM I-475 vs L. gasseri BNR17	39.959	15.49803	9.583431	70.33457	4.60912	75.30888
LA+LA vs L. gasseri BNR17	29.89946	16.07258	-1.602225	61.40115	-6.597414	66.39633
BL+LR vs L. gasseri BNR17	19.09952	17.00318	-14.22611	52.42515	-19.2653	57.46434
LPA+LS+LP vs L. gasseri BNR17	16.09997	16.63869	-16.51125	48.7112	-21.5318	53.73174
LPA+LA+BAL vs L. gasseri BNR17	36.35946	15.6033	5.777556	66.94136	0.7998074	71.91911
BAL+ST+LB vs L. gasseri BNR17	35.51079	14.96794	6.174163	64.84741	1.214351	69.80723
LP+LP+PA vs L. gasseri BNR17	29.10801	15.75423	-1.769718	59.98574	-6.752705	64.96873
EF+LA+X vs L. gasseri BNR17	24.73155	15.43137	-5.513373	54.97647	-10.4856	59.9487
LA+ST+X vs L. gasseri BNR17	27.95358	14.98978	-1.42586	57.33301	-6.386174	62.29332
BAL+LA+BB+X vs L. gasseri BNR17	24.96951	16.83686	-8.030119	57.96915	-13.06058	62.99961
LR+PF+X vs L. gasseri BNR17	25.38936	16.27378	-6.506658	57.28538	-11.51034	62.28906
BAL+LA+LR+X vs L. gasseri BNR17	37.7365	15.34962	7.651806	67.82121	2.682034	72.79097
LA+LR+ST+BL+BBR+X vs L. gasseri BNR17	28.47631	15.2865	-1.484674	58.43729	-6.452626	63.40524
VSL#3 vs L. gasseri BNR17	23.17046	15.88079	-7.955316	54.29623	-12.94297	59.28389
LP+LC+BL+ST+LA+X vs L. gasseri BNR17	29.19683	14.98358	-0.1704459	58.56411	-5.130616	63.52428
Placebo vs L. gasseri BNR17	36.09826	14.5069	7.665257	64.53127	2.713991	69.48253
L. casei LCR35 vs B. infantis 35624	-8.599591	11.66232	-31.45732	14.25814	-36.46212	19.26294
S. boulardii CNCM I-475 vs B. infantis 35624	-3.53971	9.155445	-21.48405	14.40463	-26.76101	19.68159
LA+LA vs B. infantis 35624	-13.59925	10.09759	-33.39016	6.191654	-38.53188	11.33337
BL+LR vs B. infantis 35624	-24.39919	11.52135	-46.98063	-1.817752	-51.994	3.195614
LPA+LS+LP vs B. infantis 35624	-27.39874	10.9763	-48.9119	-5.885587	-53.96496	-0.832518
LPA+LA+BAL vs B. infantis 35624	-7.139255	9.332532	-25.43068	11.15217	-30.67864	16.40013
BAL+ST+LB vs B. infantis 35624	-7.987924	8.226333	-24.11124	8.135393	-29.5717	13.59586
LP+LP+PA vs B. infantis 35624	-14.3907	9.582756	-33.17255	4.391157	-38.38247	9.601073
EF+LA+X vs B. infantis 35624	-18.76716	9.042134	-36.48942	-1.044905	-41.78587	4.251544
LA+ST+X vs B. infantis 35624	-15.54514	8.266009	-31.74622	0.6559425	-37.19767	6.1074
BAL+LA+BB+X vs B. infantis 35624	-18.5292	11.27444	-40.62671	3.5683	-45.65672	8.59832
LR+PF+X vs B. infantis 35624	-18.10935	10.41486	-38.52209	2.303391	-43.62801	7.409309
BAL+LA+LR+X vs B. infantis 35624	-5.762208	8.90192	-23.20965	11.68524	-28.53127	17.00686
LA+LR+ST+BL+BBR+X vs B. infantis 35624	-15.02241	8.792623	-32.25563	2.210819	-37.59771	7.552896
VSL#3 vs B. infantis 35624	-20.32825	9.789422	-39.51517	-1.141338	-44.69614	4.03963
LP+LC+BL+ST+LA+X vs B. infantis 35624	-14.30188	8.254761	-30.48092	1.877155	-35.93491	7.331154
Placebo vs B. infantis 35624	-7.400455	7.354148	-21.81432	7.01341	-27.50328	12.70237
S. boulardii CNCM I-475 vs L. casei LCR35	5.059882	10.56716	-15.65138	25.77114	-20.74168	30.86144
LA+LA vs L. casei LCR35	-4.999661	11.39316	-27.32984	17.33051	-32.35158	22.35226
BL+LR vs L. casei LCR35	-15.7996	12.67219	-40.63663	9.037433	-45.59834	13.99914
LPA+LS+LP vs L. casei LCR35	-18.79915	12.17875	-42.66906	5.070761	-47.64799	10.0497
LPA+LA+BAL vs L. casei LCR35	1.460337	10.72096	-19.55235	22.47303	-24.62787	27.54854
BAL+ST+LB vs L. casei LCR35	0.6116678	9.773193	-18.54344	19.76678	-23.7266	24.94994
LP+LP+PA vs L. casei LCR35	-5.791108	10.93947	-27.23207	15.64986	-32.28822	20.70601
EF+LA+X vs L. casei LCR35	-10.16757	10.46914	-30.68672	10.35157	-35.78695	15.45181
LA+ST+X vs L. casei LCR35	-6.945546	9.806613	-26.16615	12.27506	-31.34481	17.45372
BAL+LA+BB+X vs L. casei LCR35	-9.929609	12.44812	-34.32748	14.46827	-39.29616	19.43694
LR+PF+X vs L. casei LCR35	-9.509759	11.67527	-32.39287	13.37335	-37.39692	18.3774
BAL+LA+LR+X vs L. casei LCR35	2.837384	10.34828	-17.44487	23.11964	-22.55793	28.2327
LA+LR+ST+BL+BBR+X vs L. casei LCR35	-6.422814	10.25441	-26.52109	13.67546	-31.64455	18.79892
VSL#3 vs L. casei LCR35	-11.72866	11.12095	-33.52533	10.068	-38.5668	15.10947
LP+LC+BL+ST+LA+X vs L. casei LCR35	-5.702288	9.797134	-24.90432	13.49974	-30.08425	18.67967
Placebo vs L. casei LCR35	1.199137	9.051311	-16.54111	18.93938	-21.83595	24.23422
LA+LA vs S. boulardii CNCM I-475	-10.05954	8.810014	-27.32685	7.207767	-32.66563	12.54654
BL+LR vs S. boulardii CNCM I-475	-20.85948	10.41138	-41.2654	-0.45356	-46.37169	4.652727
LPA+LS+LP vs S. boulardii CNCM I-475	-23.85903	9.804809	-43.0761	-4.641958	-48.255	0.5369409
LPA+LA+BAL vs S. boulardii CNCM I-475	-3.599545	7.927698	-19.13755	11.93846	-24.6695	17.47041
BAL+ST+LB vs S. boulardii CNCM I-475	-4.448214	6.583079	-17.35081	8.454384	-23.29764	14.40122
LP+LP+PA vs S. boulardii CNCM I-475	-10.85099	8.21479	-26.95168	5.249705	-32.41479	10.71281
EF+LA+X vs S. boulardii CNCM I-475	-15.22745	7.592223	-30.10794	-0.3469682	-35.72841	5.273508
LA+ST+X vs S. boulardii CNCM I-475	-12.00543	6.633633	-25.00711	0.9962536	-30.9352	6.92435
BAL+LA+BB+X vs S. boulardii CNCM I-475	-14.98949	10.13747	-34.85856	4.879577	-39.99553	10.01655
LR+PF+X vs S. boulardii CNCM I-475	-14.56964	9.171932	-32.5463	3.407016	-37.82048	8.681195
BAL+LA+LR+X vs S. boulardii CNCM I-475	-2.222497	7.403476	-16.73304	12.28805	-22.40741	17.96241
LA+LR+ST+BL+BBR+X vs S. boulardii CNCM I-475	-11.4827	7.28147	-25.75411	2.788723	-31.46496	8.499573
VSL#3 vs S. boulardii CNCM I-475	-16.78854	8.456127	-33.36225	-0.2148388	-38.77208	5.194988
LP+LC+BL+ST+LA+X vs S. boulardii CNCM I-475	-10.76217	6.61309	-23.72359	2.199247	-29.65926	8.134924
Placebo vs S. boulardii CNCM I-475	-3.860745	5.453324	-14.54906	6.827573	-20.99327	13.27178
BL+LR vs LA+LA	-10.79994	11.24882	-32.84721	11.24734	-37.87909	16.27921
LPA+LS+LP vs LA+LA	-13.79949	10.68988	-34.75126	7.152284	-39.82969	12.23071
LPA+LA+BAL vs LA+LA	6.459997	8.993921	-11.16776	24.08776	-16.47274	29.39273
BAL+ST+LB vs LA+LA	5.611329	7.84008	-9.754946	20.9776	-15.30915	26.53181
LP+LP+PA vs LA+LA	-0.7914469	9.253296	-18.92757	17.34468	-24.18829	22.60539
EF+LA+X vs LA+LA	-5.16791	8.692232	-22.20437	11.86855	-27.5659	17.23007
LA+ST+X vs LA+LA	-1.945885	7.881702	-17.39374	13.50197	-22.9373	19.04553
BAL+LA+BB+X vs LA+LA	-4.929948	10.99579	-26.48129	16.6214	-31.53275	21.67286
LR+PF+X vs LA+LA	-4.510098	10.11254	-24.33031	15.31012	-29.47024	20.45005
BAL+LA+LR+X vs LA+LA	7.837045	8.546244	-8.913285	24.58738	-14.30424	29.97833
LA+LR+ST+BL+BBR+X vs LA+LA	-1.423154	8.432354	-17.95026	15.10396	-23.36516	20.51885
VSL#3 vs LA+LA	-6.729001	9.467158	-25.28429	11.82629	-30.51136	17.05336
LP+LC+BL+ST+LA+X vs LA+LA	-0.7026275	7.869894	-16.12734	14.72208	-21.67391	20.26865
Placebo vs LA+LA	6.198798	6.919376	-7.36293	19.76052	-13.19013	25.58772
LPA+LS+LP vs BL+LR	-2.99955	12.04383	-26.60502	20.60592	-31.58991	25.59081
LPA+LA+BAL vs BL+LR	17.25994	10.56744	-3.451873	37.97174	-8.542144	43.06202
BAL+ST+LB vs BL+LR	16.41127	9.60454	-2.413285	35.23582	-7.620047	40.44258
LP+LP+PA vs BL+LR	10.00849	10.78906	-11.13768	31.15466	-16.20695	36.22393
EF+LA+X vs BL+LR	5.632028	10.31189	-14.5789	25.84296	-19.69594	30.96
LA+ST+X vs BL+LR	8.854053	9.638544	-10.03715	27.74525	-15.23903	32.94714
BAL+LA+BB+X vs BL+LR	5.86999	12.31615	-18.26923	30.00921	-23.24267	34.98265
LR+PF+X vs BL+LR	6.28984	11.53446	-16.31729	28.89697	-21.32983	33.90951
BAL+LA+LR+X vs BL+LR	18.63698	10.18915	-1.333375	38.60734	-6.464297	43.73827
LA+LR+ST+BL+BBR+X vs BL+LR	9.376785	10.0938	-10.40671	29.16028	-15.54888	34.30245
VSL#3 vs BL+LR	4.070938	10.97303	-17.43581	25.57768	-22.48915	30.63102
LP+LC+BL+ST+LA+X vs BL+LR	10.09731	9.628895	-8.774977	28.9696	-13.97824	34.17286
Placebo vs BL+LR	16.99874	8.86894	-0.3840675	34.38154	-5.711786	39.70926
LPA+LA+BAL vs LPA+LS+LP	20.25949	9.970384	0.717892	39.80108	-4.439476	44.95845
BAL+ST+LB vs LPA+LS+LP	19.41082	8.943421	1.882033	36.9396	-3.432016	42.25365
LP+LP+PA vs LPA+LS+LP	13.00804	10.20497	-6.993332	33.00941	-12.12243	38.13851
EF+LA+X vs LPA+LS+LP	8.631578	9.699116	-10.37834	27.6415	-15.57169	32.83485
LA+ST+X vs LPA+LS+LP	11.8536	8.979931	-5.746737	29.45394	-11.05421	34.76142
BAL+LA+BB+X vs LPA+LS+LP	8.86954	11.80785	-14.27342	32.0125	-19.27007	37.00915
LR+PF+X vs LPA+LS+LP	9.289391	10.99006	-12.25073	30.82951	-17.30266	35.88144
BAL+LA+LR+X vs LPA+LS+LP	21.63653	9.568501	2.882618	40.39045	-2.329376	45.60244
LA+LR+ST+BL+BBR+X vs LPA+LS+LP	12.37634	9.466918	-6.178483	30.93115	-11.40559	36.15826
VSL#3 vs LPA+LS+LP	7.070487	10.39928	-13.31173	27.4527	-18.4193	32.56027
LP+LC+BL+ST+LA+X vs LPA+LS+LP	13.09686	8.969568	-4.48317	30.67689	-9.7925	35.98622
Placebo vs LPA+LS+LP	19.99829	8.148355	4.027803	35.96877	-1.450694	41.44727
BAL+ST+LB vs LPA+LA+BAL	-0.8486688	6.825359	-14.22613	12.52879	-20.0856	18.38826
LP+LP+PA vs LPA+LA+BAL	-7.251444	8.411971	-23.73861	9.235717	-29.15788	14.65499
EF+LA+X vs LPA+LA+BAL	-11.62791	7.764592	-26.84623	3.590413	-32.42009	9.16428
LA+ST+X vs LPA+LA+BAL	-8.405883	6.87135	-21.87348	5.061717	-27.71706	10.90529
BAL+LA+BB+X vs LPA+LA+BAL	-11.38995	10.29769	-31.57304	8.793147	-36.69165	13.91176
LR+PF+X vs LPA+LA+BAL	-10.9701	9.348717	-29.29324	7.353052	-34.53864	12.59844
BAL+LA+LR+X vs LPA+LA+BAL	1.377047	7.637331	-13.59185	16.34594	-19.19989	21.95399
LA+LR+ST+BL+BBR+X vs LPA+LA+BAL	-7.883151	7.492877	-22.56892	6.802619	-28.21738	12.45108
VSL#3 vs LPA+LA+BAL	-13.189	8.644649	-30.1322	3.754201	-35.50316	9.125165
LP+LC+BL+ST+LA+X vs LPA+LA+BAL	-7.162625	6.868934	-20.62549	6.300237	-26.46989	12.14464
Placebo vs LPA+LA+BAL	-0.2611998	5.745657	-11.52248	11.00008	-17.82243	17.30003
LP+LP+PA vs BAL+ST+LB	-6.402775	7.164849	-20.44562	7.64007	-26.1926	13.38705
EF+LA+X vs BAL+ST+LB	-10.77924	6.420898	-23.36397	1.805491	-29.37278	7.8143
LA+ST+X vs BAL+ST+LB	-7.557214	5.275286	-17.89659	2.782157	-24.43459	9.320158
BAL+LA+BB+X vs BAL+ST+LB	-10.54128	9.306913	-28.78249	7.699939	-34.03453	12.95198
LR+PF+X vs BAL+ST+LB	-10.12143	8.244682	-26.28071	6.037855	-31.73699	11.49414
BAL+LA+LR+X vs BAL+ST+LB	2.225716	6.22611	-9.977235	14.42867	-16.06439	20.51582
LA+LR+ST+BL+BBR+X vs BAL+ST+LB	-7.034482	6.066854	-18.9253	4.856332	-25.07981	11.01084
VSL#3 vs BAL+ST+LB	-12.34033	7.438772	-26.92006	2.239397	-32.58411	7.903452
LP+LC+BL+ST+LA+X vs BAL+ST+LB	-6.313956	5.259015	-16.62144	3.993524	-23.16824	10.54033
Placebo vs BAL+ST+LB	0.587469	3.686334	-6.637613	7.812551	-14.25012	15.42506
EF+LA+X vs LP+LP+PA	-4.376463	8.088827	-20.23027	11.47735	-25.72283	16.96991
LA+ST+X vs LP+LP+PA	-1.154438	7.210396	-15.28655	12.97768	-21.01927	18.7104
BAL+LA+BB+X vs LP+LP+PA	-4.138502	10.52498	-24.76709	16.49009	-29.86162	21.58461
LR+PF+X vs LP+LP+PA	-3.718651	9.598512	-22.53139	15.09409	-27.73902	20.30172
BAL+LA+LR+X vs LP+LP+PA	8.628491	7.931191	-6.916358	24.17334	-12.44744	29.70442
LA+LR+ST+BL+BBR+X vs LP+LP+PA	-0.6317067	7.808589	-15.93626	14.67285	-21.49862	20.2352
VSL#3 vs LP+LP+PA	-5.937554	8.916027	-23.41265	11.53754	-28.73168	16.85658
LP+LC+BL+ST+LA+X vs LP+LP+PA	0.0888194	7.197321	-14.01767	14.19531	-19.75447	19.9321
Placebo vs LP+LP+PA	6.990244	6.143755	-5.051294	19.03178	-11.1729	25.15339
LA+ST+X vs EF+LA+X	3.222025	6.467249	-9.453549	15.8976	-15.44435	21.8884
BAL+LA+BB+X vs EF+LA+X	0.2379615	10.03526	-19.43079	19.90671	-24.58008	25.05601
LR+PF+X vs EF+LA+X	0.6578121	9.058846	-17.0972	18.41282	-22.39073	23.70635
BAL+LA+LR+X vs EF+LA+X	13.00495	7.29385	-1.290729	27.30064	-6.997816	33.00772
LA+LR+ST+BL+BBR+X vs EF+LA+X	3.744756	7.118915	-10.20806	17.69757	-15.96961	23.45913
VSL#3 vs EF+LA+X	-1.561091	8.327741	-17.88316	14.76098	-23.32083	20.19864
LP+LC+BL+ST+LA+X vs EF+LA+X	4.465282	6.480405	-8.236078	17.16664	-14.22181	23.15238
Placebo vs EF+LA+X	11.36671	5.260846	1.05564	21.67778	-5.490173	28.22359
BAL+LA+BB+X vs LA+ST+X	-2.984063	9.342003	-21.29405	15.32592	-26.54051	20.57238
LR+PF+X vs LA+ST+X	-2.564213	8.284272	-18.80109	13.67266	-24.24844	19.12001
BAL+LA+LR+X vs LA+ST+X	9.78293	6.2802	-2.526036	22.0919	-8.590994	28.15685
LA+LR+ST+BL+BBR+X vs LA+ST+X	0.5227317	6.119426	-11.47112	12.51659	-17.60306	18.64852
VSL#3 vs LA+ST+X	-4.783116	7.482321	-19.44819	9.881964	-25.09968	15.53345
LP+LC+BL+ST+LA+X vs LA+ST+X	1.243258	5.322361	-9.188377	11.67489	-15.70113	18.18764
Placebo vs LA+ST+X	8.144683	3.774043	0.7476953	15.54167	-6.792748	23.08211
LR+PF+X vs BAL+LA+BB+X	0.4198505	11.28784	-21.70391	22.54361	-26.73297	27.57267
BAL+LA+LR+X vs BAL+LA+BB+X	12.76699	9.909096	-6.654477	32.18847	-11.81966	37.35365
LA+LR+ST+BL+BBR+X vs BAL+LA+BB+X	3.506795	9.811032	-15.72247	22.73606	-20.90054	27.91413
VSL#3 vs BAL+LA+BB+X	-1.799052	10.71349	-22.79711	19.199	-27.87332	24.27521
LP+LC+BL+ST+LA+X vs BAL+LA+BB+X	4.227321	9.332047	-14.06316	22.5178	-19.31119	27.76583
Placebo vs BAL+LA+BB+X	11.12875	8.545734	-5.620585	27.87808	-11.01165	33.26914
BAL+LA+LR+X vs LR+PF+X	12.34714	8.918873	-5.133527	29.82781	-10.45204	35.14633
LA+LR+ST+BL+BBR+X vs LR+PF+X	3.086944	8.809796	-14.17994	20.35383	-19.51875	25.69264
VSL#3 vs LR+PF+X	-2.218903	9.804848	-21.43605	16.99825	-26.61494	22.17714
LP+LC+BL+ST+LA+X vs LR+PF+X	3.807471	8.273043	-12.40739	20.02234	-17.85727	25.47221
Placebo vs LR+PF+X	10.7089	7.374667	-3.745186	25.16298	-9.428047	30.84584
LA+LR+ST+BL+BBR+X vs BAL+LA+LR+X	-9.260199	6.962368	-22.90619	4.385791	-28.71892	10.19852
VSL#3 vs BAL+LA+LR+X	-14.56605	8.181625	-30.60174	1.469645	-36.07249	6.940394
LP+LC+BL+ST+LA+X vs BAL+LA+LR+X	-8.539672	6.254343	-20.79796	3.718615	-26.87348	9.79414
Placebo vs BAL+LA+LR+X	-1.638247	5.016051	-11.46953	8.193032	-18.15264	14.87615
VSL#3 vs LA+LR+ST+BL+BBR+X	-5.305847	8.059657	-21.10249	10.49079	-26.60203	15.99034
LP+LC+BL+ST+LA+X vs LA+LR+ST+BL+BBR+X	0.7205261	6.111227	-11.25726	12.69831	-17.39269	18.83375
Placebo vs LA+LR+ST+BL+BBR+X	7.621951	4.819407	-1.823912	17.06781	-8.624235	23.86814
LP+LC+BL+ST+LA+X vs VSL#3	6.026373	7.471799	-8.618084	20.67083	-14.27259	26.32534
Placebo vs VSL#3	12.9278	6.461368	0.2637493	25.59185	-5.729323	31.58492
Placebo vs LP+LC+BL+ST+LA+X	6.901425	3.749353	-0.4471722	14.25002	-8.007732	21.81058

5. Abdominal bloating score

_Comparison	_Effect_Size	_Standard_Error	_LCI	_UCI	_LPrI	_UPrI
S. cerevisiae CNCM I-3856 vs L. plantarum CCFM8610	11.58197	12.87969	-13.66176	36.8257	-18.95997	42.12391
L. plantarum Apsulloc 331261 vs L. plantarum CCFM8610	-2.777841	15.45191	-33.06304	27.50735	-38.63649	33.08081
L. plantarum 299v vs L. plantarum CCFM8610	5.131821	10.57274	-15.59037	25.85401	-20.76633	31.02998
B. coagulans Unique IS2 vs L. plantarum CCFM8610	11.92249	10.64223	-8.935904	32.78088	-14.11318	37.95816
B. coagulans MTCC 5856 vs L. plantarum CCFM8610	-10.74722	20.65421	-51.22873	29.73429	-57.60634	36.1119
L. acidophilus DDS-1 vs L. plantarum CCFM8610	8.192414	10.33052	-12.05503	28.43986	-17.22779	33.61261
L. acidophilus ATCC 700396 vs L. plantarum CCFM8610	18.82244	9.889589	-0.5607986	38.20568	-5.733539	43.37842
B. lactis UABla-12 vs L. plantarum CCFM8610	13.49241	10.37179	-6.835926	33.82076	-12.00907	38.9939
B. bifidum MIMBb75 vs L. plantarum CCFM8610	8.091559	9.511966	-10.55155	26.73467	-15.73079	31.91391
C. butyricum CGMCC0313.1 vs L. plantarum CCFM8610	18.96595	9.559554	0.229566	37.70233	-4.9485	42.8804
B. infantis 35624 vs L. plantarum CCFM8610	21.32166	10.26661	1.199478	41.44385	-3.972796	46.61612
S. boulardii CNCM I-475 vs L. plantarum CCFM8610	18.85467	9.534605	0.1671892	37.54215	-5.011477	42.72082
BL+LR vs L. plantarum CCFM8610	-14.077	13.98071	-41.47869	13.32469	-46.8806	18.7266
LPA+LA+BAL vs L. plantarum CCFM8610	25.58876	9.395717	7.173496	44.00403	1.990959	49.18657
BAL+ST+LB vs L. plantarum CCFM8610	19.00844	8.458537	2.430012	35.58687	-2.80435	40.82123
EF+LA+X vs L. plantarum CCFM8610	15.32227	9.978371	-4.234977	34.87952	-9.407086	40.05162
LA+ST+X vs L. plantarum CCFM8610	10.62181	9.666297	-8.323789	29.5674	-13.49961	34.74322
BAL+LA+BB+X vs L. plantarum CCFM8610	11.92195	11.73258	-11.07349	34.91739	-16.29224	40.13614
LR+PF+X vs L. plantarum CCFM8610	9.212239	10.49215	-11.352	29.77648	-16.52665	34.95113
BAL+LA+LR+X vs L. plantarum CCFM8610	18.65435	9.12072	0.7780698	36.53064	-4.414873	41.72358
LA+LR+ST+BL+BBR+X vs L. plantarum CCFM8610	16.94491	8.900897	-0.5005294	34.39035	-5.704551	39.59437
VSL#3 vs L. plantarum CCFM8610	6.213778	8.766213	-10.96768	23.39524	-16.17977	28.60733
LP+LC+BL+ST+LA+X vs L. plantarum CCFM8610	14.21126	8.427327	-2.305998	30.72852	-7.542931	35.96545
Placebo vs L. plantarum CCFM8610	19.92294	7.645381	4.938269	34.90761	-0.3837384	40.22962
L. plantarum Apsulloc 331261 vs S. cerevisiae CNCM I-3856	-14.35981	16.96388	-47.60841	18.88879	-53.38892	24.6693
L. plantarum 299v vs S. cerevisiae CNCM I-3856	-6.450147	12.68002	-31.30254	18.40224	-36.58464	23.68435
B. coagulans Unique IS2 vs S. cerevisiae CNCM I-3856	0.3405215	12.73775	-24.62502	25.30606	-29.91169	30.59273
B. coagulans MTCC 5856 vs S. cerevisiae CNCM I-3856	-22.32919	21.80811	-65.0723	20.41393	-71.65602	26.99765
L. acidophilus DDS-1 vs S. cerevisiae CNCM I-3856	-3.389554	12.47854	-27.84705	21.06794	-33.11382	26.33471
L. acidophilus ATCC 700396 vs S. cerevisiae CNCM I-3856	7.240473	12.11598	-16.50641	30.98735	-21.74805	36.229
B. lactis UABla-12 vs S. cerevisiae CNCM I-3856	1.910446	12.51273	-22.61406	26.43495	-27.88336	31.70425
B. bifidum MIMBb75 vs S. cerevisiae CNCM I-3856	-3.490409	11.81012	-26.63781	19.657	-31.86087	24.88006
C. butyricum CGMCC0313.1 vs S. cerevisiae CNCM I-3856	7.38398	11.84825	-15.83816	30.60612	-21.0634	35.83136
B. infantis 35624 vs S. cerevisiae CNCM I-3856	9.739695	12.4258	-14.61442	34.09381	-19.87733	39.35672
S. boulardii CNCM I-475 vs S. cerevisiae CNCM I-3856	7.272704	11.82818	-15.91011	30.45552	-21.1342	35.67961
BL+LR vs S. cerevisiae CNCM I-3856	-25.65897	15.63538	-56.30375	4.985808	-61.90081	10.58288
LPA+LA+BAL vs S. cerevisiae CNCM I-3856	14.00679	11.71627	-8.956675	36.97026	-14.17454	42.18813
BAL+ST+LB vs S. cerevisiae CNCM I-3856	7.426473	10.97909	-14.09214	28.94509	-19.27819	34.13113
EF+LA+X vs S. cerevisiae CNCM I-3856	3.740302	12.18862	-20.14895	27.62956	-25.39537	32.87597
LA+ST+X vs S. cerevisiae CNCM I-3856	-0.9601628	11.93465	-24.35165	22.43132	-29.58197	27.66165
BAL+LA+BB+X vs S. cerevisiae CNCM I-3856	0.3399803	13.66202	-26.43709	27.11705	-31.80656	32.48652
LR+PF+X vs S. cerevisiae CNCM I-3856	-2.369729	12.61272	-27.09021	22.35075	-32.36709	27.62763
BAL+LA+LR+X vs S. cerevisiae CNCM I-3856	7.072383	11.4977	-15.4627	29.60746	-20.66943	34.8142
LA+LR+ST+BL+BBR+X vs S. cerevisiae CNCM I-3856	5.362941	11.32339	-16.8305	27.55638	-22.02935	32.75523
VSL#3 vs S. cerevisiae CNCM I-3856	-5.36819	11.21833	-27.35572	16.61934	-32.55027	21.81389
LP+LC+BL+ST+LA+X vs S. cerevisiae CNCM I-3856	2.629291	10.95678	-18.84561	24.10419	-24.03095	29.28953
Placebo vs S. cerevisiae CNCM I-3856	8.340972	10.36541	-11.97487	28.65681	-17.14795	33.82989
L. plantarum 299v vs L. plantarum Apsulloc 331261	7.909662	15.28636	-22.05105	37.87037	-27.60357	43.4229
B. coagulans Unique IS2 vs L. plantarum Apsulloc 331261	14.70033	15.33428	-15.3543	44.75496	-20.91284	50.31351
B. coagulans MTCC 5856 vs L. plantarum Apsulloc 331261	-7.969377	23.41972	-53.87119	37.93243	-60.75421	44.81546
L. acidophilus DDS-1 vs L. plantarum Apsulloc 331261	10.97025	15.11965	-18.66371	40.60422	-24.19555	46.13606
L. acidophilus ATCC 700396 vs L. plantarum Apsulloc 331261	21.60028	14.82183	-7.449969	50.65054	-12.94587	56.14643
B. lactis UABla-12 vs L. plantarum Apsulloc 331261	16.27026	15.14788	-13.41904	45.95955	-18.95436	51.49487
B. bifidum MIMBb75 vs L. plantarum Apsulloc 331261	10.8694	14.57287	-17.6929	39.4317	-23.15978	44.89859
C. butyricum CGMCC0313.1 vs L. plantarum Apsulloc 331261	21.74379	14.60379	-6.879112	50.36669	-12.34955	55.83713
B. infantis 35624 vs L. plantarum Apsulloc 331261	24.0995	15.07615	-5.449197	53.6482	-10.97571	59.17472
S. boulardii CNCM I-475 vs L. plantarum Apsulloc 331261	21.63251	14.58752	-6.958494	50.22352	-12.42706	55.69209
BL+LR vs L. plantarum Apsulloc 331261	-11.29916	17.81445	-46.21484	23.61653	-52.12285	29.52453
LPA+LA+BAL vs L. plantarum Apsulloc 331261	28.3666	14.49692	-0.0468383	56.78004	-5.505064	62.23827
BAL+ST+LB vs L. plantarum Apsulloc 331261	21.78628	13.90792	-5.472731	49.0453	-10.86707	54.43963
EF+LA+X vs L. plantarum Apsulloc 331261	18.10011	14.88127	-11.06664	47.26686	-16.56961	52.76983
LA+ST+X vs L. plantarum Apsulloc 331261	13.39965	14.67398	-15.36082	42.16011	-20.83937	47.63866
BAL+LA+BB+X vs L. plantarum Apsulloc 331261	14.69979	16.11026	-16.87574	46.27532	-22.53598	51.93555
LR+PF+X vs L. plantarum Apsulloc 331261	11.99008	15.23058	-17.8613	41.84146	-23.40686	47.38702
BAL+LA+LR+X vs L. plantarum Apsulloc 331261	21.43219	14.32085	-6.636161	49.50055	-12.07467	54.93906
LA+LR+ST+BL+BBR+X vs L. plantarum Apsulloc 331261	19.72275	14.18129	-8.072064	47.51756	-13.49532	52.94082
VSL#3 vs L. plantarum Apsulloc 331261	8.991619	14.09754	-18.63906	36.6223	-24.05331	42.03655
LP+LC+BL+ST+LA+X vs L. plantarum Apsulloc 331261	16.9891	13.89031	-10.2354	44.2136	-15.62792	49.60612
Placebo vs L. plantarum Apsulloc 331261	22.70078	13.42876	-3.619097	49.02066	-8.966033	54.3676
B. coagulans Unique IS2 vs L. plantarum 299v	6.790668	10.39951	-13.59199	27.17333	-18.76544	32.34677
B. coagulans MTCC 5856 vs L. plantarum 299v	-15.87904	20.5303	-56.11768	24.3596	-62.47362	30.71554
L. acidophilus DDS-1 vs L. plantarum 299v	3.060593	10.08035	-16.69652	22.81771	-21.86831	27.9895
L. acidophilus ATCC 700396 vs L. plantarum 299v	13.69062	9.627892	-5.179702	32.56094	-10.35627	37.73751
B. lactis UABla-12 vs L. plantarum 299v	8.360593	10.12264	-11.47942	28.2006	-16.65119	33.37238
B. bifidum MIMBb75 vs L. plantarum 299v	2.959738	9.240035	-15.1504	21.06987	-20.33837	26.25784
C. butyricum CGMCC0313.1 vs L. plantarum 299v	13.83413	9.288722	-4.371432	32.03969	-9.557576	37.22583
B. infantis 35624 vs L. plantarum 299v	16.18984	10.01498	-3.439157	35.81884	-8.611102	40.99079
S. boulardii CNCM I-475 vs L. plantarum 299v	13.72285	9.263117	-4.432525	31.87823	-9.619615	37.06532
BL+LR vs L. plantarum 299v	-19.20882	13.79726	-46.25095	7.833313	-51.63396	13.21632
LPA+LA+BAL vs L. plantarum 299v	20.45694	9.119778	2.582505	38.33138	-2.61048	43.52436
BAL+ST+LB vs L. plantarum 299v	13.87662	8.151057	-2.099159	29.8524	-7.361487	35.11473
EF+LA+X vs L. plantarum 299v	10.19045	9.719148	-8.858731	29.23963	-14.03362	34.41452
LA+ST+X vs L. plantarum 299v	5.489985	9.398684	-12.9311	23.91107	-18.11354	29.09351
BAL+LA+BB+X vs L. plantarum 299v	6.790128	11.51305	-15.77503	29.35528	-20.98249	34.56275
LR+PF+X vs L. plantarum 299v	4.080419	10.24598	-16.00134	24.16217	-21.17349	29.33433
BAL+LA+LR+X vs L. plantarum 299v	13.52253	8.83723	-3.798121	30.84318	-9.005832	36.05089
LA+LR+ST+BL+BBR+X vs L. plantarum 299v	11.81309	8.609214	-5.06066	28.68684	-10.28343	33.9096
VSL#3 vs L. plantarum 299v	1.081957	8.47056	-15.52003	17.68395	-20.75342	22.91734
LP+LC+BL+ST+LA+X vs L. plantarum 299v	9.079438	8.121006	-6.837442	24.99632	-12.10283	30.2617
Placebo vs L. plantarum 299v	14.79112	7.303505	0.4765126	29.10573	-4.896527	34.47877
B. coagulans MTCC 5856 vs B. coagulans Unique IS2	-22.66971	20.566	-62.97833	17.63892	-69.34051	24.00109
L. acidophilus DDS-1 vs B. coagulans Unique IS2	-3.730076	10.15287	-23.62933	16.16918	-28.80115	21.341
L. acidophilus ATCC 700396 vs B. coagulans Unique IS2	6.899951	9.703796	-12.11914	25.91904	-17.29429	31.09419
B. lactis UABla-12 vs B. coagulans Unique IS2	1.569924	10.19486	-18.41164	21.55149	-23.58356	26.72341
B. bifidum MIMBb75 vs B. coagulans Unique IS2	-3.83093	9.319098	-22.09603	14.43417	-27.28109	19.61923
C. butyricum CGMCC0313.1 vs B. coagulans Unique IS2	7.043458	9.367374	-11.31626	25.40318	-16.4997	30.58661
B. infantis 35624 vs B. coagulans Unique IS2	9.399173	10.08797	-10.37289	29.17123	-15.54467	34.34301
S. boulardii CNCM I-475 vs B. coagulans Unique IS2	6.932182	9.341985	-11.37777	25.24214	-16.56205	30.42642
BL+LR vs B. coagulans Unique IS2	-25.99949	13.85033	-53.14565	1.146667	-58.53406	6.535081
LPA+LA+BAL vs B. coagulans Unique IS2	13.66627	9.199877	-4.365154	31.6977	-9.554718	36.88726
BAL+ST+LB vs B. coagulans Unique IS2	7.085951	8.240577	-9.065283	23.23719	-14.31885	28.49076
EF+LA+X vs B. coagulans Unique IS2	3.39978	9.794345	-15.79678	22.59634	-20.97057	27.77013
LA+ST+X vs B. coagulans Unique IS2	-1.300684	9.476424	-19.87413	17.27277	-25.05431	22.45294
BAL+LA+BB+X vs B. coagulans Unique IS2	-0.0005412	11.5766	-22.69025	22.68917	-27.90084	27.89976
LR+PF+X vs B. coagulans Unique IS2	-2.71025	10.31734	-22.93187	17.51137	-28.10451	22.68401
BAL+LA+LR+X vs B. coagulans Unique IS2	6.731862	8.919862	-10.75074	24.21447	-15.95371	29.41743
LA+LR+ST+BL+BBR+X vs B. coagulans Unique IS2	5.022419	8.694017	-12.01754	22.06238	-17.23437	27.27921
VSL#3 vs B. coagulans Unique IS2	-5.708712	8.556737	-22.47961	11.06218	-27.70626	16.28884
LP+LC+BL+ST+LA+X vs B. coagulans Unique IS2	2.288769	8.210842	-13.80419	18.38172	-19.06061	23.63815
Placebo vs B. coagulans Unique IS2	8.000451	7.403279	-6.50971	22.51061	-11.86691	27.86781
L. acidophilus DDS-1 vs B. coagulans MTCC 5856	18.93963	20.40647	-21.05632	58.93559	-27.39069	65.26996
L. acidophilus ATCC 700396 vs B. coagulans MTCC 5856	29.56966	20.1868	-9.995749	69.13507	-16.29211	75.43143
B. lactis UABla-12 vs B. coagulans MTCC 5856	24.23963	20.4274	-15.79733	64.2766	-22.13535	70.61461
B. bifidum MIMBb75 vs B. coagulans MTCC 5856	18.83878	20.00472	-20.36976	58.04731	-26.63485	64.3124
C. butyricum CGMCC0313.1 vs B. coagulans MTCC 5856	29.71317	20.02726	-9.539537	68.96587	-15.80848	75.23482
B. infantis 35624 vs B. coagulans MTCC 5856	32.06888	20.37426	-7.863937	72.0017	-14.19272	78.33048
S. boulardii CNCM I-475 vs B. coagulans MTCC 5856	29.60189	20.01539	-9.627563	68.83134	-15.89448	75.09826
BL+LR vs B. coagulans MTCC 5856	-3.329781	22.4761	-47.38213	40.72257	-54.08841	47.42885
LPA+LA+BAL vs B. coagulans MTCC 5856	36.33598	19.94946	-2.764253	75.43621	-9.019892	81.69185
BAL+ST+LB vs B. coagulans MTCC 5856	29.75566	19.52564	-8.513887	68.02521	-14.69776	74.20908
EF+LA+X vs B. coagulans MTCC 5856	26.06949	20.23049	-13.58154	65.72051	-19.88543	72.02441
LA+ST+X vs B. coagulans MTCC 5856	21.36902	20.0785	-17.9841	60.72215	-24.26184	66.99989
BAL+LA+BB+X vs B. coagulans MTCC 5856	22.66917	21.15091	-18.78585	64.12418	-25.25126	70.58959
LR+PF+X vs B. coagulans MTCC 5856	19.95946	20.4888	-20.19785	60.11676	-26.54655	66.46547
BAL+LA+LR+X vs B. coagulans MTCC 5856	29.40157	19.82189	-9.448615	68.25175	-15.68252	74.48566
LA+LR+ST+BL+BBR+X vs B. coagulans MTCC 5856	27.69213	19.72129	-10.96089	66.34515	-17.17774	72.562
VSL#3 vs B. coagulans MTCC 5856	16.961	19.66116	-21.57417	55.49616	-27.78085	61.70284
LP+LC+BL+ST+LA+X vs B. coagulans MTCC 5856	24.95848	19.5131	-13.28649	63.20345	-19.46826	69.38522
Placebo vs B. coagulans MTCC 5856	30.67016	19.18728	-6.93623	68.27655	-13.06372	74.40404
L. acidophilus ATCC 700396 vs L. acidophilus DDS-1	10.63003	9.360947	-7.717092	28.97715	-12.90074	34.16079
B. lactis UABla-12 vs L. acidophilus DDS-1	5.3	7.079198	-8.574972	19.17497	-13.98665	24.58665
B. bifidum MIMBb75 vs L. acidophilus DDS-1	-0.1008547	8.961544	-17.66516	17.46345	-22.86587	22.66416
C. butyricum CGMCC0313.1 vs L. acidophilus DDS-1	10.77353	9.011735	-6.889142	28.43621	-12.08726	33.63433
B. infantis 35624 vs L. acidophilus DDS-1	13.12925	9.758629	-5.997312	32.25581	-11.17159	37.43009
S. boulardii CNCM I-475 vs L. acidophilus DDS-1	10.66226	8.98534	-6.948686	28.2732	-12.14815	33.47267
BL+LR vs L. acidophilus DDS-1	-22.26941	13.61233	-48.94908	4.410256	-54.31366	9.774838
LPA+LA+BAL vs L. acidophilus DDS-1	17.39635	8.837499	0.0751675	34.71753	-5.132527	39.92522
BAL+ST+LB vs L. acidophilus DDS-1	10.81603	7.83395	-4.538233	26.17029	-9.835885	31.46794
EF+LA+X vs L. acidophilus DDS-1	7.129856	9.45478	-11.40117	25.66088	-16.58195	30.84166
LA+ST+X vs L. acidophilus DDS-1	2.429391	9.125035	-15.45535	20.31413	-20.6481	25.50688
BAL+LA+BB+X vs L. acidophilus DDS-1	3.729535	11.29076	-18.39994	25.85901	-23.59742	31.05649
LR+PF+X vs L. acidophilus DDS-1	1.019826	9.995558	-18.57111	20.61076	-23.74313	25.78278
BAL+LA+LR+X vs L. acidophilus DDS-1	10.46194	8.545618	-6.287165	27.21104	-11.51466	32.43854
LA+LR+ST+BL+BBR+X vs L. acidophilus DDS-1	8.752495	8.309609	-7.53404	25.03903	-12.78121	30.2862
VSL#3 vs L. acidophilus DDS-1	-1.978636	8.165868	-17.98344	14.02617	-23.24429	19.28702
LP+LC+BL+ST+LA+X vs L. acidophilus DDS-1	6.018845	7.802664	-9.274096	21.31178	-14.57561	26.6133
Placebo vs L. acidophilus DDS-1	11.73053	6.94782	-1.88695	25.348	-7.323281	30.78433
B. lactis UABla-12 vs L. acidophilus ATCC 700396	-5.330027	9.406475	-23.76638	13.10633	-28.94859	18.28853
B. bifidum MIMBb75 vs L. acidophilus ATCC 700396	-10.73088	8.449389	-27.29138	5.829617	-32.52649	11.06473
C. butyricum CGMCC0313.1 vs L. acidophilus ATCC 700396	0.1435072	8.502604	-16.52129	16.80831	-21.75212	22.03914
B. infantis 35624 vs L. acidophilus ATCC 700396	2.499222	9.29052	-15.70986	20.70831	-20.89594	25.89438
S. boulardii CNCM I-475 vs L. acidophilus ATCC 700396	0.0322307	8.474625	-16.57773	16.64219	-21.81079	21.87525
S. boulardii CNCM I-475 vs L. acidophilus ATCC 700396	0.0322307	8.474625	-16.57773	16.64219	-21.81079	21.87525
BL+LR vs L. acidophilus ATCC 700396	-32.89944	13.28075	-58.92923	-6.869648	-64.26242	-1.536457
LPA+LA+BAL vs L. acidophilus ATCC 700396	6.766321	8.317709	-9.53609	23.06873	-14.78253	28.31517
BAL+ST+LB vs L. acidophilus ATCC 700396	0.1859999	7.242491	-14.00902	14.38102	-19.39215	19.76415
EF+LA+X vs L. acidophilus ATCC 700396	-3.500171	8.970829	-21.08267	14.08233	-26.28289	19.28255
LA+ST+X vs L. acidophilus ATCC 700396	-8.200636	8.622597	-25.10062	8.699344	-30.32242	13.92115
BAL+LA+BB+X vs L. acidophilus ATCC 700396	-6.900493	10.88872	-28.24198	14.441	-33.4253	19.62431
LR+PF+X vs L. acidophilus ATCC 700396	-9.610202	9.539083	-28.30646	9.086057	-33.48502	14.26461
BAL+LA+LR+X vs L. acidophilus ATCC 700396	-0.1680893	8.006905	-15.86133	15.52516	-21.13887	20.80269
LA+LR+ST+BL+BBR+X vs L. acidophilus ATCC 700396	-1.877532	7.754516	-17.0761	13.32104	-22.3837	18.62864
VSL#3 vs L. acidophilus ATCC 700396	-12.60866	7.600284	-27.50495	2.287619	-32.83317	7.615841
LP+LC+BL+ST+LA+X vs L. acidophilus ATCC 700396	-4.611182	7.208644	-18.73987	9.517501	-24.12873	14.90636
Placebo vs L. acidophilus ATCC 700396	1.1005	6.273366	-11.19507	13.39607	-16.78386	18.98486
B. bifidum MIMBb75 vs B. lactis UABla-12	-5.400855	9.009091	-23.05835	12.25664	-28.2566	17.45489
C. butyricum CGMCC0313.1 vs B. lactis UABla-12	5.473534	9.059019	-12.28182	23.22888	-17.47761	28.42468
B. infantis 35624 vs B. lactis UABla-12	7.829248	9.802311	-11.38293	27.04142	-16.55661	32.21511
S. boulardii CNCM I-475 vs B. lactis UABla-12	5.362257	9.032763	-12.34163	23.06615	-17.5387	28.26322
BL+LR vs B. lactis UABla-12	-27.56941	13.64367	-54.31053	-0.8283022	-59.67819	4.539359
LPA+LA+BAL vs B. lactis UABla-12	12.09635	8.88571	-5.319324	29.51202	-10.52421	34.7169
BAL+ST+LB vs B. lactis UABla-12	5.516027	7.888297	-9.944751	20.9768	-15.23586	26.26791
EF+LA+X vs B. lactis UABla-12	1.829856	9.499859	-16.78953	20.44924	-21.96908	25.62879
LA+ST+X vs B. lactis UABla-12	-2.870609	9.171735	-20.84688	15.10566	-26.03761	20.29639
BAL+LA+BB+X vs B. lactis UABla-12	-1.570466	11.32853	-23.77398	20.63305	-28.97305	25.83212
LR+PF+X vs B. lactis UABla-12	-4.280174	10.03821	-23.9547	15.39435	-29.12657	20.56622
BAL+LA+LR+X vs B. lactis UABla-12	5.161938	8.595467	-11.68487	22.00874	-16.90864	27.23251
LA+LR+ST+BL+BBR+X vs B. lactis UABla-12	3.452495	8.360865	-12.9345	19.83949	-18.1771	25.08209
VSL#3 vs B. lactis UABla-12	-7.278636	8.21802	-23.38566	8.828388	-28.64139	14.08412
LP+LC+BL+ST+LA+X vs B. lactis UABla-12	0.7188447	7.857228	-14.68104	16.11873	-19.97586	21.41355
Placebo vs B. lactis UABla-12	6.430526	7.009041	-7.306943	20.168	-12.7316	25.59265
C. butyricum CGMCC0313.1 vs B. bifidum MIMBb75	10.87439	8.060782	-4.924454	26.67323	-10.19615	31.94492
B. infantis 35624 vs B. bifidum MIMBb75	13.2301	8.887952	-4.189962	30.65017	-9.394716	35.85492
S. boulardii CNCM I-475 vs B. bifidum MIMBb75	10.76311	8.031266	-4.977881	26.5041	-10.25275	31.77897
BL+LR vs B. bifidum MIMBb75	-22.16856	13.00232	-47.65263	3.315517	-52.96118	8.624063
LPA+LA+BAL vs B. bifidum MIMBb75	17.4972	7.865503	2.0811	32.9133	-3.212727	38.20713
BAL+ST+LB vs B. bifidum MIMBb75	10.91688	6.718299	-2.250742	24.08451	-7.733924	29.56769
EF+LA+X vs B. bifidum MIMBb75	7.230711	8.553227	-9.533308	23.99473	-14.76023	29.22165
LA+ST+X vs B. bifidum MIMBb75	2.530246	8.187254	-13.51648	18.57697	-18.7752	23.83569
BAL+LA+BB+X vs B. bifidum MIMBb75	3.830389	10.54733	-16.84199	24.50277	-22.01752	29.67829
LR+PF+X vs B. bifidum MIMBb75	1.12068	9.14746	-16.80801	19.04937	-21.99978	24.24114
BAL+LA+LR+X vs B. bifidum MIMBb75	10.56279	7.536096	-4.207683	25.33327	-9.545014	30.6706
LA+LR+ST+BL+BBR+X vs B. bifidum MIMBb75	8.85335	7.267348	-5.390391	23.09709	-10.76937	28.47607
VSL#3 vs B. bifidum MIMBb75	-1.877781	7.102551	-15.79853	12.04296	-21.20598	17.45042
LP+LC+BL+ST+LA+X vs B. bifidum MIMBb75	6.119699	6.681867	-6.976519	19.21592	-12.4676	24.707
Placebo vs B. bifidum MIMBb75	11.83138	5.660129	0.7377326	22.92503	-5.032755	28.69552
B. infantis 35624 vs C. butyricum CGMCC0313.1	2.355714	8.938557	-15.16353	19.87496	-20.36548	25.07691
S. boulardii CNCM I-475 vs C. butyricum CGMCC0313.1	-0.1112765	8.087232	-15.96196	15.73941	-21.23085	21.0083
BL+LR vs C. butyricum CGMCC0313.1	-33.04295	13.03696	-58.59492	-7.49097	-63.90644	-2.179446
LPA+LA+BAL vs C. butyricum CGMCC0313.1	6.622814	7.922644	-8.905282	22.15091	-14.19236	27.43799
BAL+ST+LB vs C. butyricum CGMCC0313.1	0.0424927	6.785107	-13.25607	13.34106	-18.72511	18.81009
EF+LA+X vs C. butyricum CGMCC0313.1	-3.643678	8.605801	-20.51074	13.22338	-25.73375	18.4464
LA+ST+X vs C. butyricum CGMCC0313.1	-8.344143	8.242161	-24.49848	7.810196	-29.7519	13.06362
BAL+LA+BB+X vs C. butyricum CGMCC0313.1	-7.044	10.59	-27.80003	13.71203	-32.9763	18.8883
LR+PF+X vs C. butyricum CGMCC0313.1	-9.753709	9.196637	-27.77879	8.271369	-32.96848	13.46106
BAL+LA+LR+X vs C. butyricum CGMCC0313.1	-0.3115965	7.595703	-15.1989	14.57571	-20.52776	19.90457
LA+LR+ST+BL+BBR+X vs C. butyricum CGMCC0313.1	-2.021039	7.329154	-16.38592	12.34384	-21.75481	17.71273
VSL#3 vs C. butyricum CGMCC0313.1	-12.75217	7.165776	-26.79683	1.292492	-32.19309	6.688751
LP+LC+BL+ST+LA+X vs C. butyricum CGMCC0313.1	-4.75469	6.749003	-17.98249	8.473114	-23.45912	13.94974
Placebo vs C. butyricum CGMCC0313.1	0.9569923	5.739264	-10.29176	12.20574	-16.03622	17.9502
S. boulardii CNCM I-475 vs B. infantis 35624	-2.466991	8.911946	-19.93409	15.0001	-25.13749	20.20351
BL+LR vs B. infantis 35624	-35.39866	13.56399	-61.9836	-8.813729	-67.34347	-3.453856
LPA+LA+BAL vs B. infantis 35624	4.267099	8.762863	-12.9078	21.442	-18.1201	26.65429
BAL+ST+LB vs B. infantis 35624	-2.313222	7.749656	-17.50227	12.87582	-22.81049	18.18404
EF+LA+X vs B. infantis 35624	-5.999393	9.385056	-24.39377	12.39498	-29.57664	17.57785
LA+ST+X vs B. infantis 35624	-10.69986	9.052773	-28.44297	7.043251	-33.63906	12.23935
BAL+LA+BB+X vs B. infantis 35624	-9.399714	11.23244	-31.41489	12.61546	-36.60999	17.81056
LR+PF+X vs B. infantis 35624	-12.10942	9.929632	-31.57114	7.3523	-36.74356	12.52471
BAL+LA+LR+X vs B. infantis 35624	-2.667311	8.46842	-19.26511	13.93049	-24.49867	19.16405
LA+LR+ST+BL+BBR+X vs B. infantis 35624	-4.376754	8.230187	-20.50763	11.75412	-25.76219	17.00868
VSL#3 vs B. infantis 35624	-15.10788	8.085037	-30.95427	0.7384966	-36.22339	6.007619
LP+LC+BL+ST+LA+X vs B. infantis 35624	-7.110404	7.718057	-22.23752	8.01671	-27.54983	13.32902
Placebo vs B. infantis 35624	-1.398722	6.852637	-14.82964	12.0322	-20.28481	17.48736
BL+LR vs S. boulardii CNCM I-475	-32.93167	13.01873	-58.44791	-7.415429	-63.75787	-2.105475
LPA+LA+BAL vs S. boulardii CNCM I-475	6.73409	7.893067	-8.736037	22.20422	-14.02658	27.49476
BAL+ST+LB vs S. boulardii CNCM I-475	0.1537692	6.750449	-13.07687	13.38441	-18.55319	18.86073
EF+LA+X vs S. boulardii CNCM I-475	-3.532402	8.578158	-20.34528	13.28048	-25.57033	18.50553
LA+ST+X vs S. boulardii CNCM I-475	-8.232866	8.213296	-24.33063	7.864899	-29.58682	13.12108
BAL+LA+BB+X vs S. boulardii CNCM I-475	-6.932723	10.56755	-27.64474	13.7793	-32.82062	18.95517
LR+PF+X vs S. boulardii CNCM I-475	-9.642432	9.170774	-27.61682	8.331956	-32.80759	13.52273
BAL+LA+LR+X vs S. boulardii CNCM I-475	-0.20032	7.562922	-15.02337	14.62273	-20.35686	19.95622
LA+LR+ST+BL+BBR+X vs S. boulardii CNCM I-475	-1.909763	7.297175	-16.21196	12.39244	-21.58604	17.76651
VSL#3 vs S. boulardii CNCM I-475	-12.64089	7.132655	-26.62064	1.338852	-32.02272	6.740934
LP+LC+BL+ST+LA+X vs S. boulardii CNCM I-475	-4.643413	6.70873	-17.79228	8.505456	-23.27752	13.9907
Placebo vs S. boulardii CNCM I-475	1.068269	5.69773	-10.09908	12.23561	-15.85709	17.99363
LPA+LA+BAL vs BL+LR	39.66576	12.91714	14.34864	64.98288	9.047302	70.28422
BAL+ST+LB vs BL+LR	33.08544	12.25242	9.071138	57.09974	3.820424	62.35046
EF+LA+X vs BL+LR	29.39927	13.34705	3.239523	55.55901	-2.09977	60.89831
LA+ST+X vs BL+LR	24.6988	13.11554	-1.007172	50.40478	-6.325544	55.72316
BAL+LA+BB+X vs BL+LR	25.99895	14.70484	-2.822003	54.8199	-8.304148	60.30204
LR+PF+X vs BL+LR	23.28924	13.73544	-3.631721	50.2102	-9.008498	55.58698
BAL+LA+LR+X vs BL+LR	32.73135	12.71922	7.802142	57.66056	2.516947	62.94575
LA+LR+ST+BL+BBR+X vs BL+LR	31.02191	12.56187	6.40109	55.64273	1.128094	60.91572
VSL#3 vs BL+LR	20.29078	12.46725	-4.144591	44.72615	-9.41053	49.99208
LP+LC+BL+ST+LA+X vs BL+LR	28.28826	12.23243	4.313142	52.26337	-0.936214	57.51273
Placebo vs BL+LR	33.99994	11.70569	11.05721	56.94267	5.83991	62.15997
BAL+ST+LB vs LPA+LA+BAL	-6.580321	6.55099	-19.42003	6.259384	-24.94054	11.7799
EF+LA+X vs LPA+LA+BAL	-10.26649	8.423172	-26.7756	6.242621	-32.01289	11.4799
LA+ST+X vs LPA+LA+BAL	-14.96696	8.051284	-30.74718	0.8132704	-36.01989	6.085978
BAL+LA+BB+X vs LPA+LA+BAL	-13.66681	10.44214	-34.13302	6.799396	-39.30699	11.97336
LR+PF+X vs LPA+LA+BAL	-16.37652	9.02597	-34.0671	1.314055	-39.26451	6.511463
BAL+LA+LR+X vs LPA+LA+BAL	-6.93441	7.391218	-21.42093	7.552111	-26.78	12.91118
LA+LR+ST+BL+BBR+X vs LPA+LA+BAL	-8.643853	7.112763	-22.58461	5.296907	-27.99024	10.70253
VSL#3 vs LPA+LA+BAL	-19.37498	6.94517	-32.98727	-5.762702	-38.42411	-0.3258584
LP+LC+BL+ST+LA+X vs LPA+LA+BAL	-11.3775	6.525166	-24.16659	1.411587	-29.69312	6.938117
Placebo vs LPA+LA+BAL	-5.665822	5.461612	-16.37039	5.038742	-22.20981	10.87817
EF+LA+X vs BAL+ST+LB	-3.686171	7.363369	-18.11811	10.74577	-23.48155	16.10921
LA+ST+X vs BAL+ST+LB	-8.386636	6.934881	-21.97875	5.205482	-27.41759	10.64432
BAL+LA+BB+X vs BAL+ST+LB	-7.086493	9.607684	-25.91721	11.74422	-31.09419	16.92121
LR+PF+X vs BAL+ST+LB	-9.796202	8.045964	-25.566	5.973598	-30.83928	11.24688
BAL+LA+LR+X vs BAL+ST+LB	-0.3540892	6.155565	-12.41878	11.7106	-18.03904	17.33086
LA+LR+ST+BL+BBR+X vs BAL+ST+LB	-2.063532	5.819246	-13.46905	9.341981	-19.18801	15.06094
VSL#3 vs BAL+ST+LB	-12.79466	5.612961	-23.79586	-1.793462	-29.58225	3.992928
LP+LC+BL+ST+LA+X vs BAL+ST+LB	-4.797182	5.081454	-14.75665	5.162284	-20.74357	11.1492
Placebo vs BAL+ST+LB	0.9144996	3.619198	-6.178998	8.007998	-12.96552	14.79451
LA+ST+X vs EF+LA+X	-4.700464	8.724375	-21.79992	12.399	-27.01472	17.61379
BAL+LA+BB+X vs EF+LA+X	-3.400321	10.96949	-24.90012	18.09948	-30.08586	23.28522
LR+PF+X vs EF+LA+X	-6.11003	9.63118	-24.9868	12.76674	-30.1633	17.94324
BAL+LA+LR+X vs EF+LA+X	3.332082	8.116406	-12.57578	19.23995	-17.84164	24.50581
LA+LR+ST+BL+BBR+X vs EF+LA+X	1.622639	7.86753	-13.79744	17.04272	-19.09102	22.3363
VSL#3 vs EF+LA+X	-9.108492	7.715559	-24.23071	6.013725	-29.54335	11.32637
LP+LC+BL+ST+LA+X vs EF+LA+X	-1.111011	7.330082	-15.47771	13.25569	-20.84645	18.62443
Placebo vs EF+LA+X	4.600671	6.412539	-7.967674	17.16902	-13.52122	22.72256
BAL+LA+BB+X vs LA+ST+X	1.300143	10.68658	-19.64518	22.24546	-24.82339	27.42367
LR+PF+X vs LA+ST+X	-1.409566	9.307687	-19.6523	16.83316	-24.83776	22.01863
BAL+LA+LR+X vs LA+ST+X	8.032546	7.729789	-7.117563	23.18266	-12.42835	28.49344
LA+LR+ST+BL+BBR+X vs LA+ST+X	6.323103	7.468025	-8.313956	20.96016	-13.66129	26.3075
VSL#3 vs LA+ST+X	-4.408028	7.307752	-18.73096	9.914904	-24.10331	15.28725
LP+LC+BL+ST+LA+X vs LA+ST+X	3.589453	6.89957	-9.933455	17.11236	-15.37923	22.55813
Placebo vs LA+ST+X	9.301135	5.915578	-2.293185	20.89545	-7.982494	26.58476
LR+PF+X vs BAL+LA+BB+X	-2.709709	11.43888	-25.1295	19.71008	-30.33348	24.91406
BAL+LA+LR+X vs BAL+LA+BB+X	6.732403	10.19629	-13.25196	26.71677	-18.42389	31.8887
LA+LR+ST+BL+BBR+X vs BAL+LA+BB+X	5.02296	9.999321	-14.57535	24.62127	-19.74736	29.79328
VSL#3 vs BAL+LA+BB+X	-5.70817	9.880193	-25.07299	13.65665	-30.24582	18.82948
LP+LC+BL+ST+LA+X vs BAL+LA+BB+X	2.28931	9.582191	-16.49144	21.07006	-21.66899	26.2476
Placebo vs BAL+LA+BB+X	8.000992	8.899944	-9.442579	25.44456	-14.64666	30.64864
BAL+LA+LR+X vs LR+PF+X	9.442112	8.740387	-7.688732	26.57296	-12.90248	31.7867
LA+LR+ST+BL+BBR+X vs LR+PF+X	7.732669	8.50978	-8.946194	24.41153	-14.17646	29.6418
VSL#3 vs LR+PF+X	-2.998462	8.369478	-19.40234	13.40541	-24.64419	18.64727
LP+LC+BL+ST+LA+X vs LR+PF+X	4.999019	8.01551	-10.71109	20.70913	-15.98768	25.98572
Placebo vs LR+PF+X	10.7107	7.186026	-3.373651	24.79505	-8.766397	30.1878
LA+LR+ST+BL+BBR+X vs BAL+LA+LR+X	-1.709443	6.751189	-14.94153	11.52264	-20.41769	16.99881
VSL#3 vs BAL+LA+LR+X	-12.44057	6.570662	-25.31884	0.4376884	-30.83482	5.953672
LP+LC+BL+ST+LA+X vs BAL+LA+LR+X	-4.443093	6.078196	-16.35614	7.469952	-21.99793	13.11174
Placebo vs BAL+LA+LR+X	1.268589	4.975468	-8.483149	11.02033	-14.51506	17.05224
VSL#3 vs LA+LR+ST+BL+BBR+X	-10.73113	6.259679	-22.99988	1.537615	-28.59224	7.12998
LP+LC+BL+ST+LA+X vs LA+LR+ST+BL+BBR+X	-2.73365	5.790228	-14.08229	8.614987	-19.81041	14.34311
Placebo vs LA+LR+ST+BL+BBR+X	2.978032	4.558227	-5.955928	11.91199	-12.18289	18.13896
LP+LC+BL+ST+LA+X vs VSL#3	7.997481	5.57191	-2.923262	18.91822	-8.723732	24.71869
Placebo vs VSL#3	13.70916	4.290595	5.299752	22.11857	-1.068563	28.48689
Placebo vs LP+LC+BL+ST+LA+X	5.711682	3.550924	-1.248002	12.67136	-8.082642	19.50601

6. Bowel movement frequency (per week) in IBS-D (a) and IBS-C (b).

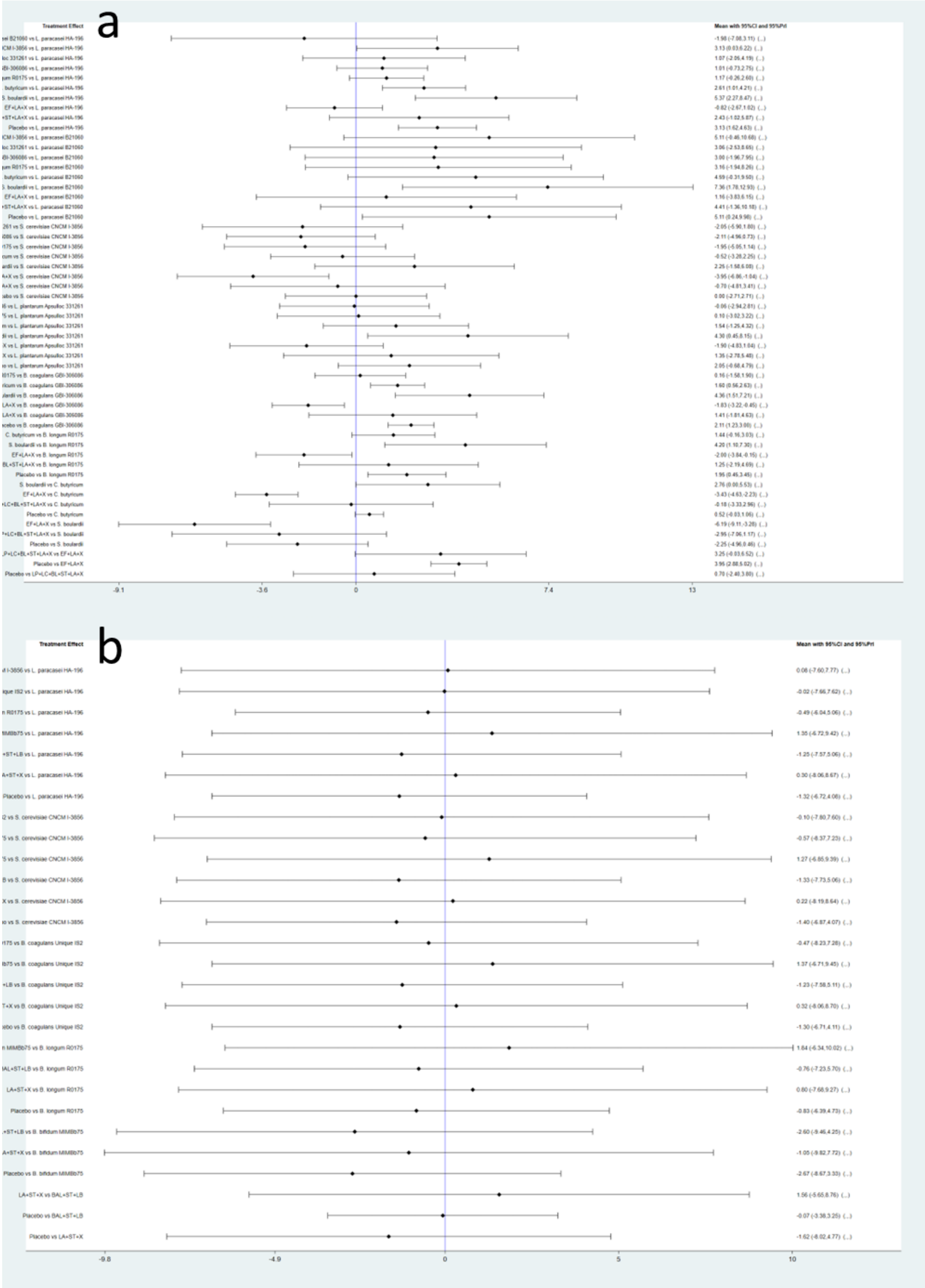

7. Bristol stool form scale in IBS-D (a) and IBS-C (b)

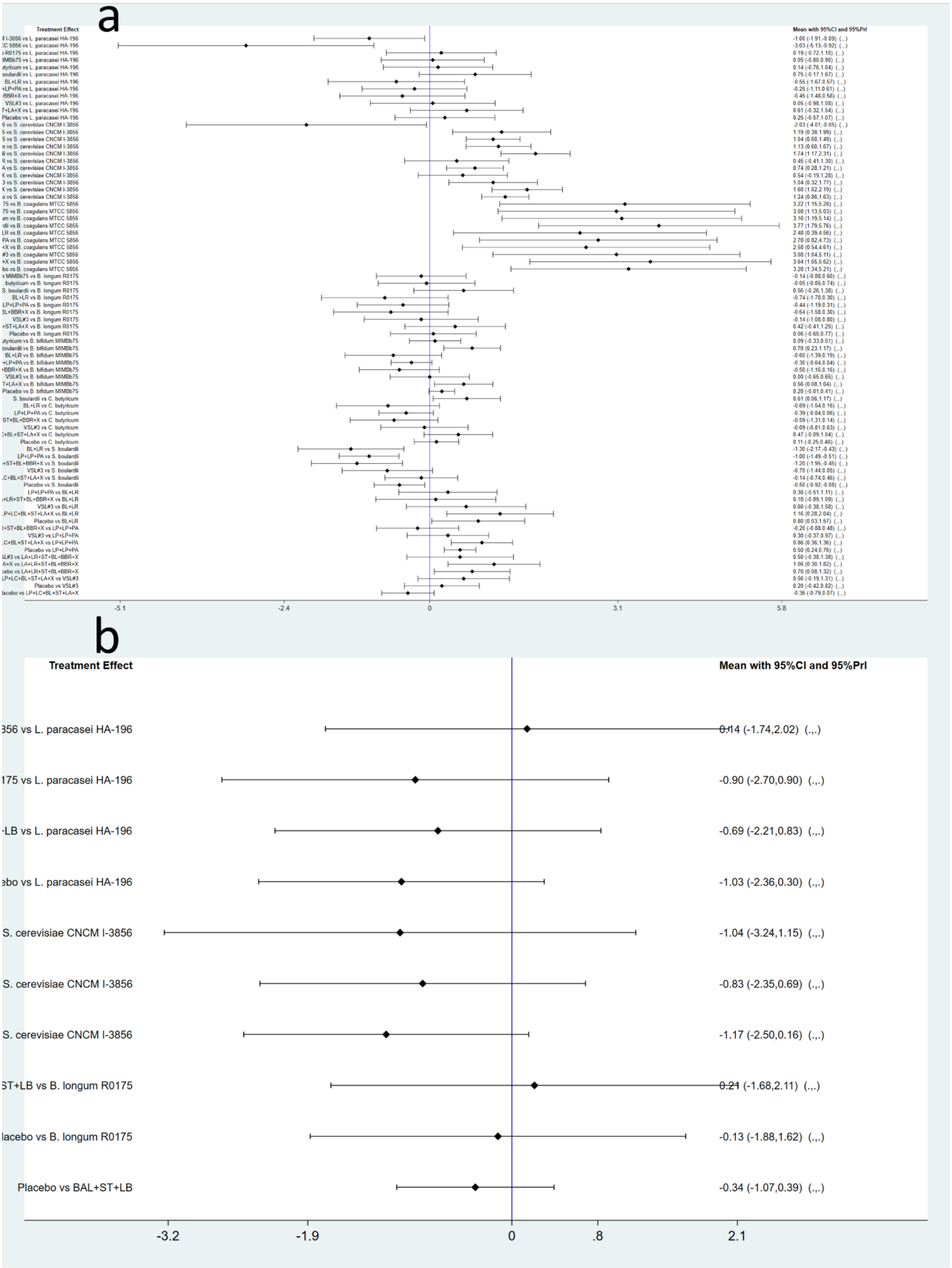

Figure S6. Funnel plots for each outcome

IBS-SSS, IBS Symptom Severity Scale; IBS-QOL, IBS-Quality of Life Measure; HADS, the Hospital Anxiety and Depression Scale.

1. IBS-SSS

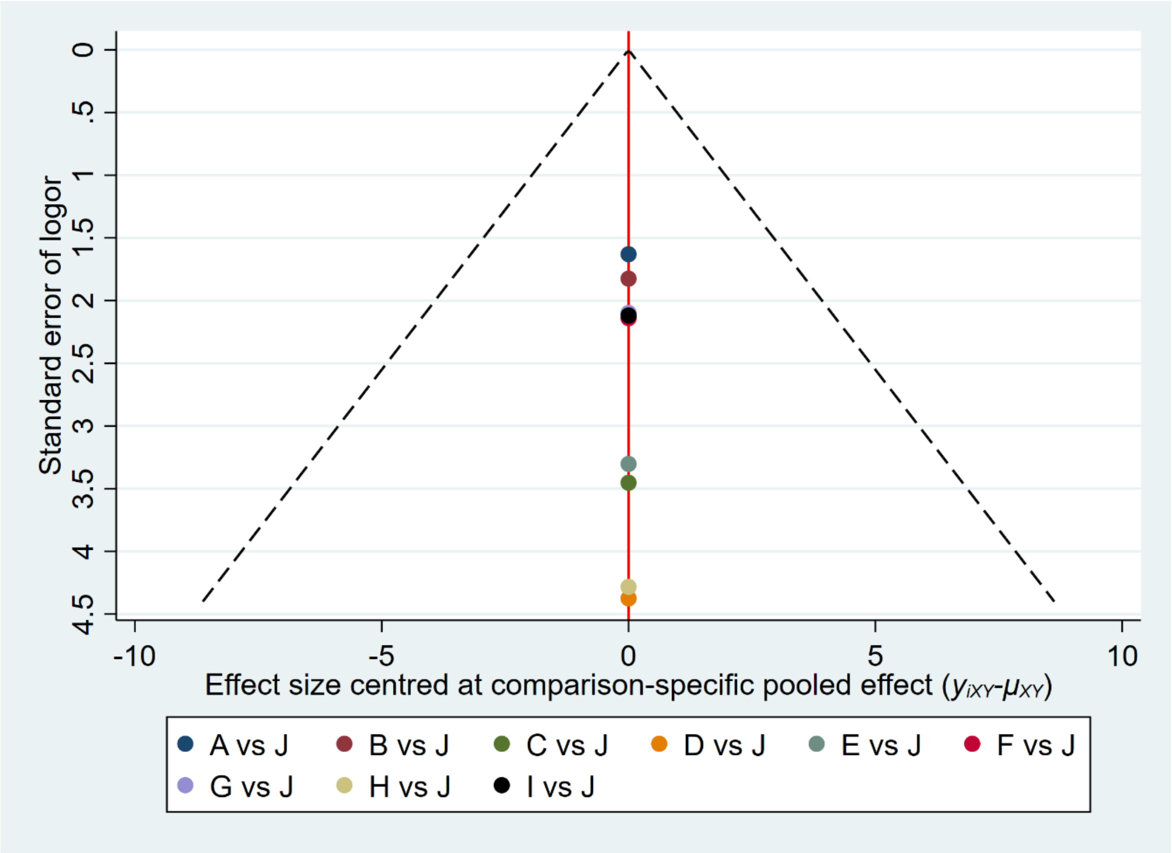

2. IBS-QOL

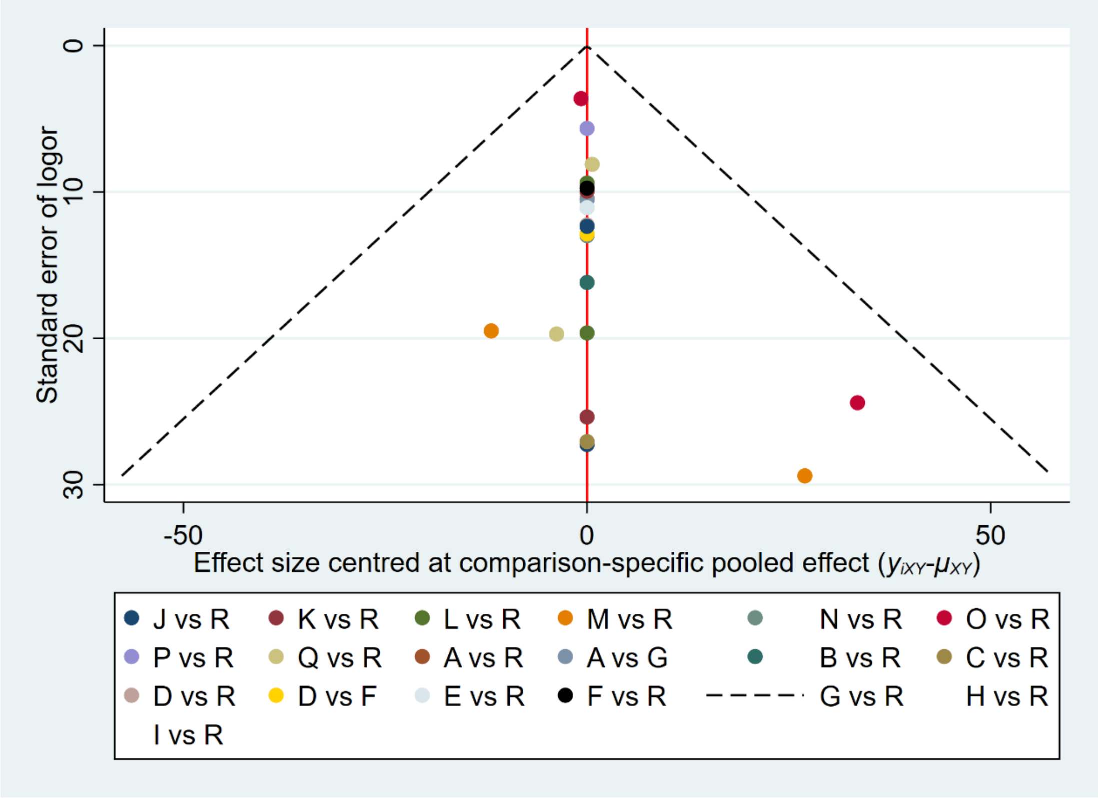

3. **Abdominal pain score**

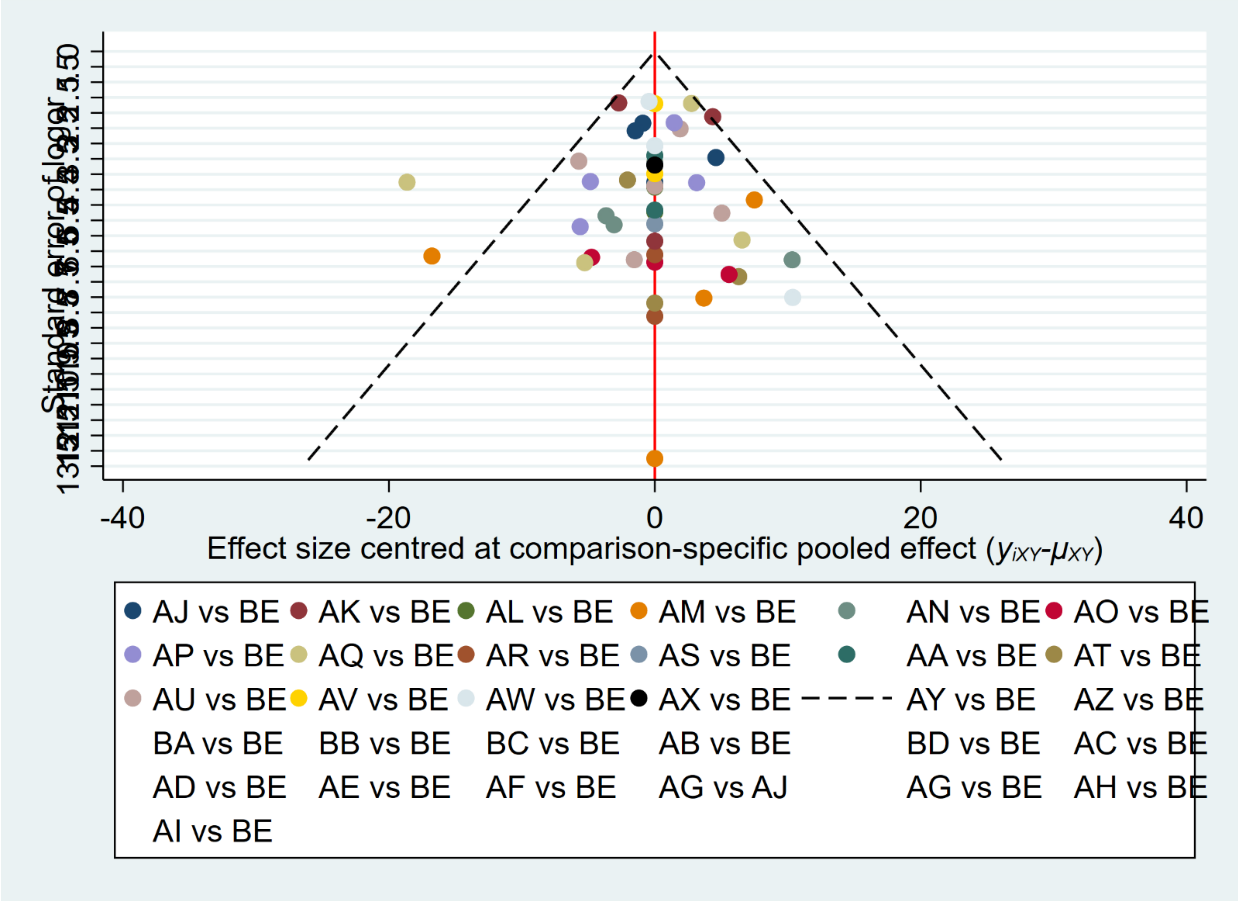

4. **Abdominal bloating score**

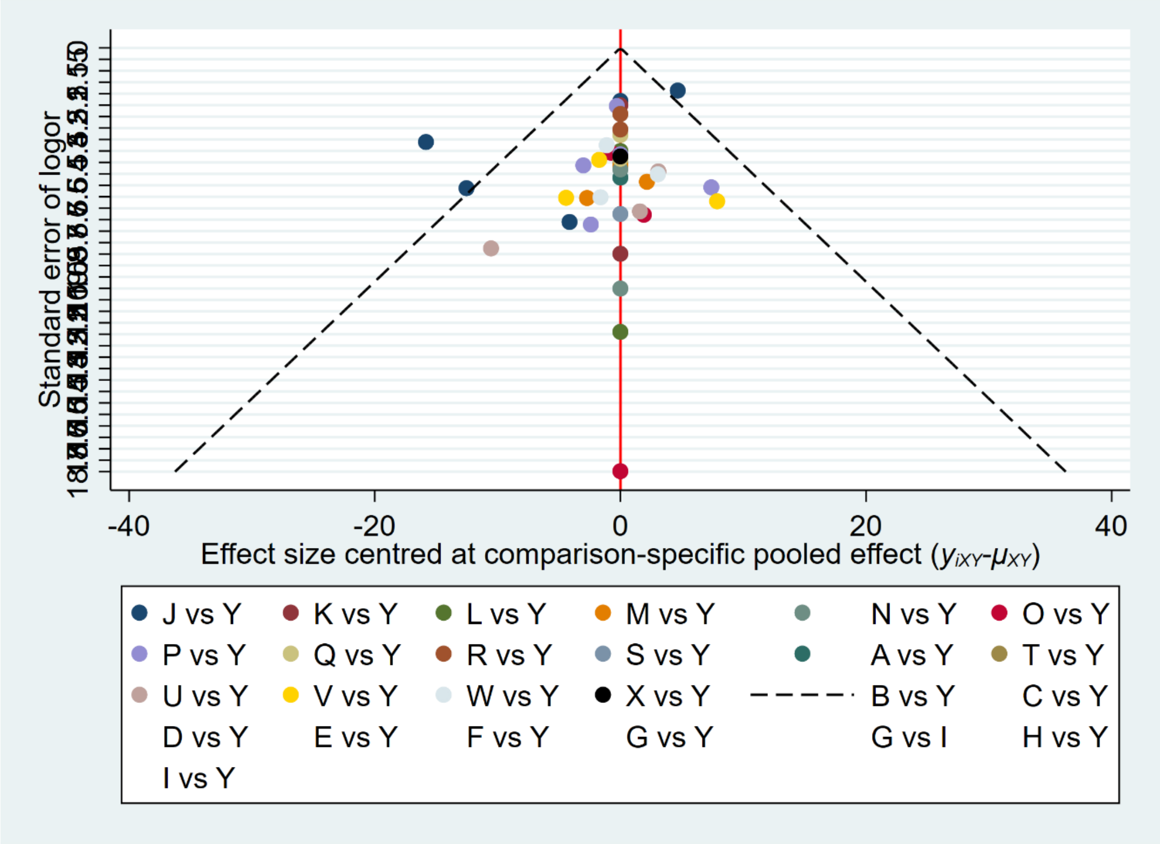

5. **Bowel movement frequency in IBS-D (a) and IBS-C (b)**

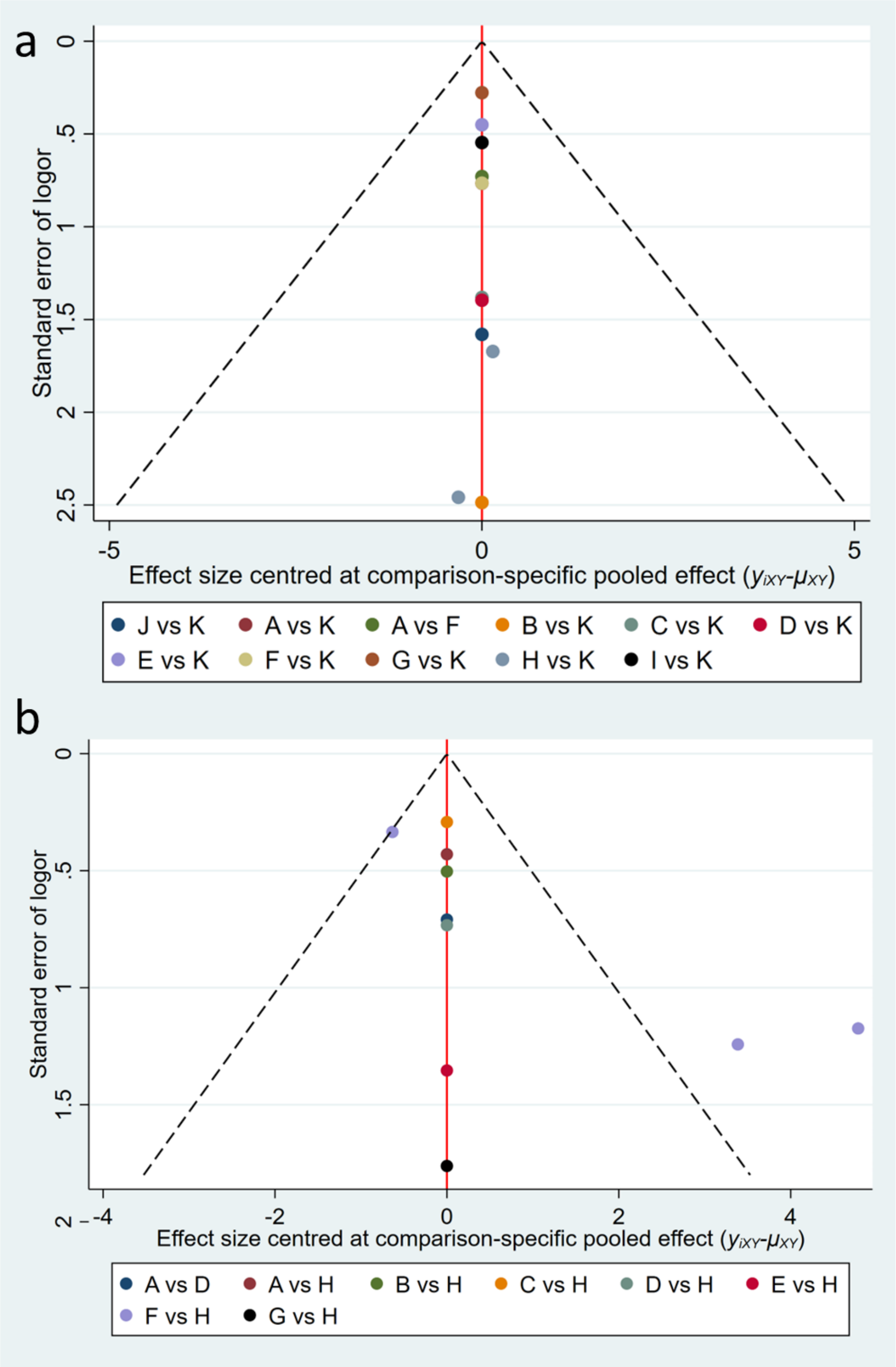

6. Bristol stool form scale IBS-D (a) and IBS-C (b)

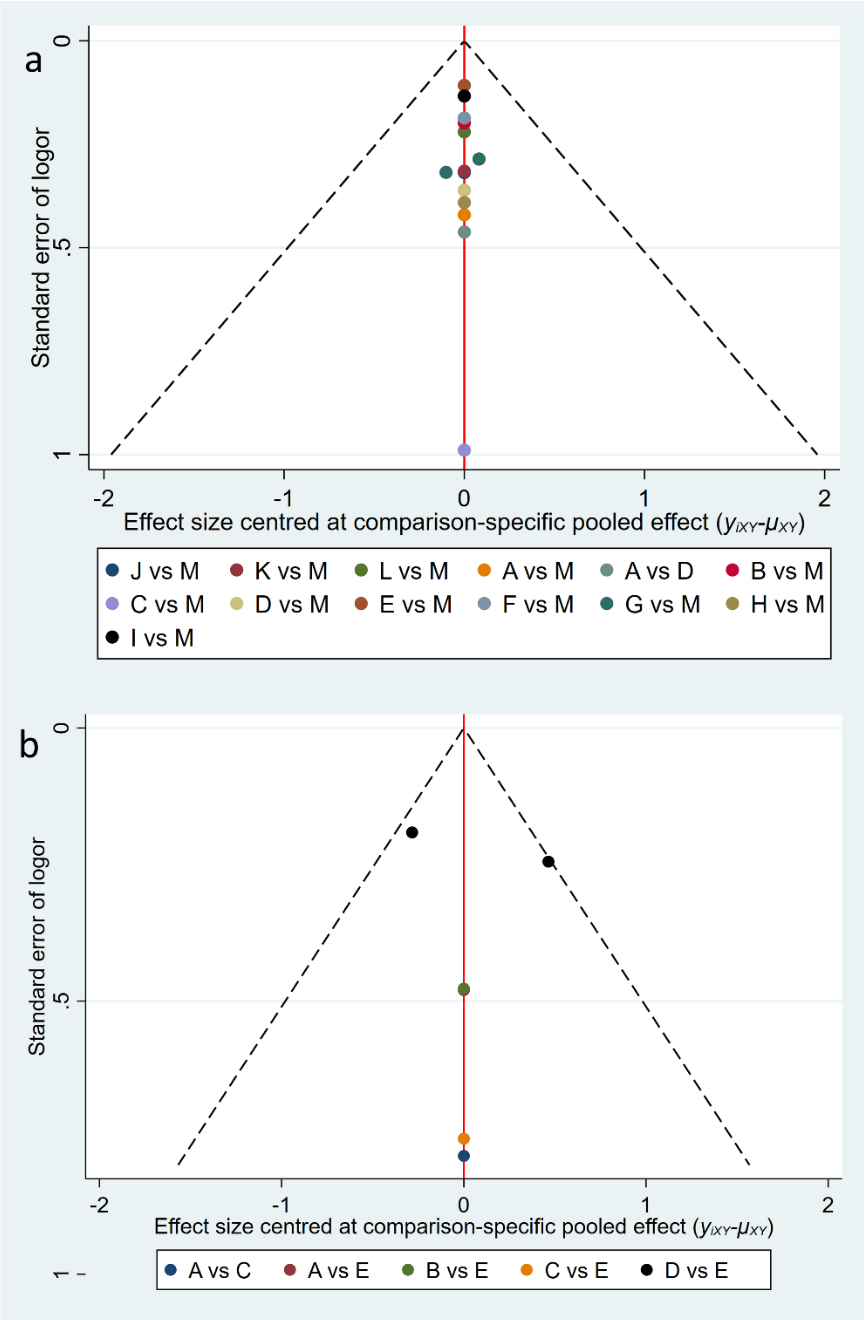

Table S1. Characteristics of Included Randomized Controlled Trials (1).

Study and Publication years	Location	Diagnostic Criteria	IBS Subtype	Intervention time (W)	Probiotic used	Dose (CFU/day)	Sample size (I)	Sample size(C)
Bai, 2022	China	Rome IV	D	4	a multi-strain preparation (4)	$1{\cdot}395 \times 10^{7}$	145	145
Mourey, 2022	France	Rome IV	C	8	S. cerevisiae I-3856	8×10^{9}	230	226
Jung, 2022	Korea	Rome IV	D	4	L. plantarum Apsulloc 331261 (GTB1TM)	1×10^{10}	18	9
Xu, 2021	China	Rome III	All	4	a multi-strain preparation (3)	1×10^{10}	24	24
Skrzydlo-Radomanska, 2021	Poland	Rome III	D	8	a multi-strain preparation (10)	5×10^{9}	25	26
Liu, 2021	China	Rome III	D	8	L. plantarum CCFM8610	1×10^{10}	25	25
Gupta, 2021	India	Rome IV	All	80 days	B. coagulans LBSC (DSM17654)	6×10^{9}	20	20
Barraza-Ortiz, 2021	Mexico	Rome IV	D, M	6	a multi-strain preparation (3)	3×10^{9}	18	18
Sadrin, 2020	France	Rome III	All	8	a multi-strain preparation (2)	1×10^{10}	40	40
Martoni, 2020	India	Rome IV	All	6	L. acidophilus DDS-1	1×10^{10}	111	109
					B. lactis UABla-12	1×10^{10}	110	
Lewis, 2020	Canada	Rome III	All	8	L. paracasei HA-196	1×10^{10}	95	95
					B. longum R0175	1×10^{10}	95	
Kim, 2020	Korea	Rome II	D	8	a multi-strain preparation (5)	$1{\cdot}5 \times 10^{10}$	32	31
Gayathri, 2020	India	Rome III	All	8	S. cerevisiae CNCM I-3856	4×10^{9}	52	48
Bonfrate, 2020	Italy	Rome IV	All	4	a multi-strain preparation (2)	5×10^{9}	15	15
Andresen, 2020	Germany	Rome III	All	8	B. bifidum MIMBb75 (SYN-HI-001)	1×10^{9} bacteria/day	221	222
Oh, 2019	Korea	Rome III	D, M, U	4	a multi-strain preparation (3)	1×10^{9}	28	27
Madempudi, 2019	India	Rome III	C	8	B. coagulans Unique IS2	2×10^{9}	68	68
Ashtiani, 2019	Iran	Rome II	All	4	a multi-strain preparation (7)	2×10^{9}	30	30
Sun, 2018	China	Rome III	D	4	C. butyricum	$5{\cdot}67 \times 10^{7}$	105	95
Shin, 2018	Korea	Rome III	D	8	L. gasseri BNR17	1×10^{10}	30	30
Preston, 2018	USA	Rome III	All	12	a multi-strain preparation (3)	1×10^{11}	76	37
Kim, 2018	Korea	subjective symptoms of IBS	All	4	L. gasseri BNR17	1×10^{10}	10	12
Ishaque, 2018	Bangladesh	Rome III	D	16	a multi-strain preparation (14)	8×10^{9}	181	179
Cremon, 2018	Italy	Rome III	All	8	L. paracasei CNCM I-1572	$4{\cdot}8 \times 10^{10}$ bacteria/day	20	20
Pinto-Sanchez, 2017	Canada	Rome III	D, M	6	B. longum NCC3001	1×10^{10}	18	20
Nobutani, 2017	Japan	Rome III	All	4	L. gasseri CP2305	$1{\cdot}3 \times 10^{8}$	17	17
Hod, 2017	Israel	Rome III	D	8	a multi-strain preparation (11)	$2{\cdot}5 \times 10^{10}$	54	53
Thijssen, 2016	Netherlands	Rome II	All	8	L. casei Shirota	$1{\cdot}3 \times 10^{10}$	39	41
Spiller, 2016	France	Rome III	All	12	S. cerevisiae CNCM I-3856	8×10^{9}	192	187
Mezzasalma, 2016	Italy	Rome III	C	60 days	a multi-strain preparation (2)	4×10^{9}	53	52
					a multi-strain preparation (3)	6×10^{9}	52	
Majeed, 2016	India	Rome III	D	90 days	B. coagulans MTCC 5856	$2 \times 10^{\wedge 9}$	18	18

						spore/day		
Lyra, 2016	Finland	Rome III	All	12	L. acidophilus NCFM (ATCC 700396)	1×10^9	129	131
Yoon, 2015	Korea	Rome III	All	4	a multi-strain preparation (6)	1×10^{10} bacteria/day	39	42
Wong, 2015	Singapore	Rome III	All	6	a multi-strain preparation (8)	9×10^{11} bacteria/day	20	22
de Chambrun, 2015	France	Rome III	All	8	S. cerevisiae CNCM I-3856	4×10^9	93	86
Yoon, 2014	Korea	Rome III	All	4	a multi-strain preparation (6)	1×10^{10} viable cells	25	24
Stevenson, 2014	South Africa	Rome II	All	8	L. plantarum 299V	10×10^9	54	27
Sisson, 2014	UK	Rome III	All	12	a multi-strain preparation (4)	2×10^8/(KG*day)	124	62
Shavakhi, 2014	Iran	Rome III	All	2	a multi-strain preparation (7)	2×10^8	66	63
Ludidi, 2014	Netherlands	Rome III	All	6	a multi-strain preparation (6)	5×10^9	21	19
Lorenzo-Zuniga, 2014	Spain	Rome III	D	6	a multi-strain preparation (3)	$3\text{-}6 \times 10^9$	55	29
Jafari, 2014	Iran	Rome III	All	4	a multi-strain preparation (4)	8×10^9	54	54
Abbas, 2014	Pakistan	Rome III	D	6	S. boulardii	NA	37	35
Roberts, 2013	UK	Rome III	C, M	12	a multi-strain preparation (4)	$2{\cdot}74 \times 10^{10}$	88	91
Lee, 2013	Korea	Rome III	All	6	a multi-strain preparation (5)	$2{\cdot}6 \times 10^{10}$	46	39
Charbonneau, 2013	Ireland	Rome II	All	8	B. infantis 35624	1×10^9	39	37
Cappello, 2013	Italy	Rome III	All	4	a multi-strain preparation (9)	$3{\cdot}8 \times 10^{10}$	34	34
Begtrup, 2013	Denmark	Rome III	All	24	a multi-strain preparation (3)	$5{\cdot}2 \times 10^{10}$	67	64
Amirimani, 2013	Iran	Rome III	All	4	L. reuteri	1×10^8	41	31
Kruis, 2012	Germany	Rome II	All	12	E. coli Nissle 1917	$5\text{-}50 \times 10^9$	60	60
Ducrotte, 2012	India	Rome III	All	4	L. plantarum 299v (DSM 9843)	1×10^{10}	108	106
Dapoigny, 2012	France	Rome III	All	4	L. casei rhamnosus LCR35	6×10^8	26	26
Cui, 2012	China	Rome III	All	4	a multi-strain preparation (2)	NA	37	23
Cha, 2012	Korea	Rome III	D	8	a multi-strain preparation (7)	1×10^{10} cells/day	25	25
Sondergaard, 2011	Danish, Sweden	Rome II	All	8	a multi-strain preparation (5)	$7{\cdot}5 \times 10^{10}$	32	25
Michail, 2011	USA	Rome III	D	24	a multi-strain preparation (8)	9×10^{11} bacteria/day	15	9
Guglielmetti, 2011	Germany	Rome III	All	4	B. bifidum MIMBb75	1×10^9	60	62
Choi, 2011	Korea	Rome II	D, M	4	S. boulardii	8×10^{11} bacteria/day	45	45
Simren, 2010	Sweden	Rome II	All	8	a multi-strain preparation (3)	2×10^{10}	37	37
Ligaarden, 2010	Norway	Rome II	All	3	L. plantarum MF1298	1×10^{10}	10	9
Hun, 2009	USA	Rome II	D	8	B. coagulans GBI-30, 6086	8×10^8	22	22
Hong, 2009	Korea	Rome III	All	8	a multi-strain preparation (4)	4×10^{10}	36	34
Enck, 2009	Germany	primary care criteria	All	8	E. coli (DSM 17252)	$6{\cdot}75\text{-} 13{\cdot}5 \times 10^7$	148	150
Dolin, 2009	USA	Rome III	D	8	B. coagulans GBI-30, 6086	$2{\cdot}0 \times 10^9$	26	29
Zeng, 2008	China	Rome II	D	4	a multi-strain preparation (4)	$5{\cdot}2 \times 10^{10}$	14	15
Williams, 2008	UK	Rome II	All	8	a multi-strain preparation (4)	$2{\cdot}5 \times 10^{10}$	28	28

Sinn, 2008	Korea	Rome III	All	4	L. acidophilus-SDC 2012, 2013	$2{\cdot}9 \times 10^9$	20	20
Kajander, 2008	Finland	Rome II	All	20	a multi-strain preparation (4)	$1{\cdot}2 \times 10^9$	43	43
Enck, 2008	Germany	primary care criteria	All	8	a multi-strain preparation (2)	$9\text{-}27 \times 10^7$	149	148
Drouault-Holowacz, 2008	France	Rome II	All	4	a multi-strain preparation (3)	1×10^{10}	53	53
Andriulli, 2008	Italy	Rome II	All	12	L. paracasei strain B21060	1×10^{10}	132	135
Agrawal, 2008	France	Rome III	C	4	a multi-strain preparation (3)	$2{\cdot}74 \times 10^{10}$	17	17
Guyonnet, 2007	France	Rome II	C	6	a multi-strain preparation (3)	$2{\cdot}74 \times 10^{10}$	137	137
Whorwell, 2006	UK	Rome II	All	4	B. infantis 35624	1×10^{10}	90	92
O'Mahony, 2005	Ireland	Rome II	All	8	a multi-strain preparation (2)	1×10^{10} bacteria/day	50	25
Niv, 2005	Israel	Rome II	All	22	L. reuteri ATCC 55730	2×10^8	27	27
Kim, 2005	USA	Rome II	All	4	a multi-strain preparation (8)	$4{\cdot}5 \times 10^{11}$ bacteria/day	24	24
Kajander, 2005	Finland	Rome I	All	24	a multi-strain preparation (4)	$8\text{–}9 \times 10^9$	52	51
Kim, 2003	USA	Rome II	D	8	a multi-strain preparation (8)	$4{\cdot}5 \times 10^{11}$	12	13
Niedzielin, 2001	Poland	Manning criteria	All	4	L. plantarum 299V	2×10^{10}	20	20
Nobaek, 2000	USA	Rome I	All	4	L. plantarum DSM 9843	5×10^7	30	30

IBS subtype: D, IBS with predominant diarrhea; C, IBS with predominant constipation. W, week; CFU, colony-forming unit; I, intervention group; C, control group.

Lactobacillus paracasei, L. paracasei; Saccharomyces cerevisiae, S. cerevisiae; Lactiplantibacillus plantarum (Lactobacillus plantarum), L. plantarum; Bacillus coagulans, B. coagulans; Lactobacillus acidophilus, L. acidophilus; Bifidobacterium animalis subsp. lactis, B. lactis; Bifidobacterium longum, B. longum; Bifidobacterium bifidum, B. bifidum; Clostridium butyricum, C. butyricum; Lactobacillus gasseri, L. gasseri; Bacillus clausii, B. clausii; Lactobacillus rhamnosus, L. rhamnosus; Bifidobacterium infantis, B. infantis; Lactobacillus casei, L. casei; Saccharomyces boulardii, S. boulardii; Escherichia coli, E. coli; Lactobacillus reuteri, L. reuteri.

Table S2. Characteristics of Included Randomized Controlled Trials (2)

Authors	Public-ation years	Sample size (I)	No. of female	Age	BMI	Sample size(C)	No. of female	Age	BMI
Bai	2023	145	70	42·41 (11·92)	23·10 (3·14)	145	60	45·74 (12·40)	23·69 (2·75)
Mourey	2022	230	202	41·2 ± 13·96	NA	226	190	39·9 ± 14·56	NA
Jung	2022	18	12	39·84 (6·44)	24·00 (3·44)	9	6	39·38 (3·16)	24·40 (3·84)
Xu	2021	24	14	35·58±13·28	NA	24	11	38·29±15·84	NA
Skrzydlo-Radomanska	2021	25	17	45·5 ± 11·1	26·5 ± 4·7	26	14	40·7 ± 14·4	25·2 ± 4·2
Liu	2021	25	9	48·57 ±11·14	25·14 ± 4·14	25	11	48·95 ± 9·38	25·42 ± 4·50
Gupta	2021	20	7	36·20±9·81	23·37±3·23	20	5	34·80±11·06	24·03±2·99
Barraza-Ortiz	2021	18	12	45·1±9·2	26·8±5·2	18	10	46·1±5·2	24·6±3·4
Sadrin	2020	40	29	48·9 ± 8·4	NA	40	28	48·9 ± 8·0	NA
Martoni	2020	111	58	39·41 (11·80)	24·09 (4·34)	109	54	37·61 (10·12)	24·10 (4·06)
		110	51	41·60 (11·11)	23·78 (3·98)			NA	NA
Lewis	2020	95	67	42·42 ± 12·30	NA	95	64	41·84 ± 16·14	NA
		95	64	42·31 ± 16·88	NA			NA	NA
Kim	2020	32	26	39 (23 – 73)	NA	31	21	33 (22 - 58)	NA
Gayathri	2020	52	20	42·25 ± 15·44	NA	48	14	39·60 ± 12·79	NA
Bonfrate	2020	15	12	50 ± 11	24·3 ± 6·2	15	6	46 ± 10	23·8 ± 5·4
Andresen	2020	221	155	40·1 (12·8)	24·5 (5·3)	222	152	42·6 (13·8)	24·7 (5·0)
Oh	2019	28	17	32·5 (26·5–39·0)	22·2 (19·9–23·6)	27	19	33·0(28·0–44·5)	20·7 (18·3–22·3)
Madempudi	2019	68	12	44·4(20-60)	NA	68	18	42·3 (20-60)	NA
Ashtiani	2019	30	16	31·2 ±12·3	NA	30	13	31·44 ± 7·6	NA
Sun	2018	105	42	43·00 (12·45)	NA	95	42	44·91 (13·01)	NA
Shin	2018	30	12	35·0 (32·0, 40·5)	23·9 (21·6-27·6)	30	17	38·0 (30·0- 46·0)	22·9 (20·1, 27·9)
Preston	2018	76	47	40·6±13·4	NA	37	21	39·9 14·0	NA
Kim	2018	10	3	28·6 ± 2·4	24·9 ± 0·5	12	6	26·7 ± 2·3	25·5 ± 0·6
Ishaque et al.	2018	181	45	32·2 ± 10·1	NA	179	34	31·7 ± 9·7	NA
Cremon et al	2018	20	11	37·35 ± 11·25	NA	20	15	44·55 ± 12·98	NA
Pinto-Sanchez	2017	18	12	46·5 (30–58)	NA	20	12	40·0 (26–57)	NA
Nobutani	2017	17	9	52·6 ± 20·1	NA	17	10	45·9±19·5	NA
Hod	2017	54	54	29·0(25·0-45·0)	22·1(19·9-25·4)	53	53	30·0, 24·0- 42·0	22·5(19·5-24·8)
Thijssen	2016	39	26	41·1 ± 14·8	NA	41	29	42·4 ± 13·5	NA
Spiller	2016	192	161	45·3 (15·7)	NA	187	156	45·4 (14·1)	NA
Mezzasalma	2016	53	NA	36·0±11·9	NA	52	NA	38·1±13·5	NA
		52	NA	38·0±12·1	NA			NA	NA
Majeed	2016	18	11	36·2 ± 11·07	24·6 ± 3·14	18	8	35·4 ± 10·75	24·1 ± 3·98
Lyra	2016	129	94	47·1 ± 13·3	24·7 ± 3·7	131	94	49·4 ± 12·9	24·9 ± 3·7
Yoon	2015	39	20	59·9 ± 11·1	NA	42	18	58·8 ± 13·3	NA
Wong	2015	20	8	40·86 (3·51)	NA	22	11	53·35 (4·15)	NA
de Chambrun	2015	93	72	42·5 ± 12·5	NA	86	82	45·4 ± 14	NA
Yoon	2014	25	14	45·9 ± 13·7	NA	24	18	43·1 ± 15·1	NA
Stevenson	2014	54	52	48·15 ± 13·48	28·83 ± 7·12	27	27	47·27 ± 12·15	28·88 ± 7·74

Sisson	2014	124	84	39·1 (10·5)	NA	62	45	36·8 (10·8)	NA
Shavakhi	2014	66	46	36·1±7 ·9	NA	63	39	36·4±10·5	NA
Ludidi	2014	21	15	40·0± 2·2	25·8 ±1·1	19	12	41·1± 4·2	25·1± 0·9
Lorenzo-Zuniga	2014	55	19	47·5 ± 13·1	24·7 ± 3·9	29	14	46·5 ± 13·1	26·4 ± 5·2
Jafari	2014	54	33	36·6 ± 12·1	NA	54	32	36·8 ± 11·0	NA
Abbas	2014	37	10	37·7±11·6	NA	35	9	33·0±12·0	NA
Roberts	2013	88	73	44·66 (11·98)	26·75 (4·03)	91	76	43·71 (12·76)	25·85 (3·61)
Lee	2013	46	32	33·1±7·6	NA	39	21	32·7±7·8	NA
Charbonneau	2013	39	31	47·0 ± 1·96	NA	37	31	43·2 ± 2·01	NA
Cappello	2013	34	22	36·6±2·2	NA	34	19	40·8±2·2	NA
Begtrup	2013	67	51	31·63 (10·05)	24·68 (4·60)	64	46	29·38 (8·64)	24·42(3·64)
Amirimani	2013	41	20	44·9±13·0	NA	31	16	37·7 ± 10·5	NA
Kruis	2012	60	48	46·3±12·1	NA	60	44	45·1±12·7	NA
Ducrotte	2012	108	38	36·53 ± 12·08	NA	106	25	38·40 ± 13·13	NA
Dapoigny	2012	26	15	46·1 ± 11·3	23·4 ± 4·9	26	20	48·0 ± 10·8	24·5 ± 4·0
Cui	2012	37	26	42·92±15·09	22·08±3·23	23	16	47 ·45±15·36	20·77±2·65
Cha	2012	25	13	37·9 ± 12·4	23·03 ± 3·31	25	11	40·3 ± 11·2	22·93 ± 2·88
Sondergaard	2011	32	20	53·9 (29–67)	24·7, 3·4	25	19	48·5 (29–67)	24·8, 4·3
Michail	2011	15	10	21·8±17	NA	9	6	21·8±17	NA
Guglielmetti	2011	60	41	36·65± 12·42	24·60 ± 5·19	62	41	40·98 ± 12·80	24·02 ± 4·45
Choi	2011	45	17	40·2±13·1	NA	45	20	40·6±12·9	NA
Simren	2010	37	26	42±15	NA	37	26	44± 16	NA
Ligaarden	2010	10	NA	50 (11)	2 4 (3)	9	NA	50 (11)	2 4 (3)
Hun	2009	22	18	48·3（23–70）	NA	22	18	48·36（23–70）	NA
Hong	2009	36	11	36±2	NA	34	12	38±3	NA
Enck	2009	148	72	49·8 (19 – 70)	24·4 (17·2 – 37·6)	150	75	49·4 (18 – 76)	24·0(17·1–33·2)
Dolin	2009	26	19	52·3 ± 11·1	NA	29	23	44·0 ± 17·9	NA
Zeng	2008	14	4	44·6 ± 12·4	NA	15	6	45·8 ± 9·2	NA
Williams	2008	28	25	40 (12)	NA	28	20	38 (11)	NA
Sinn	2008	20	14	41·9 ± 14·4	21·9 ± 3·6	20	12	47·5 ± 11·0	22·4 ± 2·2
Kajander	2008	43	41	50 (13)	25·5 (3·4)	43	39	46 (13)	26·8 (5·4)
Enck	2008	149	72	49·8 (19–70)	24·4 (17·2–37·6)	148	75	49·4 (18–76)	24·0 (17·1–33·2)
Drouault-Holowacz	2008	53	40	47 ± 14	NA	53	36	44 ± 14	NA
Andriulli	2008	132	96	42·5 (12·93)	NA	135	90	42·8 (12·10)	NA
Agrawal	2008	17	17	42 (24, 69)	25·0 (20·3, 29·6)	17	17	37 (20, 59)	25·0 (20·3–29·6)
Guyonnet	2007	137	106	49·4（11·4）	NA	137	93	49·2（11·4）	NA
Whorwell	2006	90	90	41·8 (1·10)	NA	92	92	42·4 (1·09)	NA
O'Mahony	2005	50	32	44·3	NA	25	16	44·3(18-73)	NA
Niv	2005	27	20	45·7±14·2	NA	27	16	45·6+16·1	NA
Kim	2005	24	21	40 ± 3	NA	24	24	46 + 3	NA
Kajander	2005	52	39	46 (23–65)	25·7(16·9–36·6)	51	40	45 (21–65)	24·4(16·3–39·3)
Kim	2003	12	10	48 ± 5·7	NA	13	8	38 ± 3·4	NA
Niedzielin	2001	20	15	45 ± 18	23·3±4·5	20	17	42 ±15	24·1 ± 2·7

Nobaek	2000	30	16	52(24-78)	NA	30	20	46(21-66)	NA

I, intervention group; C, control group; BMI, body mass index.

Values are reported as mean ± standard deviation, mean ±standard error, median (range), or median (interquartile range)

Table S3 Critical results of pairwise meta-analysis

MD, mean difference; SMD, standardized mean difference; CI, confidence intervals; L95%Cl, 95% confidence lower limit; U95% Cl, 95% confidence upper limit;

1. IBS-SSS

Treatment	number of studies	MD	[95% Conf. Interval]		% Weight	Heterogeneity statistic	degrees of freedom	P	I-squared**	Tau-squared	z	p(Z)
L. paracasei HA-196	1	-0.3	-20.822	20.222	5.72	0	0	.	.%	0	0.03	0.977
L. plantarum CCFM8610	1	0	-31.719	31.719	4.96	0	0	.	.%	0	0	1
L. plantarum 299v	1	-5.94	-58.975	47.095	3.52	0	0	.	.%	0	0.22	0.826
L. acidophilus DDS-1	1	-77.7	-101.717	-53.683	5.5	0	0	.	.%	0	6.34	0
L. acidophilus ATCC 700396	1	-6.8	-28.503	14.903	5.65	0	0	.	.%	0	0.61	0.539
B. lactis UABla-12	1	-48.8	-72.999	-24.601	5.49	0	0	.	.%	0	3.95	0
B. longum R0175	1	21.16	1.673	40.647	5.79	0	0	.	.%	0	2.13	0.033
B. bifidum MIMBb75	1	-29.83	-48.237	-11.423	5.85	0	0	.	.%	0	3.18	0.001
C. butyricum CGMCC0313.1	1	-21.38	-40.466	-2.294	5.81	0	0	.	.%	0	2.2	0.028
L. casei LCR35	1	1.1	-52.324	54.524	3.49	0	0	.	.%	0	0.04	0.968
BL+LR	1	-81	-130.739	-31.261	3.72	0	0	.	.%	0	3.19	0.001
LC+LP+BAL	1	-76.427	-114.905	-37.949	4.48	0	0	.	.%	0	3.89	0
BAL+ST+LB	2	23.037	-13.008	59.082	7.75	1.21	1	0.271	17.50%	132.0778	1.25	0.21
EF+LA+X	1	-35	-60.44	-9.56	5.4	0	0	.	.%	0	2.7	0.007
BAL+LA+BB+X	2	-26.745	-55.87	2.379	10.18	1.93	1	0.165	48.10%	281.9875	1.8	0.072
LR+pf+X	1	23.93	12.849	35.011	6.21	0	0	.	.%	0	4.23	0
LP+LC+BL+ST+LA+X	2	-63.963	-78.664	-49.262	10.46	0.04	1	0.836	0.00%	0	8.53	0

2. IBS-QOL

Study	number of studies	MD	[95% Conf. Interval]		% Weight	Heterogeneity statistic	degrees of freedom	P	I-squared**	Tau-squared	Z	p(Z)
L. paracasei HA-196	1	0.4	-2.795	3.595	12.01	0	0	.	.%	0	0.25	0.806
C. butyricum CGMCC0313.1	1	4.073	0.496	7.65	11.89	0	0	.	.%	0	2.23	0.026
S. boulardii CNCM I-475	1	5.7	-1.067	12.467	10.63	0	0	.	.%	0	1.65	0.099
LC+LP+BAL	1	3.76	-4.814	12.334	9.75	0	0	.	.%	0	0.86	0.39
LPA+LA+BAL	1	0.5	-5.973	6.973	10.76	0	0	.	.%	0	0.15	0.88
BAL+ST+LB	1	1.6	-2.594	5.794	11.69	0	0	.	.%	0	0.75	0.455
EF+LA+X	1	1.6	-2.513	5.713	11.72	0	0	.	.%	0	0.76	0.446
LA+LR+ST+BL+bbr+X	1	3.5	-4.895	11.895	9.84	0	0	.	.%	0	0.82	0.414
LP+LC+BL+ST+LA+X	1	24.8	20.647	28.953	11.71	0	0	.	.%	0	11.7	0

3. HADS-total score (a), HADS-anxiety (b), and HADS-depression (c)

Treatment	number of studies	MD	[95% Conf.	Interval]	% Weight	Heterogeneity statistic	degrees of freedom	P	I-squared**	Tau-squared	Z	p(Z)
**HADS total score**												
L. paracasei HA-196	1	0.031	-0.122	0.184	45.83	0	0	.	.%	0	0.4	0.691
L. acidophilus ATCC 700396	1	-0.6	-1.75	0.55	7.49	0	0	.	.%	0	1.02	0.306
B. longum R0175	1	-0.342	-0.479	-0.205	46.67	0	0	.	.%	0	4.89	0
**HADS anxiety**												
L. acidophilus ATCC 700396	1	-0.2	-0.889	0.489	47.81	0	0	.	.%	0	0.57	0.569
B. longum NCC3001	1	-1.5	-3.56	0.56	23.04	0	0	.	.%	0	1.43	0.153
LPA+LA+BAL	1	1.4	-0.249	3.049	29.15	0	0	.	.%	0	1.66	0.096
**HADS depression**												
L. paracasei CNCM I-1572	1	-0.63	-1.839	0.579	25.39	0	0	.	.%	0	1.02	0.307
L. acidophilus ATCC 700396	1	-0.3	-0.884	0.284	37.48	0	0	.	.%	0	1.01	0.314
B. longum NCC3001	1	-3	-4.918	-1.082	15.5	0	0	.	.%	0	3.07	0.002

4. Abdominal pain score

treatment	number of studies	SMD	[95% Conf.		Interval]	% Weight	Heterogeneity statistic	degrees of freedom	P	I-squared**	Tau-squared	Z
L. plantarum CCFM8610	1	10	-0.125	20.125	2.01	0	0	.	.%	0	1.94	0.053
S. cerevisiae CNCM I-3856	3	-15.816	-21.673	-9.959	5.01	1.7	1	19.20%	41.20%	8.1822	5.29	0
L. plantarum Apsulloc 331261	1	-26.6	-43.497	-9.703	1.25	0	0	.	.%	0	3.09	0.002
L. plantarum 299v	1	-4	-11.147	3.147	2.42	0	0	.	.%	0	1.1	0.273
B. coagulans Unique IS2	1	-32	-38.648	-25.352	2.49	0	0	.	.%	0	9.43	0
B. coagulans MTCC 5856	1	-41.8	-57.855	-25.745	1.32	0	0	.	.%	0	5.1	0
L. acidophilus DDS-1	1	-19.53	-27.337	-11.723	2.33	0	0	.	.%	0	4.9	0
L. acidophilus ATCC 700396	1	-3	-9.022	3.022	2.57	0	0	.	.%	0	0.98	0.329
L. acidophilus SDC 2012,2013	1	-12	-19.252	-4.748	2.41	0	0	.	.%	0	3.24	0.001
B. lactis UABla-12	1	-11.11	-19.466	-2.754	2.25	0	0	.	.%	0	2.61	0.009
B. bifidum MIMBb75	2	-7.014	-13.939	-0.09	5.68	6.82	1	0.90%	85.30%	21.326	1.99	0.047
C. butyricum CGMCC0313.1	1	-6.05	-16.286	4.186	1.99	0	0	.	.%	0	1.16	0.247
L. gasseri BNR17	1	-36.1	-62.071	-10.129	0.69	0	0	.	.%	0	2.72	0.006
B. infantis 35624	1	7.4	-1.192	15.992	2.22	0	0	.	.%	0	1.69	0.091
L. casei LCR35	1	-1.2	-14.645	12.245	1.59	0	0	.	.%	0	0.17	0.861
S. boulardii CNCM I-475	2	3.563	-4.871	11.998	4.12	1.51	1	0.219	33.80%	12.9656	0.83	0.408
LA+LA	1	-6.2	-13.269	0.869	2.43	0	0	.	.%	0	1.72	0.086
BL+LR	1	-17	-29.97	-4.03	1.65	0	0	.	.%	0	2.57	0.01
LPA+ls+LP	1	-20	-31.005	-8.995	1.89	0	0	.	.%	0	3.56	0
LPA+LA+BAL	2	1.313	-5.812	8.438	3.76	0.98	1	0.322	0.00%	0	0.36	0.718
BAL+ST+LB	4	-0.588	-5.173	3.997	8.74	4.1	3	25.10%	26.80%	6.0342	0.25	0.801
LP+LP+pa	1	-7	-10.331	-3.669	2.88	0	0	.	.%	0	4.12	0
EF+LA+X	2	-10.953	-19.947	-1.96	4.25	1.75	1	0.186	42.70%	24.9241	2.39	0.017
LA+ST+X	3	-7.634	-10.875	-4.394	7.92	2.24	2	32.60%	10.90%	0.9214	4.62	0
BAL+LA+BB+X	1	-11.13	-23.238	0.978	1.75	0	0	.	.%	0	1.8	0.072
LR+pf+X	1	-10.71	-19.369	-2.051	2.21	0	0	.	.%	0	2.42	0.015
BAL+LA+LR+X	3	2.136	-13.231	17.503	5.09	8.94	2	0.011	77.60%	141.8685	0.27	0.785
LA+LR+ST+BL+bbr+X	3	-7.448	-15.712	0.815	5.45	3.08	2	21.40%	35.20%	18.8069	1.77	0.077
VSL#3	2	-12.794	-22.901	-2.688	3.14	1.1	1	29.50%	8.70%	4.65	2.48	0.013
LP+LC+BL+ST+LA+X	4	-6.742	-18.468	4.984	8.48	23.54	3	0.00%	87.30%	118.7792	1.13	0.26

5. Abdominal bloating score

treatment	number of studies	SMD	[95% Conf.	Interval]	% Weight	Heterogeneity statistic	degrees of freedom	P	I-squared**	Tau-squared	p	p(Z)
L. plantarum CCFM8610	1	-20	-31.098	-8.902	2.58	0	0	.	.%	0	3.53	0
S. cerevisiae CNCM I-3856	1	-8.34	-25.955	9.275	1.51	0	0	.	.%	0	0.93	0.353
L. plantarum Apsulloc 331261	1	-22.7	-46.998	1.598	0.94	0	0	.	.%	0	1.83	0.067
L. plantarum 299v	1	-14.79	-24.912	-4.668	2.79	0	0	.	.%	0	2.86	0.004
B. coagulans Unique IS2	1	-8	-18.397	2.397	2.73	0	0	.	.%	0	1.51	0.132
B. coagulans MTCC 5856	1	-30.67	-66.889	5.549	0.47	0	0	.	.%	0	1.66	0.097
L. acidophilus DDS-1	1	-11.73	-20.84	-2.62	3.03	0	0	.	.%	0	2.52	0.012
L. acidophilus ATCC 700396	1	-1.1	-8.081	5.881	3.57	0	0	.	.%	0	0.31	0.757
B. lactis UABla-12	1	-6.43	-15.718	2.858	2.99	0	0	.	.%	0	1.36	0.175
B. bifidum MIMBb75	1	-11.83	-16.371	-7.289	4.18	0	0	.	.%	0	5.11	0
C. butyricum CGMCC0313.1	1	-0.956	-5.864	3.952	4.1	0	0	.	.%	0	0.38	0.703
B. infantis 35624	1	1.4	-7.428	10.228	3.1	0	0	.	.%	0	0.31	0.756
S. boulardii CNCM I-475	2	-1.181	-9.73	7.368	4.73	0.31	1	0.579	0.00%	0	0.27	0.787
BL+LR	2	-34	-54.59	-13.41	1.21	0	0	.	.%	0	3.24	0.001
LPA+LA+BAL	2	5.909	-1.69	13.507	5.04	0.1	1	0.757	0.00%	0	1.52	0.128
BAL+ST+LB	4	-0.618	-4.645	3.409	11.14	1.94	3	0.586	0.00%	0	0.3	0.764
EF+LA+X	1	-4.6	-12.051	2.851	3.45	0	0	.	.%	0	1.21	0.226
LA+ST+X	1	-9.3	-14.955	-3.645	3.91	0	0	.	.%	0	3.22	0.001
BAL+LA+BB+X	1	-8	-22.207	6.207	1.99	0	0	.	.%	0	1.1	0.27
LR+pf+X	1	-10.71	-20.504	-0.916	2.87	0	0	.	.%	0	2.14	0.032
BAL+LA+LR+X	5	-1.911	-9.478	5.657	6.29	1.83	2	0.401	0.00%	0	0.49	0.621
LA+LR+ST+BL+bbr+X	3	-2.721	-9.348	3.905	7.32	1.97	2	0.374	0.00%	0	0.8	0.421
VSL#3	3	-13.617	-19.48	-7.754	8.1	0.43	2	0.805	0.00%	0	4.55	0
LP+LC+BL+ST+LA+X	4	-4.49	-16.933	7.953	11.94	25.61	3	0	88.30%	134.8589	0.71	0.479

6. Bowel movement frequency (per week) in IBS-D and IBS-D

treatment	number of studies	MD	[95% Conf.	Interval]	% Weight	Heterogeneity statistic	degrees of freedom	P	I-squared**	Tau-squared	Z	p(Z)
**Bowel movement frequency (per week) in IBS-D**												
L. paracasei HA-196	1	-3.127	-4.628	-1.626	11.56	0	0	.	.%	0	4.08	0
L. paracasei B21060	1	-5.11	-9.983	-0.237	3.78	0	0	.	.%	0	2.06	0.04
S. cerevisiae CNCM I-3856	1	0	-2.707	2.707	7.78	0	0	.	.%	0	0	1
L. plantarum Apsulloc 331261	1	-2.05	-4.786	0.686	7.7	0	0	.	.%	0	1.47	0.142
B. coagulans GBI-30,6086	1	-2.114	-2.996	-1.232	13.46	0	0	.	.%	0	4.7	0
B. longum R0175	1	-1.953	-3.453	-0.453	11.56	0	0	.	.%	0	2.55	0.011
C. butyricum CGMCC0313.1	1	-0.516	-1.06	0.028	14.22	0	0	.	.%	0	1.86	0.063
S. boulardii CNCM I-475	2	2.246	-0.464	4.956	10.23	0.02	1	0.877	0.00%	0	1.62	0.104
EF+LA+X	1	-3.948	-5.019	-2.877	12.93	0	0	.	.%	0	7.23	0
LP+LC+BL+ST+LA+X	1	-0.7	-3.797	2.397	6.79	0	0	.	.%	0	0.44	0.658
**Bowel movement frequency (per week) in IBS-C**												
treatment	number of studies	MD	[95% Conf.	Interval]	% Weight	Heterogeneity statistic	degrees of freedom	P	I-squared**	Tau-squared	Z	p(Z)
L. paracasei HA-196	1	1.341	0.499	2.183	15.05	0	0	.	.%	0	3.12	0.002
S. cerevisiae CNCM I-3856	1	1.4	0.413	2.387	14.3	0	0	.	.%	0	2.78	0.005
B. coagulans Unique IS2	1	1.3	0.728	1.872	16.29	0	0	.	.%	0	4.45	0
B. longum R0175	1	0.838	-0.599	2.275	11.85	0	0	.	.%	0	1.14	0.253
B. bifidum MIMBb75	1	2.67	0.017	5.323	6.66	0	0	.	.%	0	1.97	0.049
BAL+ST+LB	2	0.019	-3.791	3.83	31.19	27.63	2	0	92.80%	10.3662	0.01	0.992
LA+ST+X	1	1.624	-1.829	5.077	4.66	0	0	.	.%	0	0.92	0.357

7. Bristol stool form scale in IBS-D and IBS-C

treatment	number of studies	MD	[95% Conf.	Interval]	% Weight	Heterogeneity statistic	degrees of freedom	P	I-squared**	Tau-squared	Z	p(Z)
**Bristol stool form scale in IBS-D**												
L. paracasei HA-196	1	-0.25	-1.074	0.574	6.05	0	0	.	.%	0	0.59	0.552
S. cerevisiae CNCM I-3856	1	-1.245	-1.634	-0.856	9.28	0	0	.	.%	0	6.27	0
B. coagulans MTCC 5856	1	-3.276	-5.215	-1.337	2	0	0	.	.%	0	3.31	0.001
B. longum R0175	1	-0.06	-0.768	0.648	6.85	0	0	.	.%	0	0.17	0.868
B. bifidum MIMBb75	1	-0.2	-0.411	0.011	10.4	0	0	.	.%	0	1.86	0.063
C. butyricum CGMCC0313.1	1	-0.112	-0.478	0.254	9.44	0	0	.	.%	0	0.6	0.548
S. boulardii CNCM I-475	2	0.499	0.082	0.915	15.45	0.18	1	0.669	0.00%	0	2.35	0.019
BL+LR	1	-0.8	-1.566	-0.034	6.44	0	0	.	.%	0	2.05	0.041
LP+LP+pa	1	-0.5	-0.761	-0.239	10.13	0	0	.	.%	0	3.75	0
LA+LR+ST+BL+bbr+X	1	-0.7	-1.324	-0.076	7.48	0	0	.	.%	0	2.2	0.028
VSL#3	1	-0.2	-0.817	0.417	7.53	0	0	.	.%	0	0.63	0.526
LP+LC+BL+ST+LA+X	1	0.36	-0.071	0.791	8.96	0	0	.	.%	0	1.64	0.102
**Bristol stool form scale in IBS-C**												
treatment	number of studies	MD	[95% Conf.	Interval]	% Weight	Heterogeneity statistic	degrees of freedom	P	I-squared**	Tau-squared	Z	Z(p)
L. paracasei HA-196	1	1.03	0.089	1.971	15.83	0	0	.	.%	0	2.15	0.032
S. cerevisiae CNCM I-3856	1	1.17	0.235	2.105	15.94	0	0	.	.%	0	2.45	0.014
B. longum R0175	1	0.13	-1.344	1.604	8.51	0	0	.	.%	0	0.17	0.863
BAL+ST+LB	2	0.34	-0.393	1.072	59.72	5.8	1	0.016	82.80%	0.2315	0.91	0.364

Table S4 League Table Demonstrating the Relative Efficacy for Each Pair of Comparison in the NMA.

League table demonstrating the relative efficacy for each pair of comparisons for abdominal pain score (blue background) and abdominal bloating score (green background). Comparisons should start with longitudinal probiotics and compare with horizontal probiotics. Bold values denote a statistically significant difference.

1. IBS-SSS

L. paracasei HA-196																	
-0.31 (-38.08,37.46)	L. plantarum CCFM8610																
5.63 (-51.23,62.49)	5.94 (-55.86,67.74)	L. plantarum 299v															
77.39 (45.81,108.98)	77.70 (37.91,117.49)	71.76 (13.54,129.98)	L. acidophilus DDS-1														
6.49 (-23.37,36.35)	6.80 (-31.63,45.23)	0.86 (-56.44,58.16)	-70.90 (-103.27,-38.53)	L. acidophilus ATCC 700396													
48.49 (16.77,80.21)	48.80 (8.90,88.70)	42.86 (-15.43,101.15)	-28.90 (-54.12,-3.68)	42.00 (9.49,74.50)	B. lactis UABla-12												
-21.46 (-42.11,-0.82)	-21.16 (-58.38,16.07)	-27.10 (-83.60,29.40)	-98.86 (-129.78,-67.93)	-27.96 (-57.12,1.21)	-69.96 (-101.02,-38.89)	B. longum R0175											
29.52 (1.96,57.08)	29.83 (-6.84,66.50)	23.89 (-32.25,80.03)	-47.87 (-78.13,-17.61)	23.03 (-5.43,51.49)	-18.97 (-49.37,11.43)	50.99 (24.18,77.79)	B. bifidum MIMBb75										
21.07 (-6.95,49.09)	21.38 (-15.64,58.40)	15.44 (-40.92,71.80)	-56.32 (-87.00,-25.64)	14.58 (-14.32,43.48)	-27.42 (-58.24,3.40)	42.54 (15.26,69.81)	-8.45 (-34.97,18.07)	C. butyricum CGMCC0313.1									
-1.41 (-58.63,55.82)	-1.10 (-63.23,61.03)	-7.04 (-82.32,68.24)	-78.80 (-137.37,-20.23)	-7.90 (-65.56,49.76)	-49.90 (-108.55,8.75)	20.06 (-36.81,76.92)	-30.93 (-87.44,25.58)	-22.48 (-79.21,34.25)	L. casei LCR35								
80.69 (26.89,134.49)	80.99 (22.00,139.98)	75.05 (2.35,147.76)	3.29 (-51.94,58.53)	74.19 (19.93,128.46)	32.19 (-23.12,87.51)	102.15 (48.73,155.57)	51.16 (-1.87,104.20)	59.61 (6.34,112.89)	82.09 (9.10,155.09)	BL+LR							
76.12 (32.51,119.72)	76.42 (26.56,126.29)	70.48 (4.96,136.01)	-1.27 (-46.63,44.08)	69.62 (25.45,113.80)	27.63 (-17.83,73.08)	97.58 (54.45,140.71)	46.59 (3.94,89.25)	55.04 (12.09,98.00)	77.52 (11.69,143.36)	-4.57 (-67.45,58.31)	LC+LP+BAL						
-24.67 (-62.54,13.21)	-24.36 (-69.30,20.58)	-30.30 (-92.16,31.56)	-102.06 (-141.94,-62.18)	-31.16 (-69.69,7.37)	-73.16 (-113.15,-33.17)	-3.20 (-40.53,34.13)	-54.19 (-90.97,-17.41)	-45.74 (-82.86,-8.62)	-23.26 (-85.45,38.93)	-105.36 (-164.41,-46.30)	-100.79 (-150.73,-50.84)	BAL+ST+LB					
34.69 (2.01,67.37)	35.00 (-5.66,75.66)	29.06 (-29.76,87.88)	-42.70 (-77.69,-7.71)	28.20 (-5.24,61.64)	-13.80 (-48.91,21.31)	56.16 (24.11,88.20)	5.17 (-26.23,36.57)	13.62 (-18.18,45.42)	36.10 (-23.07,95.27)	-45.99 (-101.86,9.87)	-41.43 (-87.55,4.70)	59.36 (18.60,100.12)	EF+LA+X				
18.56 (-3.12,40.23)	18.86 (-13.62,51.35)	12.92 (-40.57,66.42)	-58.84 (-83.86,-33.82)	12.06 (-10.74,34.87)	-29.94 (-55.13,-4.74)	40.02 (19.31,60.73)	-10.97 (-30.66,8.73)	-2.52 (-22.85,17.82)	19.96 (-33.92,73.84)	-62.13 (-112.36,-11.90)	-57.56 (-96.67,-18.45)	43.22 (10.62,75.83)	-16.14 (-42.52,10.25)	BAL+LA+BB+X			
-24.24 (-47.55,-0.92)	-23.93 (-57.53,9.67)	-29.87 (-84.05,24.31)	-101.63 (-128.08,-75.18)	-30.73 (-55.10,-6.36)	-72.73 (-99.34,-46.11)	-2.77 (-25.19,19.64)	-53.76 (-75.24,-32.27)	-45.31 (-67.38,-23.24)	-22.83 (-77.39,31.73)	-104.92 (-155.88,-53.97)	-100.35 (-140.40,-60.31)	0.43 (-33.28,34.14)	-58.93 (-86.68,-31.18)	-42.79 (-55.91,-29.68)	LR+PF+X		
63.65 (38.42,88.89)	63.96 (29.00,98.92)	58.02 (2.99,113.06)	-13.74 (-41.90,14.42)	57.16 (30.95,83.38)	15.16 (-13.15,43.48)	85.12 (60.71,109.53)	34.13 (10.58,57.69)	42.58 (18.49,66.67)	65.06 (9.65,120.47)	-17.03 (-68.90,34.83)	-12.46 (-53.65,28.73)	88.32 (53.25,123.39)	28.96 (-0.42,58.34)	45.10 (28.81,61.39)	87.89 (69.48,106.30)	LP+LC+BL+ST+LA+X	
-0.31 (-20.82,20.20)	-0.00 (-31.72,31.72)	-5.94 (-58.97,47.09)	-77.70 (-101.72,-53.68)	-6.80 (-28.50,14.90)	-48.80 (-73.00,-24.60)	21.16 (1.67,40.64)	-29.83 (-48.24,-11.42)	-21.38 (-40.47,-2.29)	1.10 (-52.32,54.52)	-80.99 (-130.73,-31.26)	-76.42 (-114.90,-37.95)	24.36 (-7.48,56.20)	-35.00 (-60.44,-9.56)	-18.86 (-25.88,-11.85)	23.93 (12.85,35.01)	-63.96 (-78.66,-49.26)	Placebo

2. IBS-QOL

L. acidophilus ATCC 700396									
-3.67 (-8.47,1.12)	C. butyricum CGMCC0313.1								
-5.30 (-12.78,2.18)	-1.63 (-9.28,6.03)	S. boulardii CNCM I-475							
-3.36 (-12.51,5.79)	0.31 (-8.98,9.60)	1.94 (-8.98,12.86)	LC+LP+BAL						
-0.10 (-7.32,7.12)	3.57 (-3.82,10.97)	5.20 (-4.16,14.56)	3.26 (-7.48,14.00)	LPA+LA+BAL					
-1.20 (-6.47,4.07)	2.47 (-3.04,7.99)	4.10 (-3.86,12.06)	2.16 (-7.39,11.70)	-1.10 (-8.81,6.61)	BAL+ST+LB				
-1.20 (-6.41,4.01)	2.47 (-2.98,7.92)	4.10 (-3.82,12.02)	2.16 (-7.35,11.67)	-1.10 (-8.77,6.57)	0.00 (-5.87,5.87)	EF+LA+X			
-3.10 (-12.08,5.88)	0.57 (-8.55,9.70)	2.20 (-8.58,12.98)	0.26 (-11.74,12.26)	-3.00 (-13.60,7.60)	-1.90 (-11.28,7.48)	-1.90 (-11.25,7.45)	LA+LR+ST+BL+BBR+X		
-24.40 (-29.64,-19.16)	-20.73 (-26.21,-15.25)	-19.10 (-27.04,-11.16)	-21.04 (-30.57,-11.51)	-24.30 (-31.99,-16.61)	-23.20 (-29.10,-17.30)	-23.20 (-29.04,-17.36)	-21.30 (-30.67,-11.93)	LP+LC+BL+ST+LA+X	
0.40 (-2.80,3.60)	4.07 (0.50,7.65)	5.70 (-1.07,12.47)	3.76 (-4.81,12.33)	0.50 (-5.97,6.97)	1.60 (-2.59,5.79)	1.60 (-2.51,5.71)	3.50 (-4.90,11.90)	24.80 (20.65,28.95)	Placebo

3. HADS-total score

L. paracasei HA-196			
0.63 (-0.53,1.79)	L. acidophilus ATCC 700396		
0.37 (0.23,0.52)	-0.26 (-1.42,0.90)	B. longum R0175	
0.03 (-0.13,0.19)	-0.60 (-1.75,0.55)	-0.34 (-0.48,-0.20)	Placebo

4. HADS-anxiety

L. acidophilus ATCC 700396			
1.30 (-0.87,3.47)	B. longum NCC3001		
-1.60 (-3.39,0.19)	-2.90 (-5.54,-0.26)	LPA+LA+BAL	
-0.20 (-0.89,0.49)	-1.50 (-3.56,0.56)	1.40 (-0.25,3.05)	Placebo

5. HADS-depression

L. paracasei CNCM I-1572				
-0.33 (-1.67,1.01)	L. acidophilus ATCC 700396			
2.37 (0.10,4.64)	2.70 (0.70,4.70)	B. longum NCC3001		
-0.63 (-2.51,1.25)	-0.30 (-1.85,1.25)	-3.00 (-5.40,-0.60)	LPA+LA+BAL	
-0.63 (-1.84,0.58)	-0.30 (-0.88,0.28)	-3.00 (-4.92,-1.08)	-0.00 (-1.44,1.44)	Placebo

6. Abdominal pain score

L. plantarum CCFM8610																														
25.07 (7.14,43.00)	S. cerevisiae CNCM I-3856																													
36.42 (10.87,61.97)	11.35 (-11.17,33.87)	L. plantarum Apsulloc 331261																												
13.83 (-6.63,34.29)	-11.24 (-27.76,5.27)	-22.59 (-47.18,1.99)	L. plantarum 299v																											
41.83 (21.53,62.12)	16.76 (0.45,33.06)	5.41 (-19.04,29.85)	28.00 (8.94,47.06)	B. coagulans Unique IS2																										
51.62 (26.62,76.63)	26.55 (4.65,48.45)	15.20 (-13.28,43.68)	37.80 (13.78,61.81)	9.80 (-14.07,33.67)	B. coagulans MTCC 5856																									
29.36 (8.66,50.06)	4.29 (-12.53,21.10)	-7.06 (-31.85,17.72)	15.53 (-3.96,35.02)	-12.47 (-31.78,6.85)	-22.27 (-46.49,1.95)	L. acidophilus DDS-1																								
12.83 (-7.27,32.92)	-12.24 (-28.31,3.82)	-23.59 (-47.87,0.69)	-1.00 (-19.85,17.85)	-29.00 (-47.66,-10.33)	-38.80 (-62.50,-15.09)	-16.53 (-35.64,2.58)	L. acidophilus ATCC 700396																							
21.83 (1.33,42.32)	-3.25 (-19.81,13.32)	-14.59 (-39.21,10.02)	8.00 (-11.28,27.27)	-20.00 (-39.10,-0.90)	-29.80 (-53.84,-5.75)	-7.53 (-27.06,12.00)	9.00 (-9.89,27.89)	L. acidophilus SDC 2012,2013																						
20.94 (0.02,41.85)	-4.13 (-21.21,12.94)	-15.48 (-40.45,9.48)	7.11 (-12.61,26.83)	-20.89 (-40.43,-1.35)	-30.69 (-55.09,-6.28)	-8.42 (-22.84,6.00)	8.11 (-11.23,27.45)	-0.89 (-20.64,18.87)	B. lactis UABla-12																					
16.88 (-0.65,34.42)	-8.19 (-20.91,4.54)	-19.54 (-41.75,2.68)	3.06 (-13.04,19.15)	-24.94 (-40.82,-9.06)	-34.74 (-56.32,-13.16)	-12.47 (-28.87,3.92)	4.06 (-11.57,19.68)	-4.94 (-21.08,11.20)	-4.05 (-20.72,12.61)	B. bifidum MIMBb75																				
15.88 (-5.86,37.61)	-9.19 (-27.27,8.88)	-20.54 (-46.20,5.11)	2.05 (-18.53,22.64)	-25.95 (-46.37,-5.53)	-35.75 (-60.85,-10.64)	-13.48 (-34.30,7.34)	3.05 (-17.17,23.27)	-5.95 (-26.57,14.67)	-5.06 (-26.10,15.98)	-1.01 (-18.69,16.68)	C. butyricum CGMCC0313.1																			
45.93 (13.65,78.21)	20.86 (-9.08,50.79)	9.51 (-25.53,44.55)	32.10 (0.58,63.62)	4.10 (-27.31,35.51)	-5.70 (-40.34,28.95)	16.57 (-15.11,48.24)	33.10 (1.82,64.38)	24.10 (-7.44,55.64)	24.99 (-6.83,56.80)	29.04 (-0.66,58.75)	30.05 (-2.31,62.41)	L. gasseri BNR17																		
2.43 (-18.58,23.44)	-22.64 (-39.84,-5.45)	-33.99 (-59.03,-8.95)	-11.40 (-31.22,8.42)	-39.40 (-59.04,-19.75)	-49.20 (-73.68,-24.71)	-26.93 (-47.00,-6.86)	-10.40 (-29.84,9.04)	-19.40 (-39.25,0.46)	-18.51 (-38.80,1.78)	-14.46 (-31.24,2.33)	-13.45 (-34.58,7.68)	-43.50 (-75.38,-11.62)	B. infantis 35624																	
11.03 (-12.39,34.44)	-14.04 (-34.11,6.02)	-25.39 (-52.49,1.70)	-2.80 (-25.15,19.56)	-30.80 (-53.00,-8.60)	-40.60 (-67.17,-14.02)	-18.33 (-40.90,4.24)	-1.80 (-23.82,20.22)	-10.80 (-33.19,11.59)	-9.91 (-32.68,12.86)	-5.86 (-25.57,13.86)	-4.85 (-28.38,18.67)	-34.90 (-68.41,-1.39)	8.60 (-14.26,31.46)	L. casei LCR35																
5.97 (-12.68,24.62)	-19.10 (-33.31,-4.90)	-30.45 (-53.55,-7.35)	-7.86 (-25.16,9.44)	-35.86 (-52.96,-18.76)	-45.66 (-68.15,-23.16)	-23.39 (-40.97,-5.81)	-6.86 (-23.73,10.01)	-15.86 (-33.20,1.48)	-14.97 (-32.80,2.86)	-10.92 (-24.64,2.81)	-9.91 (-28.70,8.88)	-39.96 (-70.33,-9.58)	3.54 (-14.40,21.48)	-5.06 (-25.77,15.65)	S. boulardii CNCM I-475															
16.03 (-4.41,36.46)	-9.04 (-25.53,7.44)	-20.39 (-44.95,4.17)	2.20 (-17.01,21.41)	-25.80 (-44.83,-6.77)	-35.60 (-59.59,-11.60)	-13.33 (-32.79,6.13)	3.20 (-15.62,22.02)	-5.80 (-25.05,13.45)	-4.91 (-24.60,14.78)	-0.86 (-16.92,15.20)	0.15 (-20.41,20.71)	-29.90 (-61.40,1.60)	13.60 (-6.19,33.39)	5.00 (-17.33,27.33)	10.06 (-7.21,27.33)	LA+LA														
26.83 (3.68,49.97)	1.76 (-17.99,21.50)	-9.59 (-36.45,17.27)	13.00 (-9.07,35.07)	-15.00 (-36.92,6.92)	-24.80 (-51.14,1.54)	-2.53 (-24.83,19.76)	14.00 (-7.73,35.73)	5.00 (-17.10,27.11)	5.89 (-16.60,28.38)	9.94 (-9.45,29.34)	10.95 (-12.31,34.21)	-19.10 (-52.43,14.23)	24.40 (1.82,46.98)	15.80 (-9.04,40.64)	20.86 (0.45,41.27)	10.80 (-11.25,32.85)	BL+LR													
29.83 (7.72,51.93)	4.76 (-13.76,23.27)	-6.59 (-32.56,19.38)	16.00 (-4.98,36.98)	-12.00 (-32.81,8.81)	-21.80 (-47.23,3.63)	0.47 (-20.74,21.68)	17.00 (-3.62,37.62)	8.00 (-13.01,29.01)	8.89 (-12.53,30.31)	12.94 (-5.20,31.08)	13.95 (-8.27,36.17)	-16.10 (-48.71,16.51)	27.40 (5.89,48.91)	18.80 (-5.07,42.67)	23.86 (4.64,43.08)	13.80 (-7.15,34.75)	3.00 (-20.61,26.61)	LPA+LS+LP												
9.57 (-9.41,28.55)	-15.50 (-30.17,-0.84)	-26.85 (-50.22,-3.48)	-4.26 (-21.92,13.40)	-32.26 (-49.72,-14.80)	-42.06 (-64.83,-19.29)	-19.79 (-37.73,-1.85)	-3.26 (-20.50,13.97)	-12.26 (-29.96,5.44)	-11.37 (-29.55,6.81)	-7.32 (-21.48,6.85)	-6.31 (-25.43,12.81)	-36.36 (-66.94,-5.78)	7.14 (-11.15,25.43)	-1.46 (-22.47,19.55)	3.60 (-11.94,19.14)	-6.46 (-24.09,11.17)	-17.26 (-37.97,3.45)	-20.26 (-39.80,-0.72)	LPA+LA+BAL											
10.42 (-6.49,27.32)	-14.66 (-26.49,-2.82)	-26.00 (-47.72,-4.29)	-3.41 (-18.81,11.99)	-31.41 (-46.59,-16.23)	-41.21 (-62.28,-20.14)	-18.94 (-34.66,-3.22)	-2.41 (-17.33,12.50)	-11.41 (-26.86,4.04)	-10.52 (-26.52,5.48)	-6.47 (-17.70,4.76)	-5.46 (-22.52,11.59)	-35.51 (-64.85,-6.17)	7.99 (-8.14,24.11)	-0.61 (-19.77,18.54)	4.45 (-8.45,17.35)	-5.61 (-20.98,9.75)	-16.41 (-35.24,2.41)	-19.41 (-36.94,-1.88)	0.85 (-12.53,14.23)	BAL+ST+LB										
16.82 (-2.63,36.27)	-8.25 (-23.51,7.01)	-19.60 (-43.36,4.15)	2.99 (-15.17,21.16)	-25.01 (-42.98,-7.03)	-34.80 (-57.97,-11.64)	-12.54 (-30.98,5.90)	3.99 (-13.76,21.74)	-5.01 (-23.21,13.20)	-4.12 (-22.79,14.56)	-0.07 (-14.86,14.73)	0.94 (-18.65,20.53)	-29.11 (-59.99,1.77)	14.39 (-4.39,33.17)	5.79 (-15.65,27.23)	10.85 (-5.25,26.95)	0.79 (-17.34,18.93)	-10.01 (-31.15,11.14)	-13.01 (-33.01,6.99)	7.25 (-9.24,23.74)	6.40 (-7.64,20.45)	LP+LP+PA									
21.20 (2.77,39.63)	-3.88 (-17.84,10.09)	-15.23 (-38.15,7.70)	7.37 (-9.70,24.44)	-20.63 (-37.50,-3.77)	-30.43 (-52.74,-8.11)	-8.16 (-25.52,9.19)	8.37 (-8.26,25.00)	-0.63 (-17.74,16.48)	0.26 (-17.35,17.87)	4.31 (-9.11,17.73)	5.32 (-13.26,23.89)	-24.73 (-54.98,5.51)	18.77 (1.04,36.49)	10.17 (-10.35,30.69)	15.23 (0.35,30.11)	5.17 (-11.87,22.20)	-5.63 (-25.84,14.58)	-8.63 (-27.64,10.38)	11.63 (-3.59,26.85)	10.78 (-1.81,23.36)	4.38 (-11.48,20.23)	EF+LA+X								
17.97 (1.00,34.95)	-7.10 (-19.04,4.85)	-18.45 (-40.22,3.33)	4.15 (-11.34,19.63)	-23.85 (-39.11,-8.59)	-33.65 (-54.78,-12.52)	-11.38 (-27.18,4.42)	5.15 (-9.85,20.14)	-3.85 (-19.38,11.68)	-2.96 (-19.04,13.11)	1.09 (-10.25,12.43)	2.09 (-15.04,19.23)	-27.95 (-57.33,1.43)	15.55 (-0.66,31.75)	6.95 (-12.28,26.17)	12.01 (-1.00,25.01)	1.95 (-13.50,17.39)	-8.85 (-27.75,10.04)	-11.85 (-29.45,5.75)	8.41 (-5.06,21.87)	7.56 (-2.78,17.90)	1.15 (-12.98,15.29)	-3.22 (-15.90,9.45)	LA+ST+X							
20.96 (-1.72,43.63)	-4.11 (-23.31,15.08)	-15.46 (-41.92,10.99)	7.13 (-14.45,28.71)	-20.87 (-42.29,0.55)	-30.67 (-56.59,-4.74)	-8.40 (-30.20,13.40)	8.13 (-13.10,29.36)	-0.87 (-22.48,20.74)	0.02 (-21.99,22.03)	4.07 (-14.76,22.90)	5.08 (-17.71,27.87)	-24.97 (-57.97,8.03)	18.53 (-3.57,40.63)	9.93 (-14.47,34.33)	14.99 (-4.88,34.86)	4.93 (-16.62,26.48)	-5.87 (-30.01,18.27)	-8.87 (-32.01,14.27)	11.39 (-8.79,31.57)	10.54 (-7.70,28.78)	4.14 (-16.49,24.77)	-0.24 (-19.91,19.43)	2.98 (-15.33,21.29)	BAL+LA+BB+X						
20.54 (-0.50,41.57)	-4.53 (-21.76,12.69)	-15.88 (-40.95,9.18)	6.71 (-13.14,26.56)	-21.29 (-40.96,-1.62)	-31.09 (-55.59,-6.58)	-8.82 (-28.92,11.28)	7.71 (-11.76,27.18)	-1.29 (-21.17,18.60)	-0.40 (-20.71,19.91)	3.65 (-13.17,20.47)	4.66 (-16.50,25.82)	-25.39 (-57.29,6.51)	18.11 (-2.30,38.52)	9.51 (-13.37,32.39)	14.57 (-3.41,32.55)	4.51 (-15.31,24.33)	-6.29 (-28.90,16.32)	-9.29 (-30.83,12.25)	10.97 (-7.35,29.29)	10.12 (-6.04,26.28)	3.72 (-15.09,22.53)	-0.66 (-18.41,17.10)	2.56 (-13.67,18.80)	-0.42 (-22.54,21.70)	LR+PF+X					
8.19 (-9.99,26.37)	-16.88 (-30.45,-3.31)	-28.23 (-50.95,-5.51)	-5.64 (-22.42,11.15)	-33.64 (-50.21,-17.06)	-43.43 (-65.53,-21.34)	-21.17 (-38.24,-4.09)	-4.64 (-20.97,11.70)	-13.64 (-30.46,3.19)	-12.75 (-30.08,4.59)	-8.69 (-21.76,4.37)	-7.69 (-26.00,10.63)	-37.74 (-67.82,-7.65)	5.76 (-11.69,23.21)	-2.84 (-23.12,17.44)	2.22 (-12.29,16.73)	-7.84 (-24.59,8.91)	-18.64 (-38.61,1.33)	-21.64 (-40.39,-2.88)	-1.38 (-16.35,13.59)	-2.23 (-14.43,9.98)	-8.63 (-24.17,6.92)	-13.00 (-27.30,1.29)	-9.78 (-22.09,2.53)	-12.77 (-32.19,6.65)	-12.35 (-29.83,5.13)	BAL+LA+LR+X				
17.45 (-0.52,35.42)	-7.62 (-20.94,5.69)	-18.97 (-41.52,3.58)	3.62 (-12.94,20.18)	-24.38 (-40.73,-8.02)	-34.17 (-56.10,-12.24)	-11.91 (-28.76,4.95)	4.62 (-11.48,20.73)	-4.38 (-20.98,12.23)	-3.49 (-20.60,13.63)	0.57 (-12.21,13.34)	1.57 (-16.54,19.68)	-28.48 (-58.44,1.48)	15.02 (-2.21,32.26)	6.42 (-13.68,26.52)	11.48 (-2.79,25.75)	1.42 (-15.10,17.95)	-9.38 (-29.16,10.41)	-12.38 (-30.93,6.18)	7.88 (-6.80,22.57)	7.03 (-4.86,18.93)	0.63 (-14.67,15.94)	-3.74 (-17.70,10.21)	-0.52 (-12.52,11.47)	-3.51 (-22.74,15.72)	-3.09 (-20.35,14.18)	9.26 (-4.39,22.91)	LA+LR+ST+BL+BBR+X			
22.76 (2.91,42.61)	-2.32 (-18.07,13.44)	-13.66 (-37.74,10.41)	8.93 (-9.66,27.51)	-19.07 (-37.47,-0.67)	-28.87 (-52.36,-5.37)	-6.60 (-25.45,12.25)	9.93 (-8.25,28.11)	0.93 (-17.69,19.56)	1.82 (-17.26,20.90)	5.87 (-9.44,21.18)	6.88 (-13.10,26.86)	-23.17 (-54.30,7.96)	20.33 (1.14,39.52)	11.73 (-10.07,33.53)	16.79 (0.21,33.36)	6.73 (-11.83,25.28)	-4.07 (-25.58,17.44)	-7.07 (-27.45,13.31)	13.19 (-3.75,30.13)	12.34 (-2.24,26.92)	5.94 (-11.54,23.41)	1.56 (-14.76,17.88)	4.78 (-9.88,19.45)	1.80 (-19.20,22.80)	2.22 (-17.00,21.44)	14.57 (-1.47,30.60)	5.31 (-10.49,21.10)	VSL#3		
16.73 (-0.23,33.69)	-8.34 (-20.24,3.56)	-19.69 (-41.45,2.07)	2.90 (-12.56,18.36)	-25.10 (-40.33,-9.86)	-34.89 (-56.00,-13.78)	-12.63 (-28.40,3.15)	3.90 (-11.07,18.88)	-5.10 (-20.60,10.41)	-4.21 (-20.26,11.85)	-0.15 (-11.47,11.16)	0.85 (-16.26,17.96)	-29.20 (-58.56,0.17)	14.30 (-1.88,30.48)	5.70 (-13.50,24.90)	10.76 (-2.20,23.72)	0.70 (-14.72,16.13)	-10.10 (-28.97,8.77)	-13.10 (-30.68,4.48)	7.16 (-6.30,20.63)	6.31 (-3.99,16.62)	-0.09 (-14.20,14.02)	-4.47 (-17.17,8.24)	-1.24 (-11.67,9.19)	-4.23 (-22.52,14.06)	-3.81 (-20.02,12.41)	8.54 (-3.72,20.80)	-0.72 (-12.70,11.26)	-6.03 (-20.67,8.62)	LP+LC+BL+ST+LA+X	
9.83 (-5.46,25.11)	'-15.24 (-24.62,-5.87)	-26.59 (-47.07,-6.11)	-4.00 (-17.60,9.60)	-32.00 (-45.35,-18.65)	-41.80 (-61.59,-22.00)	-19.53 (-33.49,-5.57)	-3.00 (-16.05,10.05)	-12.00 (-25.65,1.66)	-11.11 (-25.38,3.17)	-7.06 (-15.66,1.55)	-6.05 (-21.50,9.40)	-36.10 (-64.53,-7.67)	7.40 (-7.01,21.81)	-1.20 (-18.94,16.54)	3.86 (-6.83,14.55)	-6.20 (-19.76,7.36)	-17.00 (-34.38,0.38)	-20.00 (-35.97,-4.03)	0.26 (-11.00,11.52)	-0.59 (-7.81,6.64)	-6.99 (-19.03,5.05)	-11.37 (-21.68,-1.06)	-8.14 (-15.54,-0.75)	-11.13 (-27.88,5.62)	-10.71 (-25.16,3.75)	1.64 (-8.19,11.47)	-7.62 (-17.07,1.82)	-12.93 (-25.59,-0.26)	-6.90 (-14.25,0.45)	Placebo

7. Abdominal bloating score

L. plantarum CCFM8610																								
-11.58 (-36.83,13.66)	S. cerevisiae CNCM I-3856																							
2.78 (-27.51,33.06)	14.36 (-18.89,47.61)	L. plantarum Apsulloc 331261																						
-5.13 (-25.85,15.59)	6.45 (-18.40,31.30)	-7.91 (-37.87,22.05)	L. plantarum 299v																					
-11.92 (-32.78,8.94)	-0.34 (-25.31,24.63)	-14.70 (-44.75,15.35)	-6.79 (-27.17,13.59)	B. coagulans Unique IS2																				
10.75 (-29.73,51.23)	22.33 (-20.41,65.07)	7.97 (-37.93,53.87)	15.88 (-24.36,56.12)	22.67 (-17.64,62.98)	B. coagulans MTCC 5856																			
-8.19 (-28.44,12.06)	3.39 (-21.07,27.85)	-10.97 (-40.60,18.66)	-3.06 (-22.82,16.70)	3.73 (-16.17,23.63)	-18.94 (-58.94,21.06)	L. acidophilus DDS-1																		
-18.82 (-38.21,0.56)	-7.24 (-30.99,16.51)	-21.60 (-50.65,7.45)	-13.69 (-32.56,5.18)	-6.90 (-25.92,12.12)	-29.57 (-69.14,10.00)	-10.63 (-28.98,7.72)	L. acidophilus ATCC 700396																	
-13.49 (-33.82,6.84)	-1.91 (-26.43,22.61)	-16.27 (-45.96,13.42)	-8.36 (-28.20,11.48)	-1.57 (-21.55,18.41)	-24.24 (-64.28,15.80)	-5.30 (-19.17,8.57)	5.33 (-13.11,23.77)	B. lactis UABla-12																
-8.09 (-26.73,10.55)	3.49 (-19.66,26.64)	-10.87 (-39.43,17.69)	-2.96 (-21.07,15.15)	3.83 (-14.43,22.10)	-18.84 (-58.05,20.37)	0.10 (-17.46,17.67)	10.73 (-5.83,27.29)	5.40 (-12.26,23.06)	B. bifidum MIMBb75															
-18.97 (-37.70,-0.23)	-7.38 (-30.61,15.84)	-21.74 (-50.37,6.88)	-13.83 (-32.04,4.37)	-7.04 (-25.40,11.32)	-29.71 (-68.97,9.54)	-10.77 (-28.44,6.89)	-0.14 (-16.81,16.52)	-5.47 (-23.23,12.28)	-10.87 (-26.67,4.92)	C. butyricum CGMCC0313.1														
-21.32 (-41.44,-1.20)	-9.74 (-34.09,14.61)	-24.10 (-53.65,5.45)	-16.19 (-35.82,3.44)	-9.40 (-29.17,10.37)	-32.07 (-72.00,7.86)	-13.13 (-32.26,6.00)	-2.50 (-20.71,15.71)	-7.83 (-27.04,11.38)	-13.23 (-30.65,4.19)	-2.36 (-19.87,15.16)	B. infantis 35624													
-18.85 (-37.54,-0.17)	-7.27 (-30.46,15.91)	-21.63 (-50.22,6.96)	-13.72 (-31.88,4.43)	-6.93 (-25.24,11.38)	-29.60 (-68.83,9.63)	-10.66 (-28.27,6.95)	-0.03 (-16.64,16.58)	-5.36 (-23.07,12.34)	-10.76 (-26.50,4.98)	0.11 (-15.74,15.96)	2.47 (-15.00,19.93)	S. boulardii CNCM I-475												
14.08 (-13.32,41.48)	25.66 (-4.99,56.30)	11.30 (-23.62,46.21)	19.21 (-7.83,46.25)	26.00 (-1.15,53.15)	3.33 (-40.72,47.38)	22.27 (-4.41,48.95)	32.90 (6.87,58.93)	27.57 (0.83,54.31)	22.17 (-3.32,47.65)	33.04 (7.49,58.59)	35.40 (8.81,61.98)	32.93 (7.42,58.45)	BL+LR											
-25.59 (-44.00,-7.17)	-14.01 (-36.97,8.96)	-28.37 (-56.78,0.05)	-20.46 (-38.33,-2.58)	-13.67 (-31.70,4.37)	-36.34 (-75.44,2.76)	-17.40 (-34.72,-0.08)	-6.77 (-23.07,9.54)	-12.10 (-29.51,5.32)	-17.50 (-32.91,-2.08)	-6.62 (-22.15,8.91)	-4.27 (-21.44,12.91)	-6.73 (-22.20,8.74)	-39.67 (-64.98,-14.35)	LPA+LA+BAL										
-19.01 (-35.59,-2.43)	-7.43 (-28.95,14.09)	-21.79 (-49.05,5.47)	-13.88 (-29.85,2.10)	-7.09 (-23.24,9.07)	-29.76 (-68.03,8.51)	-10.82 (-26.17,4.54)	-0.19 (-14.38,14.01)	-5.52 (-20.98,9.94)	-10.92 (-24.08,2.25)	-0.04 (-13.34,13.26)	2.31 (-12.88,17.50)	-0.15 (-13.38,13.08)	-33.09 (-57.10,-9.07)	6.58 (-6.26,19.42)	BAL+ST+LB									
-15.32 (-34.88,4.23)	-3.74 (-27.63,20.15)	-18.10 (-47.27,11.07)	-10.19 (-29.24,8.86)	-3.40 (-22.60,15.80)	-26.07 (-65.72,13.58)	-7.13 (-25.66,11.40)	3.50 (-14.08,21.08)	-1.83 (-20.45,16.79)	-7.23 (-23.99,9.53)	3.64 (-13.22,20.51)	6.00 (-12.39,24.39)	3.53 (-13.28,20.35)	-29.40 (-55.56,-3.24)	10.27 (-6.24,26.78)	3.69 (-10.75,18.12)	EF+LA+X								
-10.62 (-29.57,8.32)	0.96 (-22.43,24.35)	-13.40 (-42.16,15.36)	-5.49 (-23.91,12.93)	1.30 (-17.27,19.87)	-21.37 (-60.72,17.98)	-2.43 (-20.31,15.46)	8.20 (-8.70,25.10)	2.87 (-15.11,20.85)	-2.53 (-18.58,13.52)	8.34 (-7.81,24.50)	10.70 (-7.04,28.44)	8.23 (-7.86,24.33)	-24.70 (-50.40,1.01)	14.97 (-0.81,30.75)	8.39 (-5.21,21.98)	4.70 (-12.40,21.80)	LA+ST+X							
-11.92 (-34.92,11.07)	-0.34 (-27.12,26.44)	-14.70 (-46.28,16.88)	-6.79 (-29.36,15.78)	0.00 (-22.69,22.69)	-22.67 (-64.12,18.79)	-3.73 (-25.86,18.40)	6.90 (-14.44,28.24)	1.57 (-20.63,23.77)	-3.83 (-24.50,16.84)	7.04 (-13.71,27.80)	9.40 (-12.62,31.41)	6.93 (-13.78,27.64)	-26.00 (-54.82,2.82)	13.67 (-6.80,34.13)	7.09 (-11.74,25.92)	3.40 (-18.10,24.90)	-1.30 (-22.25,19.65)	BAL+LA+BB+X						
-9.21 (-29.78,11.35)	2.37 (-22.35,27.09)	-11.99 (-41.84,17.86)	-4.08 (-24.16,16.00)	2.71 (-17.51,22.93)	-19.96 (-60.12,20.20)	-1.02 (-20.61,18.57)	9.61 (-9.09,28.31)	4.28 (-15.39,23.95)	-1.12 (-19.05,16.81)	9.75 (-8.27,27.78)	12.11 (-7.35,31.57)	9.64 (-8.33,27.62)	-23.29 (-50.21,3.63)	16.38 (-1.31,34.07)	9.80 (-5.97,25.57)	6.11 (-12.77,24.99)	1.41 (-16.83,19.65)	2.71 (-19.71,25.13)	LR+PF+X					
-18.65 (-36.53,-0.78)	-7.07 (-29.61,15.46)	-21.43 (-49.50,6.64)	-13.52 (-30.84,3.80)	-6.73 (-24.21,10.75)	-29.40 (-68.25,9.45)	-10.46 (-27.21,6.29)	0.17 (-15.53,15.86)	-5.16 (-22.01,11.68)	-10.56 (-25.33,4.21)	0.31 (-14.58,15.20)	2.67 (-13.93,19.27)	0.20 (-14.62,15.02)	-32.73 (-57.66,-7.80)	6.93 (-7.55,21.42)	0.35 (-11.71,12.42)	-3.33 (-19.24,12.58)	-8.03 (-23.18,7.12)	-6.73 (-26.72,13.25)	-9.44 (-26.57,7.69)	BAL+LA+LR+X				
-16.94 (-34.39,0.50)	-5.36 (-27.56,16.83)	-19.72 (-47.52,8.07)	-11.81 (-28.69,5.06)	-5.02 (-22.06,12.02)	-27.69 (-66.35,10.96)	-8.75 (-25.04,7.53)	1.88 (-13.32,17.08)	-3.45 (-19.84,12.93)	-8.85 (-23.10,5.39)	2.02 (-12.34,16.39)	4.38 (-11.75,20.51)	1.91 (-12.39,16.21)	-31.02 (-55.64,-6.40)	8.64 (-5.30,22.58)	2.06 (-9.34,13.47)	-1.62 (-17.04,13.80)	-6.32 (-20.96,8.31)	-5.02 (-24.62,14.58)	-7.73 (-24.41,8.95)	1.71 (-11.52,14.94)	LA+LR+ST+BL+BBR+X			
-6.21 (-23.40,10.97)	5.37 (-16.62,27.36)	-8.99 (-36.62,18.64)	-1.08 (-17.68,15.52)	5.71 (-11.06,22.48)	-16.96 (-55.50,21.57)	1.98 (-14.03,17.98)	12.61 (-2.29,27.50)	7.28 (-8.83,23.39)	1.88 (-12.04,15.80)	12.75 (-1.29,26.80)	15.11 (-0.74,30.95)	12.64 (-1.34,26.62)	-20.29 (-44.73,4.14)	19.37 (5.76,32.99)	12.79 (1.79,23.80)	9.11 (-6.01,24.23)	4.41 (-9.91,18.73)	5.71 (-13.66,25.07)	3.00 (-13.41,19.40)	12.44 (-0.44,25.32)	10.73 (-1.54,23.00)	VSL#3		
-14.21 (-30.73,2.31)	-2.63 (-24.10,18.85)	-16.99 (-44.21,10.24)	-9.08 (-25.00,6.84)	-2.29 (-18.38,13.80)	-24.96 (-63.20,13.29)	-6.02 (-21.31,9.27)	4.61 (-9.52,18.74)	-0.72 (-16.12,14.68)	-6.12 (-19.22,6.98)	4.75 (-8.47,17.98)	7.11 (-8.02,22.24)	4.64 (-8.51,17.79)	-28.29 (-52.26,-4.31)	11.38 (-1.41,24.17)	4.80 (-5.16,14.76)	1.11 (-13.26,15.48)	-3.59 (-17.11,9.93)	-2.29 (-21.07,16.49)	-5.00 (-20.71,10.71)	4.44 (-7.47,16.36)	2.73 (-8.61,14.08)	-8.00 (-18.92,2.92)	LP+LC+BL+ST+LA+X	
-19.92 (-34.91,-4.94)	-8.34 (-28.66,11.97)	-22.70 (-49.02,3.62)	-14.79 (-29.11,-0.48)	-8.00 (-22.51,6.51)	-30.67 (-68.28,6.94)	-11.73 (-25.35,1.89)	-1.10 (-13.40,11.20)	-6.43 (-20.17,7.31)	-11.83 (-22.93,-0.74)	-0.96 (-12.21,10.29)	1.40 (-12.03,14.83)	-1.07 (-12.24,10.10)	-34.00 (-56.94,-11.06)	5.67 (-5.04,16.37)	-0.91 (-8.01,6.18)	-4.60 (-17.17,7.97)	-9.30 (-20.90,2.29)	-8.00 (-25.44,9.44)	-10.71 (-24.80,3.37)	-1.27 (-11.02,8.48)	-2.98 (-11.91,5.96)	-13.71 (-22.12,-5.30)	-5.71 (-12.67,1.25)	Placebo

8. Bowel movement frequency (per week) in IBS-D

L. paracasei HA-196										
1.98 (-3.11,7.08)	L. paracasei B21060									
-3.13 (-6.22,-0.03)	-5.11 (-10.68,0.46)	S. cerevisiae CNCM I-3856								
-1.07 (-4.19,2.05)	-3.06 (-8.65,2.53)	2.05 (-1.80,5.90)	L. plantarum Apsulloc 331261							
-1.01 (-2.75,0.73)	-3.00 (-7.95,1.96)	2.11 (-0.73,4.96)	0.06 (-2.81,2.94)	B. coagulans GBI-306086						
-1.17 (-2.60,0.26)	-3.16 (-8.26,1.94)	1.95 (-1.14,5.05)	-0.10 (-3.22,3.02)	-0.16 (-1.90,1.58)	B. longum R0175					
-2.61 (-4.21,-1.01)	-4.59 (-9.50,0.31)	0.52 (-2.25,3.28)	-1.54 (-4.32,1.25)	-1.60 (-2.63,-0.56)	-1.44 (-3.03,0.16)	C. butyricum				
-5.37 (-8.47,-2.27)	-7.36 (-12.93,-1.78)	-2.25 (-6.08,1.58)	-4.30 (-8.15,-0.45)	-4.36 (-7.21,-1.51)	-4.20 (-7.30,-1.10)	-2.76 (-5.53,0.00)	S. boulardii			
0.82 (-1.02,2.67)	-1.16 (-6.15,3.83)	3.95 (1.04,6.86)	1.90 (-1.04,4.83)	1.83 (0.45,3.22)	2.00 (0.15,3.84)	3.43 (2.23,4.63)	6.19 (3.28,9.11)	EF+LA+X		
-2.43 (-5.87,1.02)	-4.41 (-10.18,1.36)	0.70 (-3.41,4.81)	-1.35 (-5.48,2.78)	-1.41 (-4.63,1.81)	-1.25 (-4.69,2.19)	0.18 (-2.96,3.33)	2.95 (-1.17,7.06)	-3.25 (-6.52,0.03)	LP+LC+BL+ST+LA+X	
-3.13 (-4.63,-1.62)	-5.11 (-9.98,-0.24)	-0.00 (-2.71,2.71)	-2.05 (-4.79,0.68)	-2.11 (-3.00,-1.23)	-1.95 (-3.45,-0.45)	-0.52 (-1.06,0.03)	2.25 (-0.46,4.96)	-3.95 (-5.02,-2.88)	-0.70 (-3.80,2.40)	Placebo

9. Bowel movement frequency (per week) in IBS-C

L. paracasei HA-196							
-0.08 (-7.77,7.60)	S. cerevisiae CNCM I-3856						
0.02 (-7.62,7.66)	0.10 (-7.60,7.80)	B. coagulans Unique IS2					
0.49 (-5.06,6.04)	0.57 (-7.23,8.37)	0.47 (-7.28,8.23)	B. longum R0175				
-1.35 (-9.42,6.72)	-1.27 (-9.39,6.85)	-1.37 (-9.45,6.71)	-1.84 (-10.02,6.34)	B. bifidum MIMBb75			
1.25 (-5.06,7.57)	1.33 (-5.06,7.73)	1.23 (-5.11,7.58)	0.76 (-5.70,7.23)	2.60 (-4.25,9.46)	BAL+ST+LB		
-0.30 (-8.67,8.06)	-0.22 (-8.64,8.19)	-0.32 (-8.70,8.06)	-0.80 (-9.27,7.68)	1.05 (-7.72,9.82)	-1.56 (-8.76,5.65)	LA+ST+X	
1.32 (-4.08,6.72)	1.40 (-4.07,6.87)	1.30 (-4.11,6.71)	0.83 (-4.73,6.39)	2.67 (-3.33,8.67)	0.07 (-3.25,3.38)	1.62 (-4.77,8.02)	Placebo

10. Bristol stool form scale in IBS-D

L. paracasei HA-196												
1.00 (0.09,1.91)	S. cerevisiae CNCM I-3856											
3.03 (0.92,5.13)	2.03 (0.05,4.01)	B. coagulans MTCC 5856										
-0.19 (-1.10,0.72)	-1.19 (-1.99,-0.38)	-3.22 (-5.28,-1.15)	B. longum R0175									
-0.05 (-0.90,0.80)	-1.04 (-1.49,-0.60)	-3.08 (-5.03,-1.13)	0.14 (-0.60,0.88)	B. bifidum MIMBb75								
-0.14 (-1.04,0.76)	-1.13 (-1.67,-0.60)	-3.16 (-5.14,-1.19)	0.05 (-0.74,0.85)	-0.09 (-0.51,0.33)	C. butyricum							
-0.75 (-1.67,0.17)	-1.74 (-2.31,-1.17)	-3.77 (-5.76,-1.79)	-0.56 (-1.38,0.26)	-0.70 (-1.17,-0.23)	-0.61 (-1.17,-0.06)	S. boulardii						
0.55 (-0.57,1.67)	-0.45 (-1.30,0.41)	-2.48 (-4.56,-0.39)	0.74 (-0.30,1.78)	0.60 (-0.19,1.39)	0.69 (-0.16,1.54)	1.30 (0.43,2.17)	BL+LR					
0.25 (-0.61,1.11)	-0.74 (-1.21,-0.28)	-2.78 (-4.73,-0.82)	0.44 (-0.31,1.19)	0.30 (-0.04,0.64)	0.39 (-0.06,0.84)	1.00 (0.51,1.49)	-0.30 (-1.11,0.51)	LP+LP+PA				
0.45 (-0.58,1.48)	-0.54 (-1.28,0.19)	-2.58 (-4.61,-0.54)	0.64 (-0.30,1.58)	0.50 (-0.16,1.16)	0.59 (-0.14,1.31)	1.20 (0.45,1.95)	-0.10 (-1.09,0.89)	0.20 (-0.48,0.88)	LA+LR+ST+BL+BBR+X			
-0.05 (-1.08,0.98)	-1.04 (-1.77,-0.32)	-3.08 (-5.11,-1.04)	0.14 (-0.80,1.08)	0.00 (-0.65,0.65)	0.09 (-0.63,0.81)	0.70 (-0.05,1.44)	-0.60 (-1.58,0.38)	-0.30 (-0.97,0.37)	-0.50 (-1.38,0.38)	VSL#3		
-0.61 (-1.54,0.32)	-1.60 (-2.19,-1.02)	-3.64 (-5.62,-1.65)	-0.42 (-1.25,0.41)	-0.56 (-1.04,-0.08)	-0.47 (-1.04,0.09)	0.14 (-0.46,0.74)	-1.16 (-2.04,-0.28)	-0.86 (-1.36,-0.36)	-1.06 (-1.82,-0.30)	-0.56 (-1.31,0.19)	LP+LC+BL+ST+LA+X	
-0.25 (-1.07,0.57)	-1.24 (-1.63,-0.86)	-3.28 (-5.21,-1.34)	-0.06 (-0.77,0.65)	-0.20 (-0.41,0.01)	-0.11 (-0.48,0.25)	0.50 (0.08,0.92)	-0.80 (-1.57,-0.03)	-0.50 (-0.76,-0.24)	-0.70 (-1.32,-0.08)	-0.20 (-0.82,0.42)	0.36 (-0.07,0.79)	Placebo

11. Bristol stool form scale in IBS-C.

L. paracasei HA-196				
-0.14 (-2.02,1.74)	S. cerevisiae CNCM I-3856			
0.90 (-0.90,2.70)	1.04 (-1.15,3.24)	B. longum R0175		
0.69 (-0.83,2.21)	0.83 (-0.69,2.35)	-0.21 (-2.11,1.68)	BAL+ST+LB	
1.03 (-0.30,2.36)	1.17 (-0.16,2.50)	0.13 (-1.62,1.88)	0.34 (-0.39,1.07)	Placebo

Table S5. The surface under the cumulative ranking curve (SUCRA) values.

1. IBS-SSS

Treatment	SUCRA
L. acidophilus DDS-1	92.9
BL+LR	91.6
LC+LP+BAL	90.9
LP+LC+BL+ST+LA+X	85.2
B. lactis UABla-12	75.8
EF+LA+X	66.2
B. bifidum MIMBb75	62.6
C. butyricum	54.2
BAL+LA+BB+X	51.8
L. acidophilus ATCC 700396	38.1
L. plantarum 299v	38
L. plantarum CCFM8610	31.4
L. casei LCR35	31.3
L. paracasei HA-196	31.1
Placebo	30.2
B. longum R0175	10.5
BAL+ST+LB	10.1
LR+PF+X	8

2. IBS-QOL

Treatment	SUCRA
LP+LC+BL+ST+LA+X	100
S. boulardii	70.7
C. butyricum	64.2
LC+LP+BAL	55.3
LA+LR+ST+BL+BBR+X	54
EF+LA+X	39.8
BAL+ST+LB	39.7
LPA+LA+BAL	30.7
L. acidophilus ATCC 700396	26.1
Placebo	19.5

3. HADS-total score

Treatment	SUCRA
L. acidophilus ATCC 700396	78.3
B. longum R0175	77.9
Placebo	26.9
L. paracasei HA-196	17

4. HADS-anxiety

Treatment	SUCRA
B. longum NCC3001	93
L. acidophilus ATCC 700396	59.5
Placebo	44
LPA+LA+BAL	3.4

5. HADS-depression

Treatment	SUCRA
B. longum NCC3001	99.1
L. paracasei CNCM I-1572	57.3
L. acidophilus ATCC 700396	45.2
LPA+LA+BAL	28.1
Placebo	20.2

6. Abdominal pain score

Treatment	SUCRA
B. coagulans MTCC 5856	96.9
B. coagulans Unique IS2	92.6
L. gasseri BNR17	91.3
L. plantarum Apsulloc 331261	85.4
L. acidophilus DDS-1	78.2
LPA+LS+LP	77.6
BL+LR	70.8
S. cerevisiae CNCM I-3856	70.5
VSL#3	63
L. acidophilus SDC 2012,2013	60
EF+LA+X	59.6
B. lactis UABla-12	57.5
BAL+LA+BB+X	56.7
LR+PF+X	56.2
LA+ST+X	49.7
LA+LR+ST+BL+BBR+X	47.4
B. bifidum MIMBb75	45.6
LP+LC+BL+ST+LA+X	45.2
LP+LP+PA	45
LA+LA	43
C. butyricum CGMCC0313.1	42.1
L. plantarum 299v	36.4
L. acidophilus ATCC 700396	33.3
L. casei LCR35	29.6
BAL+ST+LB	23.9
LPA+LA+BAL	22.9
Placebo	20.6
BAL+LA+LR+X	18.5
S. boulardii CNCM I-475	13.8
B. infantis 35624	9.7
L. plantarum CCFM8610	6.9

7. Abdominal bloating score

Treatment	SUCRA
BL+LR	94.5
B. coagulans MTCC 5856	85
L. plantarum CCFM8610	82.6
L. plantarum Apsulloc 331261	80.3
L. plantarum 299v	72
VSL#3	72
B. bifidum MIMBb75	65.5
L. acidophilus DDS-1	64.2
LR+PF+X	60.7
LA+ST+X	57.3
S. cerevisiae CNCM I-3856	52.2
B. coagulans Unique IS2	52.2
BAL+LA+BB+X	51.4
B. lactis UABla-12	46.5
LP+LC+BL+ST+LA+X	45.4
EF+LA+X	40.9
LA+LR+ST+BL+BBR+X	35
L. acidophilus ATCC 700396	29
BAL+LA+LR+X	28.5
S. boulardii CNCM I-475	28.2
C. butyricum CGMCC0313.1	28
BAL+ST+LB	26.4
B. infantis 35624	21.7
Placebo	21.3
LPA+LA+BAL	9.2

8. Bowel movement frequency (per week) in IBS-D

Treatment	SUCRA
EF+LA+X	89.7
L. paracasei B21060	89.2
L. paracasei HA-196	78.7
B. coagulans GBI-306086	60.5
L. plantarum Apsulloc 331261	58.7
B. longum R0175	57.1
LP+LC+BL+ST+LA+X	36.5
C. butyricum	32.5
S. cerevisiae CNCM I-3856	24.7
Placebo	19.1
S. boulardii	3.3

9. Bowel movement frequency (per week) in IBS-C

Treatment	SUCRA
B. bifidum MIMBb75	67.3
LA+ST+X	56.5
S. cerevisiae CNCM I-3856	53.7
L. paracasei HA-196	53.6
B. coagulans Unique IS2	53.1
B. longum R0175	47.4
BAL+ST+LB	35.9

10. Bristol stool form scale in IBS-D

Treatment	SUCRA
B. coagulans MTCC 5856	99.6
S. cerevisiae CNCM I-3856	89.7
BL+LR	75
LA+LR+ST+BL+BBR+X	72.2
LP+LP+PA	65.1
L. paracasei HA-196	46.6
B. bifidum MIMBb75	44.9
VSL#3	43.8
C. butyricum	37.5
B. longum R0175	34.5
Placebo	27.2
LP+LC+BL+ST+LA+X	9.5
S. boulardii	4.5

11. Bristol stool form scale in IBS-C

Treatment	SUCRA
L. paracasei HA-196	75.4
S. cerevisiae CNCM I-3856	80.1
B. longum R0175	32.8
BAL+ST+LB	43.3
Placebo	18.4

Table S6 Results of loop specific analysis, node-splitting method and design-by-treatment interaction inconsistency model.

Outcomes	loop specific analysis	node-splitting method	design-by-treatment interaction inconsistency model
IBS-SSS	NA (Loops were formed only by multi-arm trial(s) - Consistent by definition)	p>0·05 (all sides)	NA*
IBS-QOL	NA (No loops found)	p>0·05 (all sides)	NA*
HADS-total score	NA (Loops were formed only by multi-arm trial(s) - Consistent by definition)	p>0·05 (all sides)	NA*
HADS-anxiety	NA (No loops found)	p>0·05 (all sides)	NA*
HADS-depression	NA (No loops found)	p>0·05 (all sides)	NA*
Abdominal pain score	NA (Loops were formed only by multi-arm trial(s) - Consistent by definition)	p>0·05 (all sides)	NA*
Abdominal bloating score	NA (Loops were formed only by multi-arm trial(s) - Consistent by definition)	p>0·05 (all sides)	NA*
Bowel movement frequency in IBS-D	NA (Loops were formed only by multi-arm trial(s) - Consistent by definition)	p>0·05 (all sides)	NA*
Bowel movement frequency in IBS-C	NA (Loops were formed only by multi-arm trial(s) - Consistent by definition)	p>0·05 (all sides)	NA*
Bristol stool form scale in IBS-D	NA (Loops were formed only by multi-arm trial(s) - Consistent by definition)	p>0·05 (all sides)	NA*
Bristol stool form scale in IBS-C	NA (Loops were formed only by multi-arm trial(s) - Consistent by definition)	p>0·05 (all sides)	NA*
Straining	NA (No loops found)	p>0·05 (all sides)	NA*
Urgency	NA (No loops found)	p>0·05 (all sides)	NA*

*The design-by-treatment interaction inconsistency model indicated that there is no source of inconsistency.

Table S7. GRADE-based assessment of the quality of evidence for each outcome

1. IBS-SSS

Comparison(vs. placebo)	Confidence	Downgrading due to
L. acidophilus DDS-1	Moderate	Inconsistency
BL+LR	Moderate	Study limitation
LC+LP+BAL	Moderate	Study limitation
LP+LC+BL+ST+LA+X	Moderate	Inconsistency
B. lactis UABla-12	Moderate	Inconsistency
EF+LA+X	Moderate	Study limitation
B. bifidum MIMBb75	High	NA
C. butyricum	High	NA
BAL+LA+BB+X	Moderate	Study limitation
L. acidophilus ATCC 700396	Moderate	Inconsistency
L. plantarum 299v	Moderate	Study limitation
L. plantarum CCFM8610	High	NA
L. casei LCR35	Low	Study limitation; Imprecision
L. paracasei HA-196	High	NA
B. longum R0175	High	NA
BAL+ST+LB	Moderate	Study limitation
LR+PF+X	Low	Study limitation; Imprecision
Ranking	Moderate	Study limitation

2. IBS-QOL

Comparison(vs. placebo)	Confidence	Downgrading due to
LP+LC+BL+ST+LA+X	Moderate	Study limitation
S. boulardii	Moderate	Study limitation
C. butyricum	High	NA
LC+LP+BAL	Moderate	Study limitation
LA+LR+ST+BL+BBR+X	Moderate	Study limitation
EF+LA+X	Moderate	Inconsistency
BAL+ST+LB	Moderate	Study limitation
LPA+LA+BAL	Low	Study limitation; Imprecision
L. acidophilus ATCC 700396	Moderate	Study limitation
Ranking	Moderate	Study limitation

3. HADS-total score

Comparison(vs. placebo)	Confidence	Downgrading due to
L. acidophilus ATCC 700396	Moderate	Study limitation
B. longum R0175	High	NA
L. paracasei HA-196	High	NA
Ranking	Moderate	Study limitation

4. HADS-anxiety

Comparison(vs. placebo)	Confidence	Downgrading due to
B. longum NCC3001	High	NA
L. acidophilus ATCC 700396	Moderate	Study limitation
LPA+LA+BAL	Moderate	Study limitation
Ranking	Moderate	Study limitation

5. HADS-depression

Comparison(vs. placebo)	Confidence	Downgrading due to
B. longum NCC3001	High	NA
L. paracasei CNCM I-1572	High	NA
L. acidophilus ATCC 700396	Moderate	Study limitation
LPA+LA+BAL	Low	Study limitation; Imprecision
Ranking	Low	Study limitation

6. Abdominal pain score

Comparison(vs. placebo)	Confidence	Downgrading due to
B. coagulans MTCC 5856	Moderate	Study limitation
B. coagulans Unique IS2	Moderate	Study limitation
L. gasseri BNR17	Moderate	Study limitation
L. plantarum Apsulloc 331261	Moderate	Study limitation
L. acidophilus DDS-1	Moderate	inconsistency
LPA+LS+LP	Low	Study limitation; inconsistency
BL+LR	Low	Study limitation; inconsistency
S. cerevisiae CNCM I-3856	Moderate	Study limitation
VSL#3	Low	Study limitation; inconsistency
L. acidophilus SDC 20122013	Moderate	inconsistency
EF+LA+X	Moderate	Study limitation
B. lactis UABla-12	High	NA
BAL+LA+BB+X	Low	Study limitation; Imprecision
LR+PF+X	Moderate	Study limitation
LA+ST+X	Moderate	Study limitation
LA+LR+ST+BL+BBR+X	Moderate	Study limitation
B. bifidum MIMBb75	Low	Study limitation
LP+LP+PA	Low	Study limitation; inconsistency
LP+LC+BL+ST+LA+X	Low	Study limitation; inconsistency
LA+LA	Low	Study limitation; inconsistency
C. butyricum	High	NA
L. plantarum 299v	Moderate	Study limitation
L. acidophilus ATCC 700396	Moderate	Inconsistency
L. casei LCR35	Moderate	Study limitation
BAL+ST+LB	Low	Study limitation; inconsistency
LPA+LA+BAL	Low	Study limitation; inconsistency
BAL+LA+LR+X	Moderate	Study limitation
S. boulardii	Low	Study limitation; Imprecision
B. infantis 35624	Low	Study limitation
L. plantarum CCFM8610	Moderate	Study limitation
Ranking	Moderate	Study limitation

7. Abdominal bloating score

Comparison	Confidence	Downgrading due to
BL+LR	Moderate	Study limitation
B. coagulans MTCC 5856	Moderate	Study limitation

L. plantarum CCFM8610	Moderate	Study limitation
L. plantarum Apsulloc 331261	Moderate	Study limitation
VSL#3	Low	Study limitation
L. plantarum 299v	Moderate	Study limitation
B. bifidum MIMBb75	Moderate	Study limitation
L. acidophilus DDS-1	High	NA
LR+PF+X	Moderate	Study limitation
LA+ST+X	Moderate	Study limitation
B. coagulans Unique IS2	Moderate	Study limitation
S. cerevisiae CNCM I-3856	Moderate	Study limitation
BAL+LA+BB+X	Moderate	Study limitation
B. lactis UABla-12	High	NA
LP+LC+BL+ST+LA+X	Low	Study limitation
EF+LA+X	Low	Study limitation; Imprecision
LA+LR+ST+BL+BBR+X	Moderate	Study limitation
L. acidophilus ATCC 700396	High	NA
BAL+LA+LR+X	Moderate	Study limitation
S. boulardii	Low	Study limitation; Imprecision
C. butyricum	Moderate	Study limitation
BAL+ST+LB	Moderate	Study limitation
B. infantis 35624	Low	Study limitation; Imprecision
LPA+LA+BAL	Moderate	Study limitation
Ranking	Moderate	Study limitation

8. Bowel movement frequency (per week) in IBS-D

Comparison	Confidence	Downgrading due to
EF+LA+X	Moderate	Study limitation
L. paracasei B21060	High	NA
L. paracasei HA-196	High	NA
B. coagulans GBI-306086	Moderate	Study limitation
L. plantarum Apsulloc 331261	Moderate	Study limitation
B. longum R0175	High	NA
LP+LC+BL+ST+LA+X	Moderate	Study limitation
C. butyricum	Low	Study limitation; Imprecision
S. cerevisiae CNCM I-3856	High	NA
S. boulardii	Low	Study limitation; Imprecision
Ranking	Moderate	Study limitation

9. Bowel movement frequency (per week) in IBS-C

Comparison	Confidence	Downgrading due to
B. bifidum MIMBb75	High	NA
LA+ST+X	Moderate	Study limitation
S. cerevisiae CNCM I-3856	High	NA
L. paracasei HA-196	High	NA
B. coagulans Unique IS2	Moderate	Study limitation
B. longum R0175	High	NA
BAL+ST+LB	Low	Study limitation; Imprecision
Ranking	Low	Study limitation

10. Bristol stool form scale in IBS-D

Comparison	Confidence	Downgrading due to
B. coagulans MTCC 5856	Low	Inconsistency
S. cerevisiae CNCM I-3856	Low	study limitation; inconsistency
BL+LR	Low	study limitation; inconsistency
LA+LR+ST+BL+BBR+X	Moderate	study limitation
LP+LP+PA	Low	study limitation; inconsistency
L. paracasei HA-196	High	NA
B. bifidum MIMBb75	High	NA
VSL#3	Moderate	study limitation
C. butyricum	High	NA
B. longum R0175	High	NA
LP+LC+BL+ST+LA+X	Moderate	study limitation
S. boulardii	Moderate	study limitation
Ranking	Moderate	Study limitation

11. Bristol stool form scale in IBS-C

Comparison	Confidence	Downgrading due to
L. paracasei HA-196	High	NA
S. cerevisiae CNCM I-3856	Moderate	Study limitation
B. longum R0175	High	NA
BAL+ST+LB	Low	Study limitation; Imprecision
Ranking	High	NA

Table S8. Alterations of gut microbiota abundance.

Study	Probiotic used	Alterations of gut microbiota in probiotic groups
Bai, 2023	B. infantis, L. acidophilus, E. faecalis, and B. cereus	↑Butyricimonas, Pseudobutyrivibrio, Barnesiella, and Sutterella
Jung, 2022	L. plantarum	↑Firmicutes，Lactobacillus<br>↓Bacteroides
Xu, 2021	L. casei, B. animalis subsp. lactis, and L. plantarum	↑Prevotella (days 28)<br>↓Prevotella (days 7), Citrobacter, Clostridium, Escherichia
Liu, 2021	L. plantarum	↑Bifidobacterium, Bacteroides, Ruminococcus, Parabacteroides,<br>↑Butyric acid-producing genera: Anaerostipes, Anaerotruncus, Bifidobacterium, Butyricimonas, and Odoribacter<br>↓Prevotella, Methanobrevibacter (bloating-related genus)
Lewis, 2020	L. paracasei and B. longum	→ L. paracasei, B. longum or Bifidobacterium
Sun, 2018	C. butyricum	↓Clostridium sensu stricto
Shin, 2018	L. gasseri	↑Actinobacteria, Lactobacillus, Bifidobacterium<br>↓Proteobacteria, Blautia, fecalibacterium
Cremon, 2018	L. paracasei	↑Lactobacillus, Oscillospira, Parabacteroides, and an unidentified member of the family Barnesiellaceae<br>↓Ruminococcus
Nobutani, 2017	L. gasseri	↓Dorea, Enterococcus and Dialister
Hod, 2017	L. rhamnosus, L. casei, L. paracasei, L. plantarum, L. acidophilus, B. bifidum B. longum, B. breve, B. infantis, S. thermophilus, L. bulgaricus, and L. lactis.	↑Bifidbacterium, Lactobacillus and Streptococcus genera
Mezzasalma, 2016	L. acidophilus, L. reuteri, L. plantarum, L. rhamnosus, B. animalis subsp. Lactis	↑L. acidophilus<br>↓B. animalis subsp. lactis
Yoon, 2015	B. bifidum, B. lactis, B. longum, L. acidophilus, L. rhamnosus, and S. thermophilus	↑B. bifidum, B. lactis, L. acidophilus, L. rhamnosus<br>→ Bacteroidetes and Firmicutes
Yoon, 2014	B. bifidum, B. lactis, B. longum, L. acidophilus, L. rhamnosus, and S. thermophilus	↑B. lactis, L. rhamnosus and S. thermophilus<br>→ B. longum, B. bifidum, L. acidophilus, and Escherichia coli subgroup, and Clostridium perfringens and Bacteroides group.
Lee, 2013	L. rhamnosus, B. animalis subsp. lactis, L. casei, L. acidophilus and L. fermentum	↓Clostridium
Charbonneau, 2013	B. infantis	↑B. infantis, Clostridium coccoides-Eubacterium rectale group
Cui, 2012	B. longum and L. acidophilus	↑Bifidobacterium and Lactobacillus
Michail, 2011	B.longum, B. infantis, B. breve, L. acidophilus, L. casei, L. delbrueckii ssp. bulgaricus , L. plantarum,and S. salivarius ssp. thermophilus.	→ No differences noted at the Phylum, Class, Order, Family, or Genus levels.

↑: bacteria with increased abundance; ↓: bacteria with decreased abundance; →: bacteria with no significant change.

Table S9. The alteration of gut microbiota diversity before and after intervention

Study	Probiotic used	α diversity	β diversity
Bai, 2023	B. infantis, L. acidophilus, E. faecalis, and B. cereus	1. No significant changes in α diversity (Chao1, ACE, Shannon, and Simpson)	1. No significant changes in β diversity (measured by Bray Curtis and unweighted UniFrac distance)
Xu, 2021	L. casei, B. animalis subsp. Lactis, and L. plantarum	1. no significant difference was observed in the Shannon index between the control and probiotic groups at the end of the trial<br>2. the Shannon index increased in the probiotic group (days 7), followed by a slight decline afterwards (days 28)	1. obvious clustering pattern was seen on the PCoA score plots at day 7 and day 28 between two groups.
Liu, 2021	L. plantarum	1. The Simpson index and Shannon index of patients in the L. plantarum CCFM8610 group were significantly higher than those in the placebo group	1. supplementation with probiotics could change the composition of the gut microbiota (PCA)
Sun, 2018	C. butyricum	1. No significant differences were found between baseline and week 4 levels with Chao, Sobs and Shannon index<br>2. Sobs index showed an increased tendency after treating with CB	1. a significant difference was found at week 4 between two groups （PcoA analysis)
Cremon, 2018	L. paracasei	1. The differences between LCDG and placebo in modulating α diversity were not significant (Chao1, Shannon, and InvSimpson indexes)	1. The differences between LCDG and placebo in modulating β diversity were not significant (PCoA)
Pinto-Sanchez, 2017	B. longum	1. There were no major differences in alpha diversity (Shannon, Chao1, and Observed Species indices) before or after the treatment between the 2 groups.	1. There were no major differences in taxa, compositional distance (Bray-Curtis Principal Coordinate analysis) before or after the treatment between the 2 groups.
